# Single-cell analysis of cell fate bifurcation in the chordate *Ciona*

**DOI:** 10.1186/s12915-021-01122-0

**Published:** 2021-08-31

**Authors:** Konner M. Winkley, Wendy M. Reeves, Michael T. Veeman

**Affiliations:** Division of Biology, Kansas State University, Manhattan, KS 66506 USA

**Keywords:** *Ciona*, Ascidian, Single-cell RNAseq, FGF, MAPK, Cell state transition

## Abstract

**Background:**

Inductive signaling interactions between different cell types are a major mechanism for the further diversification of embryonic cell fates. Most blastomeres in the model chordate *Ciona robusta* become restricted to a single predominant fate between the 64-cell and mid-gastrula stages. The deeply stereotyped and well-characterized *Ciona* embryonic cell lineages allow the transcriptomic analysis of newly established cell types very early in their divergence from sibling cell states without the pseudotime inference needed in the analysis of less synchronized cell populations. This is the first ascidian study to use droplet scRNAseq with large numbers of analyzed cells as early as the 64-cell stage when major lineages such as primary notochord first become fate restricted.

**Results and conclusions:**

We identify 59 distinct cell states, including new subregions of the b-line neural lineage and the early induction of the tail tip epidermis. We find that 34 of these cell states are directly or indirectly dependent on MAPK-mediated signaling critical to early *Ciona* patterning. Most of the MAPK-dependent bifurcations are canalized with the signal-induced cell fate lost upon MAPK inhibition, but the posterior endoderm is unique in being transformed into a novel state expressing some but not all markers of both endoderm and muscle. Divergent gene expression between newly bifurcated sibling cell types is dominated by upregulation in the induced cell type. The Ets family transcription factor Elk1/3/4 is uniquely upregulated in nearly all the putatively direct inductions. Elk1/3/4 upregulation together with Ets transcription factor binding site enrichment analysis enables inferences about which bifurcations are directly versus indirectly controlled by MAPK signaling. We examine notochord induction in detail and find that the transition between a Zic/Ets-mediated regulatory state and a Brachyury/FoxA-mediated regulatory state is unexpectedly late. This supports a “broad-hourglass” model of cell fate specification in which many early tissue-specific genes are induced in parallel to key tissue-specific transcriptional regulators via the same set of transcriptional inputs.

**Supplementary Information:**

The online version contains supplementary material available at 10.1186/s12915-021-01122-0.

## Background

The journey from the totipotent fertilized egg to the myriad distinct cell types found in mature animals involves a series of decision points in which cells can follow different trajectories of differentiation [1]. It is now known that these cell fate bifurcations sometimes involve the asymmetric inheritance of cell fate determinants between sibling cells, but more often involve inductive interactions between different cell types. Decades of work in various model organisms have mapped out inductive interactions between many different cell types, identified specific signal transduction pathways used to induce specific cell fates, and identified important transcription factors controlling cell type-specific gene expression. Until recently, however, the global transcriptomic changes underlying bifurcations in cell fate have been elusive. RNAseq can be used to transcriptionally profile cell populations purified by FACS, but it is hard to scale this strategy to profile the full complexity of cell types in even simple developing embryos. Also, the maturation times for fluorescent markers of cell fates make it hard to purify distinct cell types until well after they are first specified. With the advent of massively parallel single-cell RNA sequencing (scRNAseq) [2–6], however, it is now possible to reconstruct cell type-specific transcriptional profiles from heterogenous cell mixtures including entire dissociated embryos. scRNAseq has been used to produce atlases of cell type-specific gene expression at key stages in several model organisms [7–10].

### *Ciona* as a model for understanding cell state transitions

In order to quantify the transcriptomic changes associated with cell fate bifurcation events, it is necessary to identify mother-daughter-sibling relationships between scRNAseq clusters. Here we use the mother-daughter-sibling terminology to refer to relationships between transcriptional states as one cell type bifurcates into two. This will vary in different contexts in how congruent it is with the actual lineages of cell divisions. Identifying these relationships de novo presents a complicated technical and conceptual challenge and has been the primary focus of many scRNAseq studies [11–15]. This is especially true in systems where differentiation is not synchronized and pseudotime inference strategies are needed to reconstruct developmental trajectories [16, 17] and infer the earliest transcriptomic changes between diverging cell types. Pseudotime inference involves major assumptions and there are non-trivial concerns that it may fail to accurately reconstruct cell state transitions [18, 19]. In the invertebrate ascidian chordate *Ciona*, however, the early lineages are completely stereotyped (Additional File 1: Figure S1), development is highly synchronous, and extensive fate mapping and traditional gene expression studies provide extensive and near-comprehensive prior information about the expected cell types and lineage relationships between them as reviewed in [20]. This allows highly synchronized sibling cell types to be captured for scRNAseq within the first cell cycle of their division from common progenitors. The transcriptomic differences between newly divergent cell types can thus be explicitly assessed within 20–30 min of their birth and without the underlying assumptions of pseudotime inference.

*Ciona* embryos are small and simple, yet stereotypically chordate. This makes relatively deep coverage of each cell type for an entire chordate embryo easily achievable in single-cell experiments. Most ascidian blastomeres become restricted to a single fate during a narrow window between the 64-cell and mid-gastrula stages [21–23], allowing the analysis of many cell fate specification events with a relatively short time course of sequenced stages. The mid-gastrula stage is reached in only ~ 6 h with most blastomeres having gone through 8 cell cycles. Development from the fertilized egg to the hatched tadpole larva takes less than 24 h. Several other studies have used scRNAseq to address diverse questions in ascidian models [10, 24–28], but here we provide the first scRNAseq analysis of the critical 64-cell to mid-gastrula time period in the widely used model ascidian *Ciona robusta*. This is also the first study in any ascidian to use high cell number droplet scRNAseq as early as the 64-cell stage.

The early patterning of the bilaterally symmetrical ascidian embryo up to the 32-cell stage relies primarily on two intersecting patterning systems. The first involves cortical rearrangements of the ooplasm and polarity breaking events in response to sperm entry, which culminate in the formation of a structure known as the centrosome-attracting body (CAB) in the posterior vegetal blastomeres at the 8-cell stage [29]. The CAB is then continuously partitioned into the posteriormost vegetal cells during subsequent asymmetric divisions and is thought to be responsible for directly or indirectly driving most of the anterior-posterior patterning of the early embryo (reviewed in [30]). The second system involves reciprocal interactions between maternally deposited Gata.a factors and nuclear β-catenin signaling activated on the vegetal side of the early embryo. In subsequent rounds of division at the 16-cell and 32-cell stages, these two pathways lead to the establishment of the three germs layers through antagonistic gene expression and restricted domains of nuclear localization [31–34].

At the 32-cell stage, only a subset of the endodermal blastomeres are restricted to a single fate, but nearly all the remaining blastomeres become fate restricted in the next two cell cycles [21–23]. The majority of the fate bifurcation events that take place in this time window are dependent on MAPK signaling [35–40]. MAPK activity at these stages is controlled by FGF ligands expressed on the vegetal side of the embryo downstream of β-catenin [41, 42], and by the FGF antagonist ephrinAd, which is expressed in the animal hemisphere downstream of Gata.a [38]. As development continues through the 112-cell and mid-gastrula phases, other FGF agonists and antagonists become expressed in other lineages such as the trunk lateral cells, and in complex patterns in the neural plate [43].

MAPK signaling directly induces several cell fates but also has well-characterized indirect effects. MAPK signaling induces expression of a Nodal ligand in a lateral animal cell population [44]. The Nodal signal activates downstream expression of Notch pathway ligands in neighboring cells and this Nodal/Delta relay further refines several tissue types at the 112-cell and mid-gastrula stages. This is particularly evident in the mediolateral patterning of the neural plate [44].

MAPK activity and repression are thought to be either directly or indirectly responsible for nearly all the fate bifurcations occurring after the 32-cell stage in the *Ciona* embryo [36, 38, 39, 43, 45, 46]. We performed whole-embryo scRNAseq at three key stages during cell fate specification both with and without the MEK inhibitor U0126. Using this data, we set out to characterize the repertoire of transcriptomic states in the early *Ciona* embryo and to define the transcriptomic responses of different precursor cells in response to FGF/MAPK signaling. We exploited the fixed lineages of *Ciona* to ask broad, systems-level questions about the gene regulatory networks (GRN) that control cell fate specification events, with an emphasis on inferring new features of the GRN controlling notochord specification and differentiation.

## Results and discussion

### Single-cell RNAseq of *Ciona* embryos recapitulates known expression patterns and reveals new transcriptional states

**Fig. 1:**
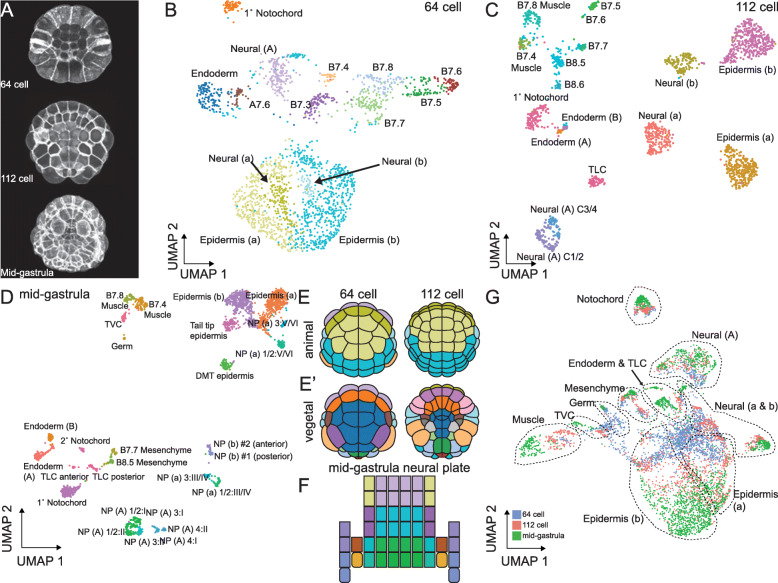
*Ciona* scRNAseq at the 64-cell, 112-cell, and mid-gastrula stages. **A** Z-projections of confocal images of embryos at each stage analyzed. Anterior is up in all images. **B–D** UMAP plot of STAMPs in control embryos dissociated at the 64-cell (**B**), the 112-cell (**C**), and the mid-gastrula (**D**) stages. NP—neural plate, NP X:Y represents the cell in row X and column Y of the neural plate. **E** Cartoon representations of the animal and vegetal views of the embryo at the 64-cell and 112-cell stages. Colors correspond to the stage matched UMAP plot in **B** and **C**. **F** Cartoon depiction of the neural plate at the mid-gastrula stage. Colors in **F** match the corresponding points in **D**. **G** UMAP plot of all control STAMPs collected across all three developmental stages. Dashed lines delineate broad lineages of the embryo

We performed single-cell RNA sequencing of dissociated *Ciona* embryos using a modified dropSeq approach [3] at the 64-cell, 112-cell, and mid-gastrula stages (stages 8, 10, and 12 in Hotta’s *Ciona* staging series [47]) (Fig. 1A and Additional File 1: Fig. S1). These stages are each spaced less than an hour apart at 18 °C and span a period in which most *Ciona* blastomeres become restricted to a single major fate. In order to obtain enough cells for each timepoint to ensure deep coverage of each left-right blastomere pair, we pooled gametes from multiple hermaphroditic adults for each fertilization. The small and well-defined number of bilaterally symmetrical cell pairs at these stages provides an upper limit on the number of distinct cell types that are theoretically possible. We profiled over 6000 cells in wild-type embryos across the three stages and achieved an average coverage of 42 times per theoretical blastomere pair. Most cell types are made up of more than one blastomere pair, so actual coverage of distinct cell types was typically higher. The scRNAseq libraries were sequenced to an average depth of 23,985 reads per cell, representing an average of 11,706 transcripts detected in each cell (Full sequencing statistics in Additional File 2: Table S1). Upon initial post-sequencing analysis and clustering, we generated first-pass UMAP plots. Each point on the UMAP plots represents a single set of 3′ RNAs associated with a unique cell barcode (also known as a “single-cell transcriptome attached to microparticle” (STAMP)), and the distances between points represent similarities and differences in gene expression across the entire transcriptome. The distinct clusters evident on the UMAP plots represent different cell types with their own distinct transcriptional states. We compared the gene expression profiles of the STAMPs contained within each cluster to those already known from extensive in situ hybridization screens in *Ciona* [48–50]. We identified several duplicate clusters for many cell types (Additional File 3: Fig. S2A-C). These were unexpected based on previous *Ciona* literature, and upon further inspection, we found that the number of duplicate clusters typically matched the number of *Ciona* adults used in each experiment.

A recently published *Ciona* single-cell RNAseq study near the time of zygotic genome activation at the 16-cell stage found that single-cell transcriptome profiles tended to cluster not by cell type, but by embryo-of-origin [26]. As that study only profiled 4 hand-dissected embryos, each of which was from a different mother, they were unable to determine if these effects were due to differences between mothers of origin or individual embryos. To determine if differential deposition of maternal RNA between the different adults used in each experiment could be driving our observed duplication of cell type clusters, we took advantage of *Ciona*’s relatively high rate of polymorphism [51] and called SNPs across the entire genome for each STAMP in our data. We calculated a metric of relatedness between all pairwise comparisons of STAMPs [52] at each stage and hierarchically clustered the resulting relatedness matrix. The dendrograms obtained from clustering at each stage contained several long branches, which matched the number of adults used in each experiment (Additional File 3: Fig. S2D-F). We then mapped the putative maternal identity back on the UMAP plots and found that the STAMPs tended to be clustering primarily by their mother at early stages, with this effect diminishing at later stages (Additional File 3: Fig. S2G-I). This trend is consistent with expectations if differences in maternally deposited RNAs are contributing the majority of the variance between STAMPs early on and if this effect is “washed out” over time as zygotic transcription products accumulate in the cells. We then used the variable regression capability of the SCTransform function in Seurat [53, 54] to regress out the mother-of-origin effects and allow cells to cluster based strictly on cell type-specific gene expression.

**Fig. 2:**
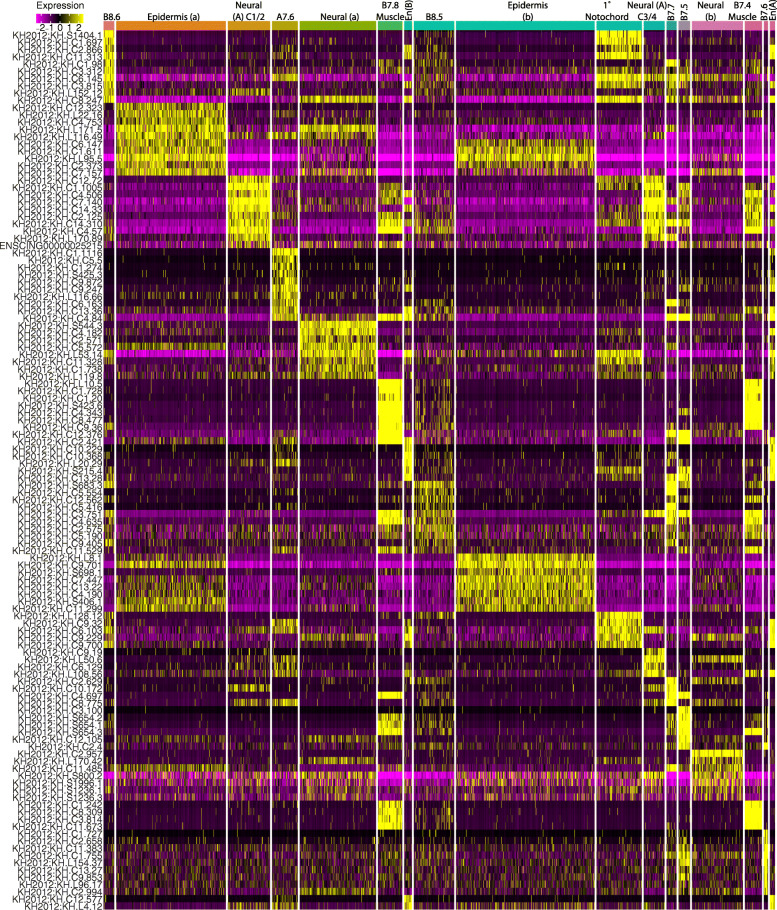
Marker genes of all cell types at the 112-cell stage. All cells are shown clustered into their assigned cell types. The union across clusters of the top 10 marker genes as ranked by Log2 fold-change for each cell type are shown. Color scale represents depth-corrected log-scaled expression value in each cell

Following post-sequencing SNP analysis and computational clustering, we again generated UMAP plots for each stage (Fig. 1B–D). This time we were able to assign cell identities to the scRNAseq clusters at all three stages, and the number of clusters was now in line with expectations based on extensive prior gene expression profiling (Fig. 1E, F, Additional File 2: Table S2, Fig. 2, Additional File 4: Fig. S3, Additional File 5: Fig. S5). Some of these clusters represented broad territories of the early embryo such as the presumptive anterior or posterior epidermis, but others had single blastomere-pair resolution such as the lateral columns of the posterior neural plate at the mid-gastrula stage. Expression patterns of the most highly variable transcription factors in the 112-cell stage clusters are generally similar to their previously characterized expression patterns by in situ hybridization but revealed important quantitative dynamics to expression patterns that had previously been appreciated only in a binary ON/OFF framework (Fig. 2 and Additional File 2: Table S3).

### New cell transcriptional states

**Fig. 3:**
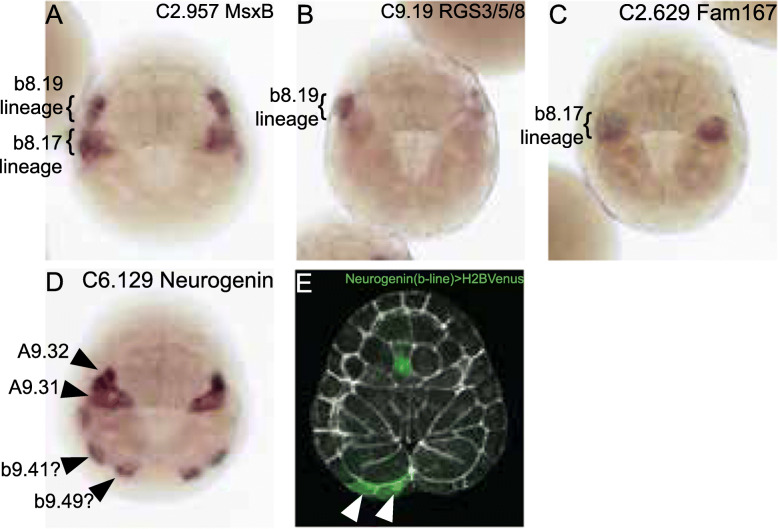
Validation of novel cell states. **A–D** In situ hybridation at stage 12 for the indicated markers. **A** *MsxB* is expressed throughout the b-line neuromesodermal territory and is included as a landmark. It is also expressed in the sibling dorsal midline tail epidermal cells but this expression is more lateral and cannot be clearly seen in this dorsal view. **B** *RGS3/5/8* is expressed in the more anterior b8.19 lineage. **C** *Fam167* is expressed in the more posterior b8.17 lineage. **D** *Neurogenin* was consistently expressed in posterior marginal b-line cells believed to be b9.41 and b9.49. It is also expressed in its well-characterized pattern in A9.32 and A9.31. There was occasional expression in a cell just anterior to b9.41 likely to be part of the dorsal midline tail epidermal precursors that are known to express neurogenin at a slightly later stage [58]. **E** Stage 12.5 embryo electroporated with a neurogenin (b-line) reporter construct driving expression of a Histone H2B Venus fluorescent protein fusion. Mosaic expression is seen in two posterior marginal blastomeres believed to be b9.41 and b9.49 (white arrowheads). Reporter signal is shown in green and phalloidin in white. The embryos in all panels are oriented with anterior to the top and the vegetal pole towards the viewer

In addition to recapitulating almost all of the cell types expected at these stages based on the *Ciona* fate map and gene expression databases, we also identified three previously unappreciated transcriptional states in the mid-gastrula embryo: a cluster of tailtip epidermal precursors and two distinct cell states within the b-line neural lineage. To validate these clusters and map them back onto the embryo, we performed in situ hybridizations with predicted markers. The b-line neural lineages at the canonical mid-gastrula stage consist of two anterior cells, b9.38 and b9.37 (dorsal neural tube in the posterior trunk and tail), and two more posterior cells, b9.33 (uncertain fate potentially including dorsal tail nerve cord) and b9.34 (secondary muscle) [21, 55–57]. One of the two b-neural clusters was enriched for the early muscle markers Tbx6c and MyoD, so we predicted this would consist of b8.17 lineage cells, and the other cluster would represent the more anterior b8.19 lineage. In situ hybridization for markers of these two clusters confirmed these expression patterns (Fig. 3). Patterning of b-line neural cells is not well understood, so these new clusters provide an entry point for future studies.

Hox12 and Wnt5 enrichment in the unexpected posterior epidermal cluster suggested that it would consist of cells fated to the posterior tip of the tail [49, 59]. This cluster was also strongly enriched for neurogenin. Neurogenin expression has been previously noted in posterior b-line cells, but the specific blastomeres involved were not clear [58, 60]. Fatemapping at the 112-cell stage shows that tail tip epidermal fates are derived from the posterior marginal b-line cells b8.21, b8.25, and b8.27 [37]. We used in situ hybridization and also a reporter construct for neurogenin [58] (Fig. 3) to show that this putative tail tip epidermal cluster most likely consists of b9.41 and b9.49, which are the marginal daughters of b8.21 and b8.25. The posteriormost marginal b-line cell b9.53, which is fated to the posterior ventral midline, is excluded from this expression domain. Many early cell fate decisions in the early *Ciona* embryo involve signaling interactions between the animal and vegetal hemispheres, so it is certainly reasonable that early manifestations of tail epidermal patterning should involve b-line cells at the animal-vegetal margin.

### Linking cell states across time

*Ciona* embryos have stereotyped and intensively studied cell lineages, so the parent/daughter/sibling relationships between the cell states represented by different STAMP clusters are straightforward to infer. We first assigned cell types to each STAMP cluster separately at each stage. To confirm the consistency of these assignments between stages, we clustered the STAMPs from all three of our stages in the same reduction of high-dimensional gene expression space. The UMAP plot from this reduction represented the known lineages in *Ciona* embryos relatively well (Fig. 1G and Additional File 1: Figure S1). The STAMPs from a given lineage across stages tended to cluster close together in a pattern that radiated outwards from the center of the UMAP plot with STAMPs that were later in developmental time usually being further from the center (Fig. 1G). This generally confirmed that our cell type assignments were consistent between lineages and showed an overarching trend that cell types established at the 64-cell stage or earlier tend to become far more transcriptionally divergent over time.

### Most but not all FGF-dependent cell types are transfated to sibling cell types upon MAPK inhibition

**Fig. 4:**
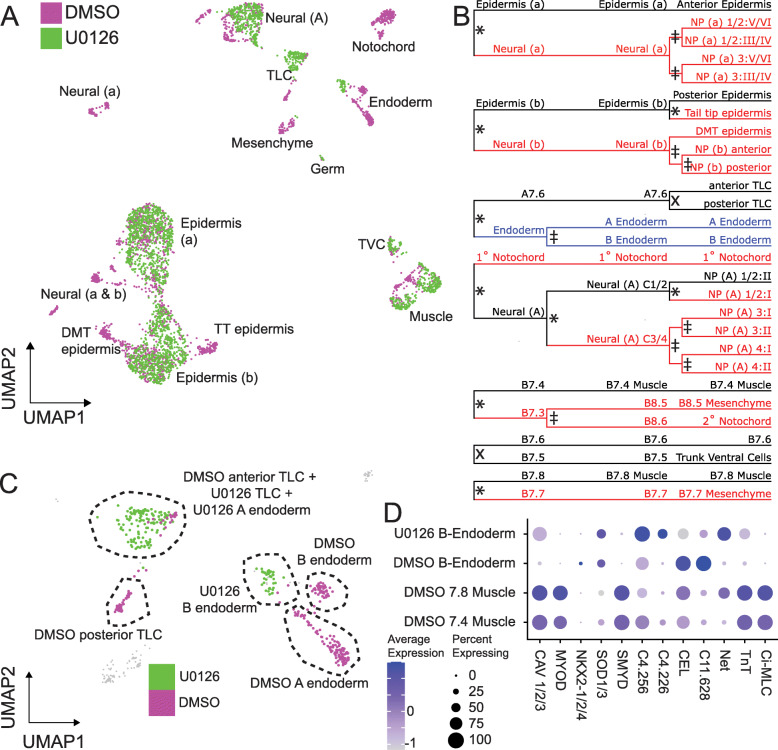
MAPK inhibition eliminates many cell types. **A** UMAP plot of all DMSO and U0126 treated STAMPs at the mid-gastrula stage. **B** Lineage diagram of all STAMP clusters identified in control embryos at the 64-cell, 112-cell, and mid-gastrula stages. Red lineage branches are missing after treatment with U0126, black lineage branches remain after U0126 treatment, blue lineage branches are transfated to a unique state. * = *p* < 0.05, X = *p* > 0.05, ‡ = untestable. **C** UMAP plot of endoderm and TLC STAMPs at the mid-gastrula stages depicting the incomplete transfating of B-line endoderm. **D** Expression of endoderm and muscle markers in the U0126 treated B-line endoderm, DMSO-treated B-line endoderm, and DMSO-treated muscle lineages

To better understand the transcriptional responses to FGF/MAPK signaling, we performed these scRNAseq experiments in parallel with control embryos and embryos treated with the MEK inhibitor U0126 from the 16-cell stage. Treatment with U0126 at this timepoint is known to block induction of mesenchyme, endoderm, and notochord [36, 39, 40]. It also prevents neural induction in a-line and b-line animal blastomeres [46] and disrupts the mediolateral patterning of the A-line neural plate, which secondarily prevents the establishment of A-line secondary muscle fate [43, 44]. The effects on A-line neural plate mediolateral patterning are mediated through a Nodal/Delta signaling relay downstream of FGF/MAPK signaling [44]. This Nodal/Delta relay is also required for the fate bifurcation involving secondary notochord [45]. We found that most of the expected cell lineages are missing from the drug-treated embryos (Fig. 4A) and that sibling cell types are present in excess (Additional File 2: Table S2). In order to define a metric of FGF dependence for each bifurcation, we predicted cell types in the U0126 STAMPs using the cell type label transfer function from Seurat. These predictions were inconsistent for certain cell types at the 64- and 112-cell stages but aligned closely with expectations from published literature at mid-gastrula as sibling cell types become more divergent. We performed chi-square tests for whether drug treatment influences the proportion of cells in sibling clusters (using the STAMPs for all descendant lineages of a STAMP cluster at mid-gastrula as a proxy). We were not able to compute this metric for the bifurcations where the parent cluster was itself FGF dependent (Fig. 4B). The only testable bifurcation that was not significant was between B7.5 and B7.6 (Fig. 4B). The cell fate decision between the B7.5 trunk lateral cell precursor and its sibling B7.6 germ cell precursor is known to depend on maternal determinants segregating in this lineage [61], but there have also been suggestions that FGF signaling may additionally be involved [28, 50]. We see no evidence in our dataset for the B7.5 versus B7.6 bifurcation being FGF/MAPK dependent when assessed at these timepoints. B7.5 and B7.6 are clearly distinguishable and show similar patterns of gene expression in both DMSO- and U0126-treated embryos, including robust expression of the B7.5 marker MESP.

All of the FGF-dependent bifurcations except one were consistent with the FGF-dependent cell type being transfated to its sibling cell type. We detected one novel cluster at the mid-gastrula stage that was only made up of STAMPs from U0126-treated embryos and not control STAMPs (Fig. 4A,C). This cluster expresses some but not all markers of both endoderm and muscle and is thus likely to represent the B-line posterior endoderm (Fig. 4D). Previous studies indicate that MAPK inhibition leads to A-line endoderm being transfated to A7.6 trunk lateral cell precursor fate [39] but have been unclear on what becomes of the posterior B-line endoderm. In a different tunicate, *Halocynthia roretzi*, FGF inhibition has been reported to cause the posterior B-line endoderm to adopt a cousin-cell muscle fate in explants, but not in whole embryos [62]. We see the expected increase in STAMPS matching the A7.6 TLC precursor transcriptional profile, but the novel U0126 treated B-line cluster indicates that the lineages that normally form B-line endoderm are not transfated to sibling cell types but instead establish a new transcriptional state not seen in normal embryos. Maternal β-catenin is a well-established endoderm determinant in ascidians and is involved in germ layer segregation early in development [31, 32, 34]. One interpretation is that vegetal FGF signaling acting downstream of maternal β-catenin is required for the normal posterior endoderm transcriptional regime, but that β-catenin alone is sufficient to make the B6.1 posterior endoderm lineage persistently distinct from the B6.2 muscle/mesenchyme/2° notochord lineage. Regardless of the underlying mechanisms, this observation of a novel transcriptional state in MAPK-inhibited posterior endoderm presents a clear demonstration of a limit to canalization in this deeply stereotyped embryo.

With the exception of posterior endoderm, all the cell fate decisions analyzed appear to be deeply canalized when assessed at the mid-gastrula stage with expected cell types either present or absent. These relationships are more complex, however, when examined earlier. At the 64-cell stage in particular, the FGF-dependent cell clusters are missing but there are also U0126-treated cells visible on the UMAP plot that are not perfectly overlapping with the control FGF-independent cell types (Additional File 6: Fig. S5A-B). Cell type predictions in the U0126-treated cells based on the patterns of differential gene expression in DMSO control cells suggest that some MAPK-dependent cell types might be transiently distinguishable at early stages even after U0126 treatment (Additional File 2: Table S2), as proposed by [28]. That said, the accuracy of the label transfer functions used to make these predictions is not clear, and gene expression in these putatively distinguishable cell types is very different than in control embryos. An alternate explanation is that these observations might reflect slight differences in the rate or synchrony of development in DMSO-treated versus U0126-treated embryos. Additional experimental work including more closely spaced scRNAseq timepoints would be needed to distinguish between these possibilities.

### Elk1/3/4 as a putative autoregulatory TF in an FGF-dependent feedback loop

**Fig. 5:**
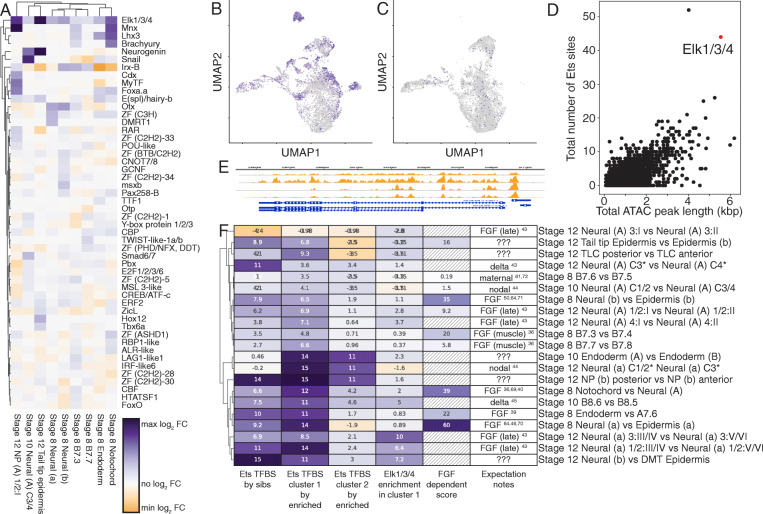
Elk1/3/4 is a putative feedback regulator of MAPK signaling. **A** Log2 fold-change of the expression level of the top 50 most variably expressed transcription factors in U0126 sensitive cell type clusters compared to their U0126 insensitive sibling cell type clusters. **B, C** Expression of Elk1/3/4 (purple colorscale) in DMSO (**B**) and U0126 (**C**) treated embryos. UMAP space is identical to Fig. 1G. **D** Scatter plot of sequence length and number of predicted Ets sites for all ATACseq predicted open chromatin regions. The ATACseq peak for Elk1/3/4 is highlighted in red. **E** Putative Elk1/3/4 sites in the 1500 bp upstream of the Elk1/3/4 transcription start site. The orange tracks show ATACseq open chromatin profiling at multiple stages from [63]. **F** Ets TFBS and Elk1/3/4 expression enrichment in sibling cell types distinguishes between bifurcations controlled directly versus indirectly by MAPK/ETS signaling. Ets TFBS by sibs—Ets TFBS enrichment score comparing the genes upregulated in the first sibling cluster compared to the genes upregulated in the second sibling cluster. Ets TFBS by enriched—Ets TFBS enrichment score comparing the genes upregulated versus downregulated in that particular cluster. Elk1/3/4 enrichment—Log2 fold-change in Elk1/3/4 expression in sibling cluster 1 compared to sibling cluster 2. FGF-dependent score—negative-log10(*p* value) of the chi-square test for association between sibling cluster numbers and treatment status, hatched boxes could not be measured due to loss of a precursor cell type. The negative log transformation makes the FGF dependency score viewable using the same color map as the other columns, but it was not included in the clustering. Expectation notes—expectation for signal dependency in each bifurcation based on the literature cited

For all of the FGF-dependent cell bifurcations, we asked to what extent these diverse lineages exhibit a universal FGF/MAPK transcriptional response versus lineage-specific responses. To address this, we clustered the fold-change in expression between FGF-dependent lineages and their FGF-independent siblings for the most variably expressed TFs. Most lineages exhibit their own characteristic patterns, but we noticed a striking and unexpected result that the Ets family transcription factor Elk1/3/4 is consistently upregulated in the FGF-dependent cell type compared to its sibling cell type (Fig. 5A). The FGF/RAS/MAPK/MEK signaling pathway culminates in *Ciona* and other animals in a transcriptional response largely mediated through Ets family transcription factors [64, 65], of which the *Ciona* genome contains 13 putative members. Published in situ expression patterns of Elk1/3/4 at these stages are complex and hard to interpret [49, 65], so this relationship could easily be missed without the quantitative data provided by scRNAseq. Elk1/3/4 expression is strongly MAPK dependent as demonstrated by its drastically reduced expression in U0126-treated embryos (Fig. 5B, C). This was previously noted in certain neural lineages [66], but is evident here throughout the embryo. We scanned for consensus Ets binding motifs in open chromatin regions throughout the *Ciona* genome using the early whole-embryo ATACseq data of [63] and found that the Elk1/3/4 locus is associated with a large amount of nearby open chromatin containing a very large number of predicted Ets sites (Fig. 5D, E). Elk1/3/4 expression is at least a proxy for Ets family transcriptional activity and given that Elk1/3/4 is itself an Ets family TF, it may have an important feedback or feedforward role in FGF/MAPK-driven cell fate specification.

### Inferring direct versus indirect roles for FGF/MAPK signaling in cell fate bifurcations

Most of the cell fate decisions in the 64-cell to mid-gastrula stages are either directly or indirectly dependent on MAPK signaling. To quantify the contribution of FGF/MAPK regulated ETS family TFs to these FGF-dependent cell states and make inferences about direct versus indirect roles, we performed transcription factor binding site (TFBS) enrichment analysis. TFBS enrichment analysis uses statistical models to determine if the binding site sequence for a particular TF is overrepresented in a set of target DNA sequences compared to a set of control DNA sequences [67]. In *Ciona*, most enhancers and other cis-regulatory modules are thought to be relatively close to the transcription start site [68]. We used the 1.5 kB of DNA upstream of the transcription start site to test for enrichment of Ets sites in the putative regulatory regions of genes that are upregulated in FGF-dependent cell types as compared to their sibling cell types. We also performed TFBS enrichment analysis in parallel using open chromatin regions inferred from whole-embryo ATACseq data [63] instead of the 1.5 kb upstream regions. Following these two analysis methods, we averaged the enrichment metric *z*-scores for each TFBS from each method to get a combined *z*-score of binding site enrichment in target sequences compared to control sequences. Our justification for averaging the results between these two target sequence sets is that neither approach is clearly superior for all genes. The 1.5 kb upstream regions will miss regulatory regions that are more distal or in introns, but the whole-embryo bulk ATACseq data is subject to ambiguities in peak calling and a strong bias towards chromatin regions that are open ubiquitously or in high abundance cell types. Averaging the enrichment *z*-scores provides a simple way of integrating information from both sets of putative regulatory regions.

*Z*-scores for the Ets family TFBS vary widely across the different FGF-dependent cell fate bifurcations, indicating that Ets-mediated transcription is likely to play a major role in some bifurcations and not in others (Fig. 5F, column 1). Divergent use of Ets sites between sibling cell types could potentially reflect upregulation of Ets-mediated targets in the induced cell type and/or repression of Ets-mediated targets in the uninduced sibling cell type. To address this, we again performed TFBS enrichment analysis, this time using the genes that are enriched in each cell type compared to all other cell types at a given stage to make up the target set and those that are depleted in the same comparison to make up the control set. This was performed for each bifurcation for both the FGF-dependent cell type (Fig. 5F, column 2) and its default sibling cell type (Fig. 5F, column 3). For bifurcations where MAPK dependency could not be tested because a precursor cell type was MAPK dependent, we ordered columns 2 and 3 based on expectations from the published literature for bifurcations that have previously been studied in detail and based on ETS site enrichment between the two sibling cell types for those that have not.

To identify distinct trends of Ets transcriptional responses directly or indirectly downstream of FGF/MAPK signaling, we performed hierarchical clustering of the enrichment *z*-scores for the Ets family TFBS calculated in the three different ways discussed above (Fig. 5F, columns 1–3). We also included differential expression of Elk1/3/4 between sibling cell types in the clustering given that we found it to be a likely proxy for Ets family TF activity (Fig. 5F, column 4).

While the quantitative details varied between bifurcations and they cluster in complex ways, the majority of bifurcations showed a consistent pattern in which ETS sites were enriched comparing upregulated genes in sib 1 vs sib 2 (column 1) and also comparing upregulated genes in sib 1 vs downregulated genes in sib 1 (column 2). These bifurcations also consistently showed enrichment of Elk1/3/4 in sib 1 (column 4). This signature of strong ETS site TFBS enrichment and Elk enrichment is consistent with MAPK signaling playing a direct role in these bifurcations. Numerous bifurcations thought to be directly induced by MAPK signaling are included in this group (primary notochord vs A-line neural, a-line neural vs anterior epidermis, b-line neural vs posterior epidermis, most row I vs row 2 neural fates) [36, 40, 43, 46, 50, 64, 69–71]. Other bifurcations believed to be independent of MAPK or controlled by indirect relay mechanisms did not show this signature. These included bifurcations induced by Nodal signaling (lateral column 3/4 vs medial column 1/2 fate in both A-line and a-line neural) [44] and bifurcations controlled by maternal determinants (B7.6 vs B7.5, anterior vs posterior endoderm) [61, 72].

While most of the bifurcations were consistent with expectations in terms of ETS TFBS enrichment, there were three bifurcations with unexpected results. The bifurcation between B8.6 (secondary notochord) and B8.5 (mesenchyme) is thought to be proximately induced by Delta/Notch signaling from A7.6 and only indirectly downstream of FGF/MAPK signaling via a relay mechanism involving the b-line neural lineage [45]. This bifurcation, however, shows strong enrichment of Ets TFBS sites in B8.6 and also strong enrichment for Elk1/3/4, suggesting that FGF/MAPK signaling might be more directly involved in this bifurcation in parallel to Delta2. In support of this hypothesis, we note that A7.6 strongly expresses FGF8/17/18 as well as Delta2 and that differential dpERK staining has been observed between B8.6 and B8.5 [45]. The combined FGF9/16/20 FGF8/17/18 double morpholino knockdown also has a more penetrant effect on secondary notochord cell fate than FGF9/16/20 knockdown alone [45], which is again consistent with a potential direct role of FGF8/17/18 from A7.6 in inducing B8.6 fate given that FGF8/17/18 is not expressed until after the early induction of b-line neural fate by FGF9/16/20.

A similar pattern of strong ETS TFBS site enrichment and Elk1/3/4 enrichment was also seen in the bifurcation of column 4 vs column 3 A-line neural fates. This bifurcation is similar to B8.6 vs B8.5 in that there is strong evidence that it requires Delta2 expression in A7.6 as part of an indirectly FGF-dependent relay mechanism [43]. As with B8.6, the cell type with Ets TFBS enrichment is in physical contact with A7.6 and it is plausible that FGF8/17/18 and Delta2 might be acting in parallel.

The other inconsistency in these results involves the bifurcation between A9.29 (column 3 row I neural plate) and A9.30 (column 3 row II neural plate). Row I vs row 2 A-line neural fate is thought be directly induced by FGF/MAPK signaling [43] and columns 1, 2, and 4 show the expected Ets site enrichment. The column 3 row 1 vs row 2 bifurcation, however, shows no evidence of Ets site enrichment. It remains to be determined whether this represents a failure of TFBS analysis to detect a clear signature of Ets site enrichment in this bifurcation or whether it represents some unknown complexity of MAPK signaling specific to column 3 A-line neural cells. The corresponding bifurcations for row I versus row II fate in column 1/2 and column 4 A-line neural cells showed only modest ETS site enrichment, so there may be lineage-specific differences in Ets site usage or the potential use of MAPK effector TFs with different binding motifs. The B7.3 versus B7.4 and B7.7 versus B7.8 bifurcations were similar in having only modest ETS site enrichment.

This analysis also reveals that several later bifurcations involving unknown mechanisms have patterns of Ets site usage and Elk1/3/4 enrichment which suggest that FGF/MAPK signaling might be playing a direct role. These include the subdivision of the early b-line neural lineage into b8.17/b8.19 (dorsal nerve cord and secondary muscle) and b8.18/b8.20 (dorsal midline epidermis), the subdivision of the b18.17/b18.19 cluster into anterior and posterior subdomains, and the induction of the tail tip epidermal cluster. In support of a direct role for MAPK signaling in this latter cell type, dpERK staining was previously observed in b9.41 and b9.49 in the stolidobranch ascidian *Halocynthia* [73].

### Analyzing the genome-scale dynamics of cell fate induction

**Fig. 6:**
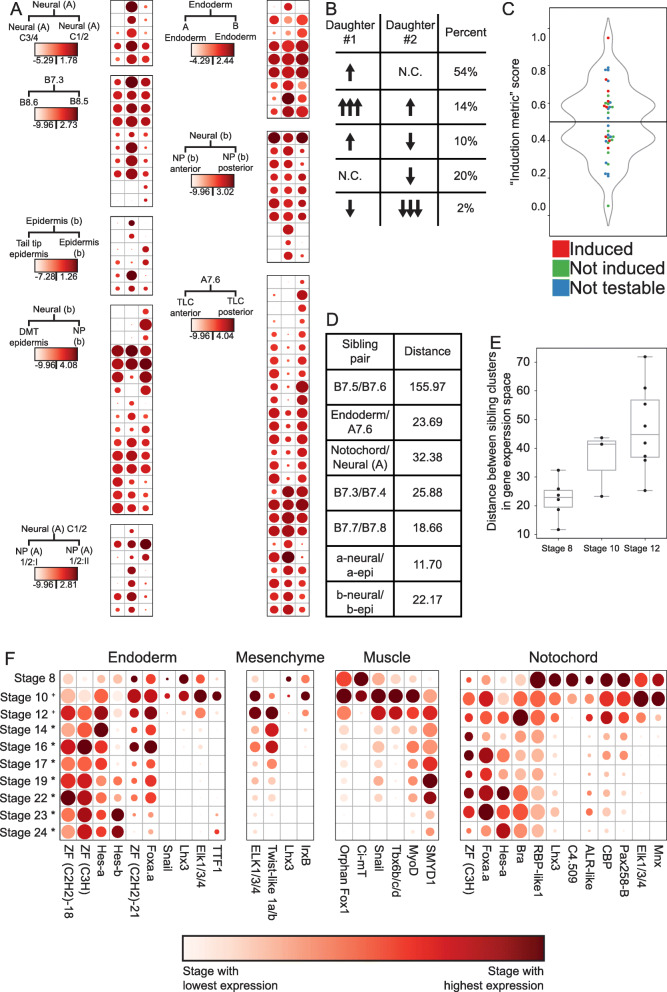
Genome-wide transcriptional responses during fate bifurcation events. **A** Log2 scaled expression levels (read counts corrected for sequencing depth) of differentially expressed transcription factors in “trios.” For each heatmap, the left column represents the parent cell type, the middle column is the “induced” sibling cell type, and the right column is the “uninduced” cell type. Color maps are scaled to each heatmap (See also supplemental table 4). Dot size represents the percent of cells expressing each gene as in Fig. 4D. **B** Different mechanisms of changes in expression level that lead to upregulation in daughter #1 compared to daughter #2, and percentages observed of each mechanism in our “trios.” **C** A simple “induction metric” (see text and methods) can be used to predict FGF sensitivity. Green = U0126 insensitive cell types by chi-square test, Red = U0126 sensitive cell types by chi-square test, Blue = untestable cell types by chi-square test (corresponds to Fig. 4B). **D** Distances between sibling cell types in gene expression space for each bifurcation occurring at the 64-cell stage. **E** Distances between sibling cell types in gene expression space for all bifurcations occurring at each stage (Germ lineage not included). **F** The expression level over time of TFs that are differentially upregulated in the endoderm, mesenchyme, muscle, and notochord lineages compared to their sibling cell types. Dot size represents the percent of cells expressing each gene

Our dataset’s wide coverage of cell types and stages enables a systematic analysis of the transcriptional changes underlying cell fate bifurcations. We first explored the expression changes of transcription factors in “trios” of STAMP clusters involving a mother cell type and its two daughter/sibling cell types. For transcription factors that passed a statistical test for differential expression between the two sibling cell clusters, we compared the expression level of that transcription factor in all three members of the trio (Fig. 6A and Additional File 2: Table S4). These dotplot-heatmaps reveal several consistent features. Numerous transcription factors are differentially expressed between the sibling cell states in each trio comparison. The majority of these were already expressed to some extent in the parental cell type, but some are newly expressed in one or both daughter/sibling clusters. One daughter typically has more upregulated TFs than the other (Fig. 6A).

If a transcript is upregulated in cell type A compared to its sibling cell type B, there are several possible underlying mechanisms (Fig. 6B). It could either be (1) upregulated in daughter A vs the parental cell state and unchanged in daughter B vs the parental state, (2) upregulated in both daughters vs the parental state but upregulated more in A than in B, (3) upregulated in daughter A vs the parental state and downregulated in daughter B vs the parental state, (4) unchanged in daughter A vs the parental state and downregulated in B vs the parental state, or (5) downregulated in both daughters versus the parental state, but downregulated more in daughter B than in daughter A.

We found that 54% of TFs that are differentially expressed between sibling cell types in a trio fall into the first category, 14% fall into the second, 10% into the third, 20% into the fourth, and the remaining 2% in the fifth category (Fig. 6B). The largest driver of differential expression between sibling lineages is upregulation in one sibling and not the other, but a surprising fraction of genes were upregulated in both siblings but more so in one than the other. This sort of differential upregulation has received little attention in the literature but was actually slightly more common (14% vs 10%) than the familiar situation in which a TF expressed in the parental state is upregulated in one sib state and downregulated in the other. Consistent with expectations for early embryonic cell fate decisions relatively soon after zygotic genome activation, upregulation was more common overall than downregulation. Individual TFs are seen to be upregulated in one or both sibs in 78% of the comparisons, whereas downregulation was seen in one or both sibs for only 32%.

The trio comparisons showed that one daughter typically had more upregulated transcription factors than the other, and we found that this was also true in the other bifurcations where we did not capture the parental cell state (Fig. S7). We hypothesized that this might reflect which daughter cluster in a cell fate bifurcation was induced downstream of FGF signaling. We tested this by calculating a simple metric of upregulated TF expression between sibling cell types involving the proportion of the total upregulation of differentially expressed TFs in each sibling cell type. This putative induction metric correctly predicted the induced cluster in 6 of the 9 bifurcations thought to be directly controlled by FGF/MAPK signaling (Fig. 6C). The exceptions were the B7.4 and B7.8 muscle lineages, which had more TF upregulation than their FGF-dependent mesenchyme and mesenchyme/2° notochord precursor sibling cell types, and also endoderm, which had more TF upregulation than A7.6. Intriguingly, all three of these bifurcations involve differential FGF/MAPK signaling being used within lineages influenced by major maternal determinants: macho-1/Zic-r.a [74] for muscle development and beta-catenin for endoderm [32]. We speculate that this differing trend in TF upregulation reflects the inhibition of intrinsically induced “default” fates versus the choice between alternate developmental trajectories in the absence of a strong maternal determinant.

### Newly born cell fates diverge from their sibling fates more quickly at later stages

The distances in gene expression space between sibling cell fates after bifurcation are widely variable across developmental time and anatomical regions. Some sibling cell types remain fairly close, indicating that gene expression profiles are not drastically changed between sibling cell states, whereas others are more divergent. The relatively transcriptionally silent B7.6 cell and its sibling blastomere B7.5 are far more distant from each other than other sibling cell state pairs at the same stage (Fig. 6D). This likely reflects maternal cell fate determinants known to segregate as RNA molecules into the B7.6 germ cell precursor downstream of patterning by the CAB [75]. We do however find a trend that the distance between sibling STAMP clusters at the first timepoint for which they are distinguishable becomes progressively greater over these three stages (Fig. 6E). This indicates that the rate of divergence in gene expression space between newly established sibling clusters is increasing with developmental time, and potentially reflects the establishment of new chromatin states and/or the zygotic expression of a broader set of transcription factors as development proceeds.

### Many cell fate bifurcations involve large numbers of differentially expressed transcription factors

Exploring the different cell fate bifurcations, we found that most involved a large number of differentially expressed TFs. Additional File 7: Fig. S6 is a poster-sized diagram detailing all of the differentially expressed transcription factors across all of the bifurcations analyzed. Decades of work in developmental genetics have emphasized the role of single transcription factors or small combinations of transcription factors in controlling specific cell fate decisions. Transcriptional profile comparisons between relatively well differentiated cell types invariably find that much larger suites of TFs are differentially expressed, but there are major questions about whether the earliest divergence between bifurcating cell types involves large or small sets of transcription factors. Here we found that many bifurcations involved 10 or more different TFs becoming differentially expressed between sibling cell types within an hour or less of cell division. This led us to explore the dynamics of these transcription factors over time to identify if they remained expressed in the tissue throughout development, or if they were only transiently upregulated at the time of differentiation. To address this, we used our scRNAseq dataset to identify all of the transcription factors upregulated in the earliest stages of muscle, mesenchyme, endoderm, and notochord differentiation as compared to their sibling cell types. We selected these cell types because they formed entire tissues at later stages of development without the dramatic increase in tissue subtypes exhibited in the neural lineages. Each of these tissues exhibited differential expression of a suite of several transcription factors (Fig. 6F). To infer patterns of transient versus stable expression, and to explore more of the temporal expression dynamics of these earliest diverging TFs, we also integrated our scRNAseq dataset of early *Ciona robusta* development with another single-cell RNAseq dataset focused on later *Ciona robusta* stages [10]. We compared the expression level from 64-cell through hatching larva of the TFs which were upregulated in muscle, mesenchyme, and endoderm compared to their sibling cell types at their initial bifurcation. We find that these TFs generally fall into one of two categories: some are expressed for only a short time window whereas others show persistent and/or increasing expression (Fig. 6F). Functional experiments are needed to determine which transcription factors diverging between sibling cell types have important roles in cell fate specification, but all of the bifurcations we have examined show several transcription factors becoming differentially expressed instead of a single putative master regulator. All of these newly specified cell types were captured by scRNAseq very soon after being separated from sibling lineages by cell division. They were typically on ice during the dissociation process within 15 min of the prior division and microfluidic encapsulation/lysis were underway within 35–40 min. It is thus likely that these relatively large suites of divergently expressed TFs represent direct responses to the earliest aspects of cell fate bifurcation.

### Many genes besides *Brachyury* are differentially expressed in the notochord immediately after fate bifurcation

**Fig. 7:**
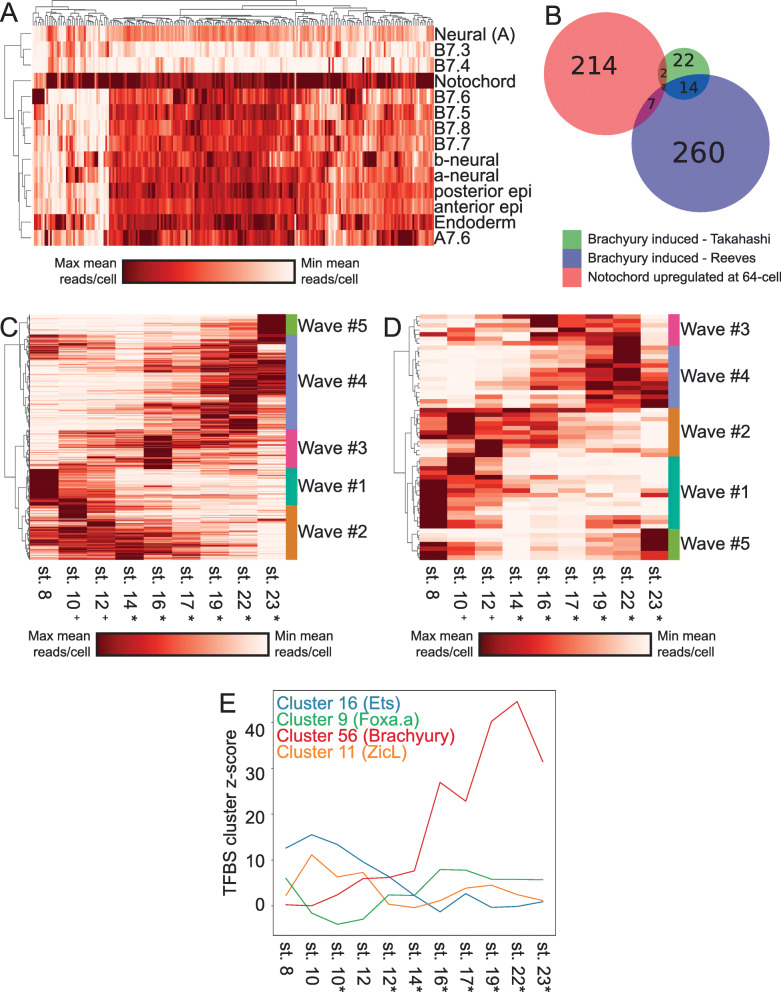
Waves of gene expression in the primary notochord. **A** Spatial expression profiles of all genes that are upregulated in the 1° notochord compared to their A-neural siblings at the 64-cell stage. **B** Overlap between the 1° notochord differentially upregulated gene set and two sets of Brachyury induced notochord-enriched genes from the indicated studies. **C, D** Temporal expression profiles of all notochord-enriched genes (**C**), and transcription factors (**D**) reveal distinct waves of expression throughout the course of development. **E** Enrichment *z*-scores for several TFBS families during the course of notochord development

The *Ciona* notochord has been intensively studied as a model for understanding tissue-specific gene expression and morphogenesis [76–85]. This is the first study to systematically identify differentially expressed genes in the *Ciona robusta* primary notochord compared to its sibling A-line neural cell fate immediately after cell fate induction at the 64-cell stage. To analyze if the differentially expressed primary notochord genes in our study were in fact notochord specific, we extracted the average expression value of each of these genes in all of the other cell clusters at the 64-cell stage. We found that nearly all of the genes are expressed most strongly in the notochord but many are also expressed elsewhere in the embryo to varying extents, indicating that they are notochord enriched, but not notochord specific (Fig. 7A). There are 12 transcription factors and many other genes that diverge between the 1° notochord and its A-neural siblings at this first timepoint immediately after they divided. Many of these genes have not previously been identified as notochord enriched at the 64-cell stage and may be effector genes of the earliest stages of the notochord GRN. There is very little overlap between the genes differentially expressed in the notochord at the 64-cell stage and those induced at later stages by ectopic expression of Brachyury in other studies (Fig. 7B) [86, 87], suggesting that these earliest differentially expressed genes may be expressed in parallel to, and not downstream of Brachyury. The transcription factors we found to be enriched in primary notochord as compared to A-line neural included several expected candidates such as Brachyury, Foxa.a, and Mnx [49, 50, 84, 88] as well as others such as Elk and Hes-a that have not been previously appreciated by in situ hybridization at the 64-cell stage. Much like in the endoderm, muscle, and mesenchyme, we find that the differentially expressed TFs are either expressed strongly in a short time frame around fate induction or are stably expressed and increase their expression over time (Fig. 6F).

### The notochord exhibits distinct waves of transcription

To further characterize the temporal dynamics of the *Ciona* notochord transcriptome, we plotted the notochord expression levels over time of all the genes that are enriched in the notochord compared to other non-notochord tissues at each stage. Hierarchical clustering of these notochord-enriched genes revealed 5 relatively distinct temporal waves of expression (Fig. 7C). Characteristic temporal expression profiles have previously been identified by in situ hybridization for select notochord genes, [78–80, 83, 84, 86, 89, 90] but the genome-wide transcriptional profiling performed here allows the comprehensive identification of distinct suites of genes that co-vary in expression in the notochord over time. A similar set of distinct temporal waves was seen when clustering the temporal expression profiles of the notochord-enriched transcription factors alone (Fig. 7D), suggesting that these waves might be controlled by distinct TFs or combinations of TFs acting in a differentiation cascade. All the waves of TF expression in the notochord involve suites of multiple co-varying TFs and not single genes. This suggests that the temporal dynamics of notochord-specific gene expression might represent a progression through a series of relatively distinct transcriptional states that each involves TFs acting in complex sets and not as single regulators. We performed Gene Ontology (GO) code enrichment analysis on the genes upregulated in the 5 primary notochord waves and found distinctive patterns in all 5 waves. The first wave is notably enriched for several GO codes related to cell cycle control, potentially related to the notochord’s imminent progression through its final mitotic divisions. The final wave is enriched for several GO codes related to cell membranes with potential relevance to notochord lumen formation (Additional File 2: Table S5). The secondary notochord also exhibits distinct transcriptional waves involving an overlapping but non-identical set of genes (Additional File 8: Fig. S7A-B). Future work will be needed to dissect these new GRN structures and test whether these waves of transcription coincide with, and possibly contribute to, the control of distinct morphogenetic events such as mediolateral intercalation and notochord elongation.

### The earliest notochord GRN is enriched for targets of Ets and Zic transcription factors

We used transcription factor binding site enrichment to infer potential roles for the numerous TFs enriched in the 1° notochord compared to its sibling cell type at the time of notochord induction. Using our scRNAseq dataset integrated with the partially overlapping Cao et al dataset [10], we performed TFBS enrichment analysis to identify which TF family binding sites are most enriched in the putative regulatory regions of genes enriched in primary notochord compared to other cell types at each stage. Our assumption from the current understanding of the notochord GRN is that the key notochord regulator Brachyury is induced by the combination of active Ets, Zic, and Foxa.a, and that most notochord-specific transcription should be directly or indirectly downstream of Brachyury. Interestingly, we find that the earliest targets are not enriched for Brachyury sites, but are instead highly enriched for the binding motifs of Zic and Ets family TFs (Fig. 7E and Additional File 9: Fig. S8A). Brachyury sites do not become enriched in notochord-enriched genes until much later in development, suggesting that there is a significant lag time between when it is first detectable at the RNA level by in situ at the 64-cell stage and when it becomes a major driver of notochord gene expression (Fig. 7E and Additional File 9: Fig. S8B). This supports the hypothesis that most early notochord genes are initially induced directly downstream of FGF, in parallel to and not downstream of the key notochord regulator Brachyury [86]. We also see that Foxa.a has a U-shaped TFBS enrichment profile over time (Fig. 7E), consistent with a role in a recently described feedforward network as both an upstream regulator of Bra expression and a major regulator of notochord-specific gene expression at later stages [86].

## Conclusions

### scRNAseq in an outbred, polymorphic species

Whole-embryo scRNAseq is a powerful new method for generating transcriptional atlases of distinct cell states and understanding the transitions between them. Here we found that a modest number of whole-embryo scRNAseq experiments has the power to recapitulate many, though by no means all, key findings from years of classical developmental biology experiments. We detected nearly all of the cell types expected from this simple and intensively studied embryo, but also identified previously undescribed transcriptional states that will facilitate studies of b-line neural patterning as well as the patterning of the posterior epidermis.

An unexpected complication in our dataset was the tendency of some cells to cluster at early stages based on mother of origin and not strictly by cell type. We introduced a method for removing these effects post hoc by inferring the mother-of-origin for each STAMP using SNPs and regressing out the maternal influence. This method exploits the extensive natural genetic variation in *Ciona* populations and will likely be useful for future scRNAseq studies in *Ciona* and other outbred, highly polymorphic species. SNP analysis could potentially be integrated with scRNAseq in a fecund, polymorphic species like *Ciona* in many interesting ways. STAMPS could potentially be assigned to distinct embryos to dissect variability on a per embryo basis. scRNAseq capture rates could be increased by overloading encapsulation devices and computationally identifying and removing cell doublets post hoc. This would be akin to the demuxlet algorithm currently used for multiplexed samples from different genotyped mouse or human samples [91] but now exploiting the much greater polymorphism of *Ciona* to infer post hoc genotypes for cells from thousands of different embryos resulting from mixing gametes from several hermaphroditic adults. Naturally occurring polymorphisms could also potentially be linked to transcriptional differences between genotypes.

### Global patterns of divergence between sibling cell types

*Ciona* and other tunicates are unusual among the chordates in having a modest repertoire of distinct cell types that become established via exceptionally stereotyped embryonic cell lineages. This makes it possible to achieve relatively deep coverage of most cell types in whole-embryo scRNAseq experiments using standard commercialized droplet microfluidics, and also to capture newly divergent sibling cell types within minutes of having divided from common parental cell types without the potentially problematic assumptions of pseudotime inference needed in the analysis of species where cell fate decisions are less synchronized and invariant. Our goal here was not just to create a transcriptional atlas of cell states during the late cleavage and early gastrula stages when most cells become restricted to a single predominant fate, but also to systematically analyze the genome-wide transcriptional changes in the cell fate bifurcations that establish these distinct cell types.

*Ciona* development is unusual in that a large number of cell fate decisions are controlled by only a few signaling pathways, with a particularly important role for FGF signaling on the vegetal side of the early embryo [20, 92]. A noteworthy feature of the cell fate bifurcations we profiled is that they were typically asymmetric in the sense that one sibling cell state had a greater number of upregulated transcription factors than the other. In most cases, the cell type with the greater number of upregulated TFs was the FGF-dependent cell type and not its FGF-independent sibling. This suggests that these inductive interactions are dominated by the upregulation of FGF target genes in the induced cell type. Interestingly, however, in the cases where we profiled parent-sib-sib cell state trios, the majority of TFs differentially expressed between the sibling cell types were already expressed to at least some extent in the parental cell type, indicating that the de novo expression of regulators unique to the induced cell state is not the dominant pattern.

Transcripts can become differentially expressed between sibling cell types through varying combinations of upregulation and downregulation in the two lineages. We found that the most common pattern was for a gene to be upregulated with respect to the parental state in one cell type while not changing in the sibling. All possible combinations were observed, however, and there were a surprising number of differentially expressed genes that were upregulated in *both* sibling cell types as compared to the parental cell type, but to varying extents. Note that differential transcript levels between sibling cell states might represent different underlying combinations of changes in transcription rates, decay rates, and asymmetric inheritance in cell division. The observation of strong TFBS enrichment signatures in many bifurcations supports the obvious role of direct transcriptional upregulation, but differential degradation and asymmetric inheritance could also be important in these very fast-paced cell state transitions.

Another noteworthy feature of the 17 bifurcations we examined is that later bifurcations were far more transcriptionally divergent than early bifurcations. We speculate that this might involve chromatin states becoming more open over time, and/or a larger set of zygotic transcription factors being expressed in parental cell states at later stages.

### *Ciona* super-enhancers?

We found that the Ets family transcription factor Elk1/3/4 was unique in being upregulated in almost all of the MAPK-dependent cell fate bifurcations, especially in the ones thought to be directly dependent on FGF signaling and not downstream relay mechanisms. Vertebrate Ets proteins generally act as transcriptional activators, but repressive functions have also been described [93]. It seems likely that *Ciona* Elk1/3/4 is acting in a feedback or feedforward loop, though it remains to be determined whether it is a positive or a negative regulator.

*Ciona elk1/3/4* is distinctive in being associated with more extensive regions of nearby open chromatin than all but two other *Ciona* genes, and also with a very high number of predicted Ets sites in these putative regulatory regions. Studies of mammalian genomes have led to the hypothesis that key cell fate specification genes are often under the cis-regulatory control of unusually large regulatory regions known as super-enhancers [94–96]. The super-enhancer concept has not previously been applied to studies of the small, compact *Ciona* genome, where many genes are known to be regulated by very short enhancer sequences [63, 68], but *elk1/3/4* suggests that the concept may be relevant. ChIP-seq data would be needed to formally test the mammalian super-enhancer criteria, but we find that transcription factors are enriched in the genes with more than 2 kb of associated open chromatin (Additional File 2: Table S6), and that the transcription factor loci with the most associated open chromatin include the effector TFs of many signal transduction pathways with roles in early *Ciona* patterning. These include Elk1/3/4 and another Ets factor, as well as RBPJ, a Lef/TCF, and a Smad (Additional File 2: Table S7).

### The “broad-hourglass” model of cell fate bifurcation

**Fig. 8:**
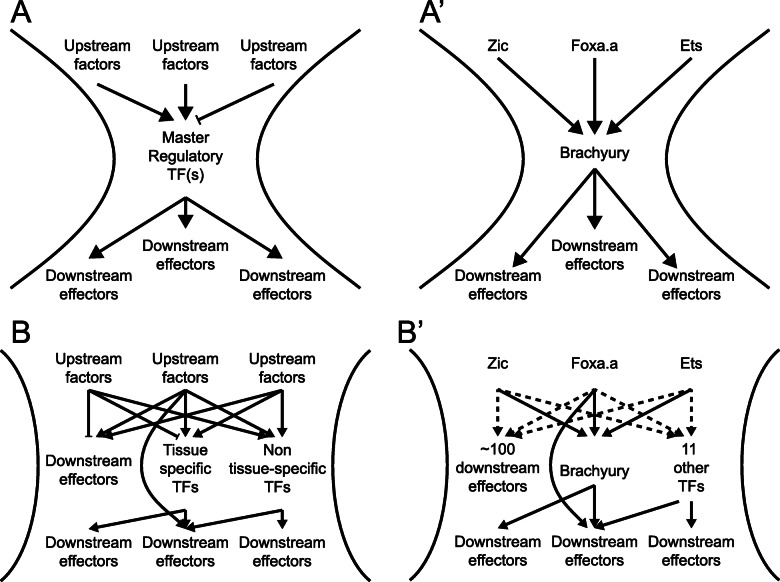
The wide-hourglass model of cell state transitions. **A** A theoretical framework of a “narrow-hourglass” transcriptional response being primarily deployed through induction of a single tissue-specific master regulator. **A’** A narrow-hourglass model of the *Ciona* notochord GRN focused on Brachyury. **B** The wide-hourglass transcriptional response includes many different TFs being upregulated alongside numerous putative effector genes at the time of cell fate induction via the same set of transcriptional inputs. **B’** The wide-hourglass model of the *Ciona* notochord GRN presented in this paper. Dashed lines indicate transcriptional inputs broadly inferred from TFBS enrichment data, but these may not apply to each and every transcript upregulated in the early notochord

The gene regulatory networks controlling cell fate specification events are conventionally thought of as hourglasses with narrow waists (Fig. 8A). Upstream of the cell fate specification event, diverse regulatory interactions give rise to some unique combination of lineage-specific transcription factors and signaling states that induces the expression of either a single master regulatory transcription factor unique to that cell type or a small number of tissue-specific transcription factors that define a combinatorial code. Transcriptional cascades acting downstream of that single factor or small set of factors then lead directly or indirectly to changes in the expression of large numbers of downstream genes. There are major questions, however, about how narrow the pinch point of the GRN hourglass actually is as sibling cell states diverge. A conventional view of *Ciona* primary notochord fate specification would be that the intersection of active Ets, Zic, and Foxa.a expression induces Bra and that Bra then induces early notochord target genes (Fig. 8A’). Here we found that this bifurcation is transcriptomically complex, with many genes, including numerous transcription factors, differentially expressed between sibling cell states within minutes of cell division (Fig. 8B). We propose that the early notochord-specific transcriptional regime is regulated not by Bra but instead by the same combination of active Ets, Zic, and Foxa.a expression that induces Bra and is thus in parallel to and not downstream of Bra activity. In support of this, TFBS enrichment analysis shows that Zic and Ets binding motifs are strongly enriched in the putative regulatory regions of early notochord-enriched genes and it is not until later that Bra motifs become strongly enriched (Fig. 6F and Fig. 8B’).

It is perhaps unsurprising that the combination of active upstream transcription factors that induces a particular cell type would upregulate more than just a few tissue-specific master regulators, but it is surprising that it is not until much later that Bra becomes the predominant TFBS enrichment signature in the fate-restricted notochord. We refer to this as the “broad hourglass” to distinguish it from the “narrow-hourglass” model in which the earliest transcriptional divergence between sibling cell types involves no more than a few key transcriptional regulators of the newly induced cell state. The hourglass metaphor is an apt way to think about the establishment of new transcriptional states, but note that we are not making reference to the concept of the phylotypic stage where an hourglass metaphor has also been widely used [97].

The number of differentially expressed TFs observed across many different cell fate bifurcations raises interesting questions about what fraction of these TFs are functionally important for the bifurcations in which they become differentially expressed. Forward and reverse developmental genetic approaches have generally emphasized the necessity of individual transcription factors for specific cell fate decisions, but that does not exclude more complex sets of TFs playing important roles. Across many different fate bifurcations, we found that the earliest differentially expressed TFs consistently fell into two major classes. Some were stably enriched in the new cell type, whereas others were only transiently enriched. It remains to be determined to what extent transiently enriched TFs represent separable roles in the induction but not maintenance of cell fate as opposed to purely ephemeral responses.

In the bifurcation between primary notochord and A-line neural cell fates, we identified 12 differentially expressed TFs at the 64-cell stage. Some of these, such as Bra and Foxa.a, have well-established roles in notochord fate. Bra was until recently thought to be a master regulator of notochord fate, but it is increasingly clear that Foxa.a has important roles within the fate-restricted notochord lineage and not just as an upstream regulator of Bra expression [84, 86]. Two other notable TFs that are enriched in primary notochord at the 64-cell stage include Elk1/3/4, which appears to be broadly involved in FGF/Ets-mediated cell fate bifurcations, and also Hes-a, which commonly acts as a transcriptional target of Delta/Notch signaling. Delta/Notch signaling is thought to be involved in inducing secondary but not primary notochord fate [45], but the lateral-most primary notochord cells are in contact with Delta2 expressing cells and Hes expression in these cells has been observed by in situ hybridization [45].

### Tissue-specific effector networks

In addition to the early embryonic gene regulatory networks that establish distinct cell types, there are also gene regulatory networks active within distinct cell types that control the temporal aspects of gene expression driving the differentiation and morphogenesis of different tissues. These tissue-specific effector networks have received less attention than the early networks that initially establish cell fates. One major question is whether gene expression in differentiating tissues represents a continuous trajectory versus a discontinuous progression through a series of discrete transcriptional states. Here we integrated our scRNAseq dataset with the Cao et al. dataset to analyze primary notochord gene expression from its origin at the 64-cell stage through to the hatched larvae. We found that the temporal expression patterns of notochord-enriched transcripts clustered into 5 relatively discrete groups, supporting the idea that notochord differentiation involves a progression through several distinct transcriptional states. This concept could be developed further in the future by integrating gene expression profiling, TFBS enrichment, computational inference, and perturbative experiments to build, test, and refine temporally dynamic GRN models of this and other differentiating tissues.

The differentiating notochord undergoes several large-scale morphogenetic events including patterned rounds of asymmetric division, mediolateral intercalation, notochord cell elongation, notochord sheath formation, and notochord lumen formation. For some of these processes, previous studies have provided insight about specific genes involved. One hypothesis is that distinct transcriptional waves in the notochord might control specific aspects of morphogenetic cell behavior. Our preliminary analysis of GO code enrichment in these waves supports this hypothesis. The postmitotic *Ciona* notochord consists of only 40 cells and is thus well suited for testing this and other hypotheses about how developmental GRNs control both cell fate decisions and tissue-specific patterns of morphogenetic cell behavior.

## Materials and methods

### Ciona

Wild caught *Ciona robusta* (formerly *Ciona intestinalis* type A) were purchased from Marine Research and Educational Products (MREP, San Diego). Animals were housed in a recirculating artificial seawater (ASW) aquarium at 18 °C under constant light until experimental use.

### Experimental setup

An independent fertilization, dechorionation, dissociation, encapsulation, and library prep were performed to obtained data for each of the 64-cell, 112-cell, and mid-gastrula stages. The U0126 drug treatments were performed in parallel for each stage using embryos from the same fertilization and dechorionation.

### Fertilization, dechorionation, and embryo culture

Fertilization and dechorionation were performed according to standard protocols [98]. Eggs and sperm were collected from 3 to 6 adults for each fertilization. Washed eggs were inseminated and left to fertilize for 5 min. They were then placed into dechorionation solution and gently pipetted to remove the follicle cells and chorion. Once dechorionation was complete, the eggs were washed 4 times in ASW containing 0.1% BSA. Embryos were incubated at 18 °C, and development was tracked in real time using an in-incubator microscope. Embryos were staged according to [47].

### Drug treatment

Embryos were treated with the MEK inhibitor U0126 (Sigma) from the 16-cell stage onward at a concentration of 4 μM. At the desired stage, a 1000X stock of 4 mM U0126 in DMSO was added to the culture dishes and mixed to give a final concentration of 4 μM. Control embryos were treated with 0.1% DMSO at the same time as drug-treated embryos.

### Dissociation

Embryos were harvested at the desired stage in a 1.5-mL microcentrifuge tube that had been treated with 1% BSA in ASW. Embryos were gently pelleted by centrifugation at 700×*g* for 30 s and the supernatant was removed. They were then washed twice with room temp Calcium-Magnesium Free AWS (CMF-ASW). Dissociation was performed by gentle pipetting of embryos in cold 0.1% BSA/CMF-ASW with 1% trypsin for 2 min. Following dissociation, the embryos were washed twice using cold 0.1% BSA/CMF-ASW, filtered through a 40-μm strainer, and washed once more. Cells were then resuspended in cold 0.1% BSA/CMF-ASW, an aliquot was counted on a Neubauer-Improved hemocytometer, and the remainder were diluted to a concentration of 250,000 cells/mL. A small subsample of embryos from each collection was fixed in 2% paraformaldehyde in ASW to confirm staging and embryo quality by confocal microscopy.

### Encapsulation and RNA capture

Microfluidic encapsulation of single cells with barcoded beads was performed on the Dolomite Nadia instrument (Dolomite Bio, Royston UK) with a 2-lane encapsulation chip. One lane on each chip was used for the DMSO control and one for the U0126-treated sample. We used the standard Nadia protocol (v1.8) and their filter-based emulsion breaking protocol. The capture beads and chemistry used in this protocol are essentially the same as in [3] and use a stringent lysis buffer and reverse transcription after emulsion breaking. The beads are coated with oligos that incorporate a poly(T) sequence to capture mRNA 3′ ends and also two barcode sequences. One is a cell barcode that is shared between every oligo on a given bead. The other is a short Unique Molecular Identifier (UMI) barcode that differs between oligos on each bead and is used to correct for PCR duplicates in each sequenced library.

### Library preparation and sequencing

After RNA capture and emulsion breaking, reverse transcription, exonuclease treatment, and PCR were performed according to the Dolomite protocols. Sequencing libraries were generated using the Illumina Nextera XT kit and library quality was assessed using an Agilent Bioanalyzer. Sequencing was performed on an Illumina NextSeq 500 at the KSU Integrated Genomics Facility. For all sequencing runs, an Illumina High-output 150 cycle kit was used with read lengths of 26 bp for the Cell barcode/UMI read, 8 bp for the i7 index read, and 116 bp for the transcript read. For each timepoint, the DMSO and U0126 libraries were sequenced in a pool across all four lanes of an Illumina chip.

### Sequence alignment and demultiplexing

Following sequencing, the dropSeqPipe pipeline [3] was used to align reads to the genome, count individual UMIs, and assign them to STAMPs. Default parameters were used except the following: Genome build—KH2012 (accessible via ANISEED database), 5 prime SMART adapter sequence—AAGCAGTGGTATCAACGCAGAGTGAATGGG, cell barcode start position—1, cell barcode end position—12, UMI barcode start position—13, UMI barcode end position—21, STAR outFilterMismatchNmax—15, STAR outFilterMismatchNoverLmax—0.3

### Maternal demultiplexing via SNPs

The GATK best-practices pipeline for variant calling in single-cell RNAseq data was used to call SNPs across the entire genome individually for all STAMPs [99]. The VCFtools package was used to calculate a relatedness statistic (unadjusted Ajk statistic based on [52]) between all pairwise STAMP comparisons in controls and drug-treated embryos at each stage [100]. The resulting distance matrix was clustered using the cluster module of the python Scipy package [101]. A distance cutoff for clustering was selected created the number of clusters mirroring the number of adults used for each experiment after examining dendrograms created from the distance matrix. Parameters used in this pipeline can be accessed via github (https://github.com/chordmorph/ciona_scRNAseq). Only two maternal clusters were seen in the mid-gastrula timepoint despite three adults having been used. This is likely because the eggs from one adult either did not fertilize or else developed poorly and were removed prior to dissociation.

### Dimensional reduction, clustering, and cluster analysis

Dimensionality reduction, clustering, and further downstream analysis were performed using Seurat.v4 [53, 54, 102, 103]. STAMPs with low numbers of genes detected (64-cell: < 1750, 112-cell: < 2500, mid-gastrula: < 1500) were filtered out to remove likely empty droplets or low-quality transcriptomes. These thresholds were selected ad hoc after observing the distribution of gene counts in the datasets. STAMPs that were not contained in one of the well-defined “mother-of-origin” SNP clusters at each stage were also filtered out to remove potential doublets or low-quality transcriptomes. For all timepoints, the SCTransform functionality in Seurat [102] was used for gene count normalization and scaling using mother-of-origin as a batch variable. Linear dimensional reduction was performed using principle component analysis (PCA), and the number of PCs to keep for downstream clustering and non-linear dimensional reduction was determined by the modified Jackstraw Procedure. The number of PCs used for all downstream analysis (non-linear dimensional reduction/UMAP, clustering, pairwise cluster distance measurement) are as follows: DMSO 64-cell—30, U0126 64-cell—30, DMSO and U0126 64-cell—30, DMSO 112-cell—40, U0126 112-cell—30, DMSO and U0126 112-cell—35, DMSO mid-gastrula—40, U0126 mid-gastrula—30, DMSO and U0126 mid-gastrula—30, DMSO and U0126 all stages—25. Distances between clusters were determined using the Euclidian distance between cluster centroids in a PCA reduced gene expression space that contained DMSO- and U0126-treated cells at all stages.

### Cell type and lineage assignment

Cell types were assigned to DMSO-treated cells at each stage by identifying marker genes and validating their expression domains using the ANISEED database [48]. These cell type labels were then transferred from DMSO-treated cells to U0126-treated cells at each stage independently using the FindTransferAnchors and TransferData functions in Seurat. Lineages were assigned using the known and stereotyped lineages in the *Ciona*. Occasionally, a single cluster at one stage gave rise to more than two clusters at the next stage. When this occurred, lineages were assigned according to the known lineage relationships. These “quadfurcations” were excluded from analysis of quasi-mother/daughter relationships but the inferred bifurcations contained in each quad group were analyzed for TFBS enrichment.

### Novel cell type validation

In situ hybridization probes were generated using template sequences synthesized by Twist Biosciences as linear fragments. These ~ 500 base pair synthetic constructs included a T7 promoter at the 3′ ends and were flanked by the standard Twist adapter sequences. The probe constructs were amplified using primers specific to the Twist adapters and then used as templates for DIG-labeled probe synthesis. In situ hybridization details were otherwise as previously described [89]. All of the sequences were designed to have minimal homology to other *Ciona* transcripts. KH genome assembly coordinates for the probe sequences used are:

C2.629 (Fam167) KhC2: 5033045-5033380 + KhC2: 5032521-5032686

C6.129 (neurogenin) KhC6:1504284-1504783

C9.19 (RGS) KHC9:2188968-2189172 and 2189651-2189814 and 2189239-2189306 and 2190109-2190173

C2.957 (MsxB) KhC2:5774333-5774655 and 5773769-5773945.

The genome coordinates are discontinuous for some probes due to introns.

The neurogenin (b-line)>H2BVenus construct used is from [58]. We electroporated it into fertilized eggs using 40 μg of plasmid in 800 μL electroporations by standard methods. Embryos fixed in 2% paraformaldehyde in seawater were immunostained for the Venus reporter using a polyclonal anti-GFP primary antibody (Invitrogen) and Alexa555 anti-rabbit secondary antibody (Invitrogen). They were also counterstained with phalloidin (Invitrogen) to label cell cortices. Embryos were cleared in Murray’s clear (benzyl alcohol/benzyl benzoate) and imaged on a Zeiss 880 LSCM using a 40 × 1.3NA oil immersion objective. We did not detect reporter expression in posterior b-line cells at stage 12 but could by stage 12.5. This likely reflects the time needed for the reporter to be translated to detectable levels.

### TFBS analysis

TFBS analysis was performed similar to [86]. Control and target sequence sites were determined using the FindMarkers function implemented in Seurat with default parameters to find statistically significantly up- and downregulated genes with a Log2 fold-change cutoff of 0.3. To extract putative enhancer regions from these gene models, we extracted the 1500 bp of sequence upstream of the transcription start site. Additionally, we intersected these upstream regions with whole-embryo ATACseq peaks from previous experiments and kept the entire sequence of any peak that was overlapping the 1500 bp upstream for a gene model. These sets of sequences were then saved as fasta files and used as the input for TFBS site calling and enrichment testing using the oPOSSUM3 [67] command line-tools with default settings and the JASPAR2020 vertebrate core PWMs [104]. The oPOSSUM *z*-score obtained from the full 1500 bp upstream region and from the ATAC regions overlapping the 1500 bp upstream of the TSS for each PWM were averaged to create a single *z*-score that incorporated information from both sources of data.

### Differential expression testing

Differential expression testing was performed with the FindMarkers function implemented in Seurat with default parameters using the Wilcoxon rank-sum test statistic. For differential expression between sibling clusters, testing was performed between only the two clusters. For identification of “enriched” genes in the notochord, testing was performed using the notochord cells versus all other cells.

### Average expression measurement

Pseudo-bulk expression measurements for each cell type at each stage were generated using the AverageExpression function in Seurat. Pseudo-bulk counts were made for all cell types in a common gene expression space after normalization and scaling with SCTransform.

### Elk1/3/4 ATACseq analysis

Called peaks from Madgwick et al.’s [63] early whole-embryo ATACseq experiments were downloaded from ANISEED and putative TFBSs were identified using the FIMO TFBS scanner with a *p* value threshold of 0.001 and the JASPAR2020 vertebrate core PWMs. Bedtools was used to associate each peak with the closest gene model in the KH2012 gene build. Sites matching the human ETS1, ELK1, ERG, and ETV matrices were de-duplicated by taking the best match within a 3-bp window of the center of each hit.

### FGF sensitivity scoring

Each cell type bifurcation was tested for sensitivity to U0126 treatment by performing a chi-square test for independence between the ratio of cells resulting from a bifurcation and the presence of U0126. This was done by summing the number of cells present for all descendants at mid-gastrula of each cell type in a bifurcation in both DMSO and U0126 treatment conditions, the resulting 2 × 2 matrix (descendant cell number by treatment) was used as the input for the chisq.test function implemented in the base R package. In the case that a bifurcation was significantly affected by U0126 treatment, the downstream bifurcations of the eliminated cell type were excluded from further testing as both cell types were often completely absent from the U0126-treated cells. For visualization in Fig. 5F, we used the negative log of the *p* values for display purposes to better show differences between the FGF-sensitive bifurcations.

### Elk1 as a mediator of ETS signaling

Elk1/3/4 was identified as having a unique pattern of expression among TFs in FGF-sensitive cell types by hierarchical clustering the Log_2_ fold-change of all TFs that were significantly differentially expressed in at least one FGF-sensitive cell type.

The relationship between Elk1 and Ets signaling was further explored by calculating the Ets TFBS enrichment score of the genes that are differentially expressed between sibling cell types; the Ets TFBS enrichment score of the genes that are differentially expressed in the first sibling cell type compared to all other cell types at a given stage; the Ets TFBS enrichment score of the genes that are differentially expressed in the second sibling cell type compared to all other cell types at a given stage; and the difference of log-scaled expression value in one sibling cell type compared to the other sibling cell type for all bifurcations. For these comparisons, we considered the known FGF-sensitive sibling or the sibling with greater Ets site usage in the sib-sib comparison to be the “first” cell type and its sibling state to be the “second” cell type. These 4 variables were then used for hierarchical clustering to identify different patterns with respect to their Elk1 expression and Ets TFBS enrichment characteristics.

### Trio differential transcription factor expression analysis

Trios were defined when a pair of sibling cell types at a given stage as well as their direct parent cell type at the previous stage were all identifiable. Differentially expressed transcription factors between the sibling cell types were identified using the FindMarkers function in Seurat and all significant (*p* value < 0.05) TFs were kept for further analysis. The average log_2_ Fold-change was calculated between each sibling cell type and maternal cell type. A TF was considered upregulated in a daughter cell type compared to the mother cell type if the average log_2_ fold-change was greater than 0.3475 and was considered downregulated vs the mother cell type if the average log_2_ fold-change was less than − 0.3475. TFs with average log_2_ fold-change between − 0.3475 and 0.3475 were considered as “not changed” vs the expression level in the mother cell type.

### Expression waves in the notochord

The union of the genes that were differentially expressed in the notochord vs all other cell types at each stage were used to define the notochord-enriched gene set list for further analysis. Average expression of these genes throughout development were obtained using the AverageExpression functionality in Seurat. Pseudo-bulk expression levels of the notochord-enriched gene set list were compared across all stages analyzed and were clustered using Ward’s variance minimization method. The resulting clusters were isolated using a manually selected distance cutoff that clearly defined patterns of distinct gene expression modules.

## Supplementary Information

**Additional File 1: Figure S1**. Cell lineages and division patterns are stereotyped during early *Ciona* development. This figure summarizes the stereotyped cell lineages of early ascidian embryos based on the work of ([20–23, 43, 109, 110]). A-D’) Animal and vegetal views of the *Ciona* embryo at the 16-cell (A-A’), 32-cell (B-B’), 64-cell (C-C’), and 112-cell (D-D’) stages. On each view, the blastomere name is labeled on the right half of the embryo, and the cell type is colored on the left half of the embryo for lineage restricted blastomeres. E-E’) Fate map of the animal blastomeres at the 112-cell stage (E), and their future territory at the mid-tailbud stages (E’). Colors represent sibling cell pairs and do not match the color scheme used in A-C. Adapted from [21]. F) Cartoon representation of the neural plate at mid-gastrula stage. Blastomere names are printed on the right half of the neural plate. Arabic numerals define the column numbers, while roman numerals define the row numbers. Colors on the left half of the neural plate define the cellular lineage and correspond with those in A-D’ and G (magenta: A-line, orange: a-line, brown: b-line). G) Lineage tree of blastomeres in the early stages of *Ciona* development. Colors correspond with A-D’ and F.

**Additional File 2: Table S1** - Sequencing statistics. **Table S2** - Number of stamps in each cluster for all treatments and developmental stages. **Table S3** - Marker genes for all clusters at the 112-cell stage. **Table S4** - Transcription factor differential expression in “trios”. **Table S5** - GO code enrichment for Biological Process terms in notochord waves. **Table S6** - Transcription factor association with open chromatin length in *Ciona.* **Table S7** - Effector TFs associated with putative *Ciona* super-enhancers.

**Additional File 3: Figure S2**. Adult of origin effect correction using SNPs. A-C) First-pass UMAP plots at the 64-cell (A), 112-cell (B), and mid-gastrula (C) stages where the animal lineages are colored in red. D-F) Dendrograms of heterarchical clustering of genetic relatedness of STAMPs in the 64-cell (D), 112-cell (E), and mid-gastrula (F) stages using SNPs. G-I) UMAP plots at the 64-cell (G), 112-cell (H), and mid-gastrula (I) stages where STAMPs have been colored by the putative adult-of-origin using the clustering in D-F. UMAP space is identical to A-C.

**Additional File 4: Figure S3**. Marker genes of all cell types at the 64-cell stage. All cells are shown clustered into their assigned cell types. The top 10 marker genes as ranked by Log2 fold-change are shown for each cell type unless used as a marker of a previously clustered cell type. Color scale represents depth-corrected log-scaled expression value in each cell.

**Additional File 5: Figure S4**. Marker genes of all cell types at the mid-gastrula stage. All cells are shown clustered into their assigned cell types. The top 10 marker genes as ranked by Log2 fold-change are shown for each cell type unless used as a maker of an earlier cell type. Color scale represents depth-corrected log-scaled expression value in each cell.

**Additional File 6: Figure S5**. Differences in transcriptional states vary by stage after U0126 treatment. A-B) UMAP projection of all DMSO and U0126 treated STAMPS at 64 cell stage (A) and 112 cell stage (B) colored by treatment. Dotted lines divide STAMPS into their developmental lineages.

**Additional File 7: Figure S6**. Poster image showing differential transcription factor expression and TFBS enrichment for all bifurcations.

**Additional File 8: Figure S7**. The secondary notochord also exhibits transcriptional waves. A-B) Temporal expression profiles of all secondary notochord enriched genes (A), and transcription factors (B) reveal distinct waves of expression throughout the course of development similar to the primary notochord.

**Additional File 9: Figure S8**. TFBS enrichment in the primary notochord. Left panel) Average TFBS motif enrichment z-score in the primary and secondary notochord from the 64-cell, 112-cell, and mid-gastrula stages, which represent the early notochord GRN. Right panel) Average TFBS motif enrichment z-score in the primary and secondary notochord from the final three stages of development assayed, which represent the late notochord GRN.

## Data Availability

Raw sequencing reads, along with unprocessed (dropSeqPipe output) and processed Seurat objects for each stage are available online at the Gene Expression Omnibus (GEO accession number: GSE160701) [105]. The single-cell data can also be browsed on the UCSC Single Cell Viewer (https://ciona-dev.cells.ucsc.edu) [106, 107]. Code used for SNP calling and clustering and TFBS analysis are available on the Veeman lab Github page (https://github.com/chordmorph/ciona_scRNAseq) [108].
