# Supplementary figures and images for: Single-cell analysis of cell fate bifurcation in the chordate Ciona
Source: BMC Biol. 2021 Aug 31;19:180. doi: 10.1186/s12915-021-01122-0 (PMC8408944; doi:10.1186/s12915-021-01122-0)

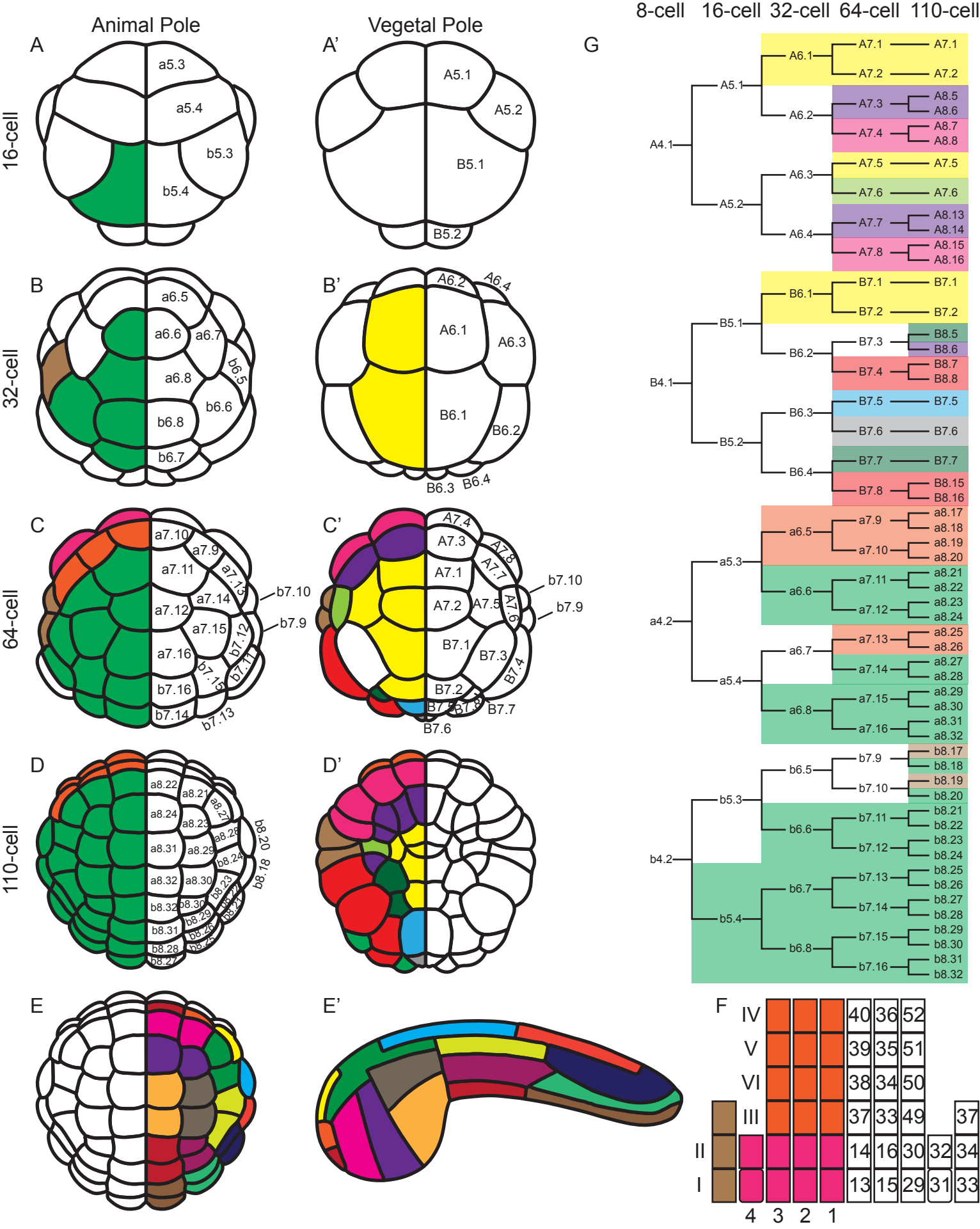

Supplement: Supplementary file 1 — Additional File 1: Figure S1. Cell lineages and division patterns are stereotyped during early Ciona development. This figure summarizes the stereotyped cell lineages of early ascidian embryos based on the work of ([20–23, 43, 109, 110]). A-D’) Animal and vegetal views of the Ciona embryo at the 16-cell (A-A’), 32-cell (B-B’), 64-cell (C-C’), and 112-cell (D-D’) stages. On each view, the blastomere name is labeled on the right half of the embryo, and the cell type is colored on the left half of the embryo for lineage restricted blastomeres. E-E’) Fate map of the animal blastomeres at the 112-cell stage (E), and their future territory at the mid-tailbud stages (E’). Colors represent sibling cell pairs and do not match the color scheme used in A-C. Adapted from [21]. F) Cartoon representation of the neural plate at mid-gastrula stage. Blastomere names are printed on the right half of the neural plate. Arabic numerals define the column numbers, while roman numerals define the row numbers. Colors on the left half of the neural plate define the cellular lineage and correspond with those in A-D’ and G (magenta: A-line, orange: a-line, brown: b-line). G) Lineage tree of blastomeres in the early stages of Ciona development. Colors correspond with A-D’ and F. [file 12915_2021_1122_MOESM1_ESM.pdf]

**A**

UMAP 2

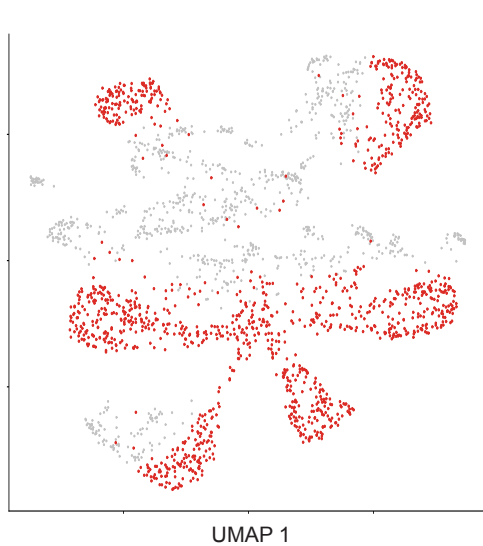**B**

UMAP 2

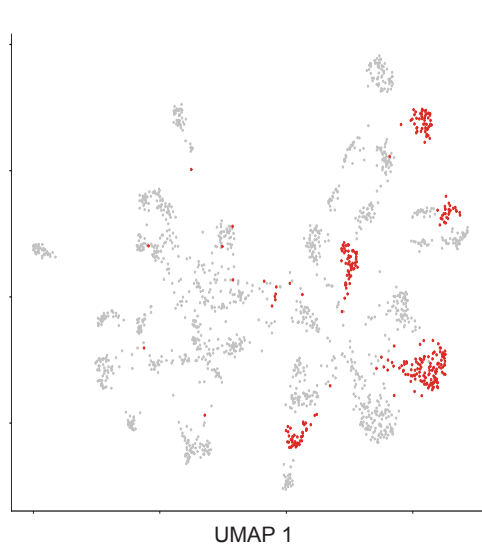**C**

UMAP 2

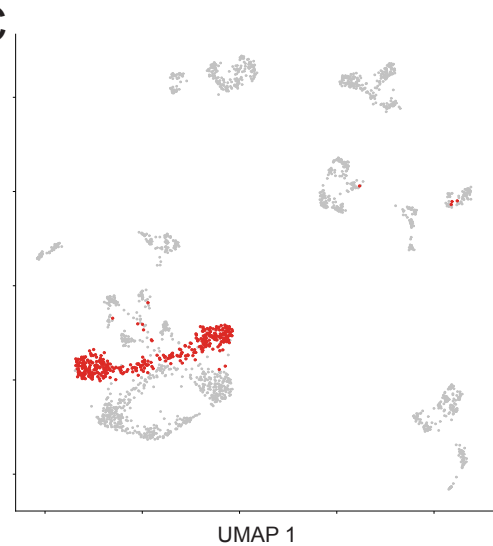**D**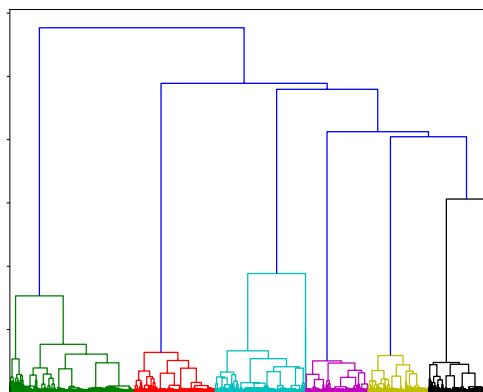**E**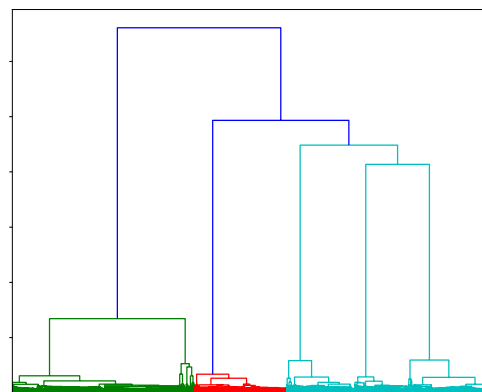**F**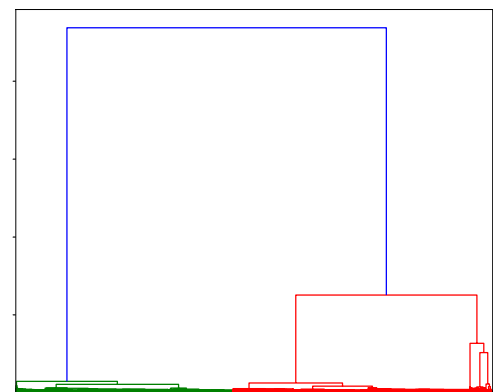**G**

UMAP 2

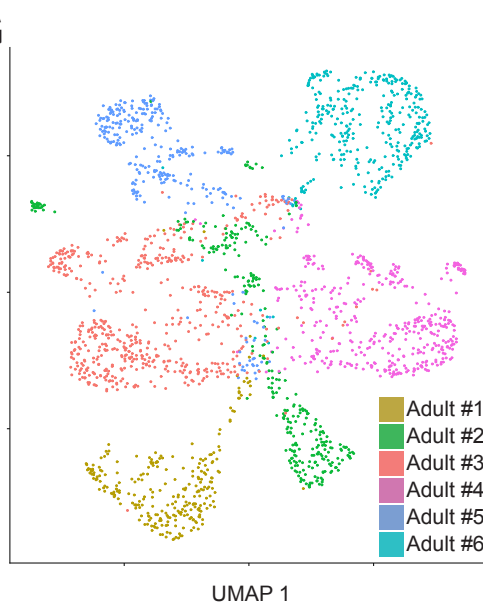**H**

UMAP 2

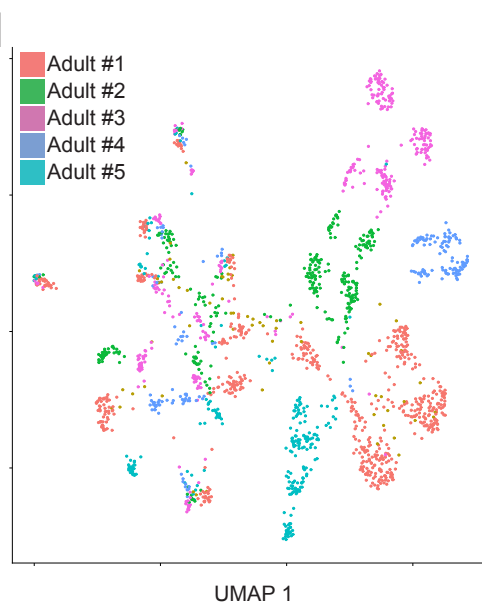**I**

UMAP 2

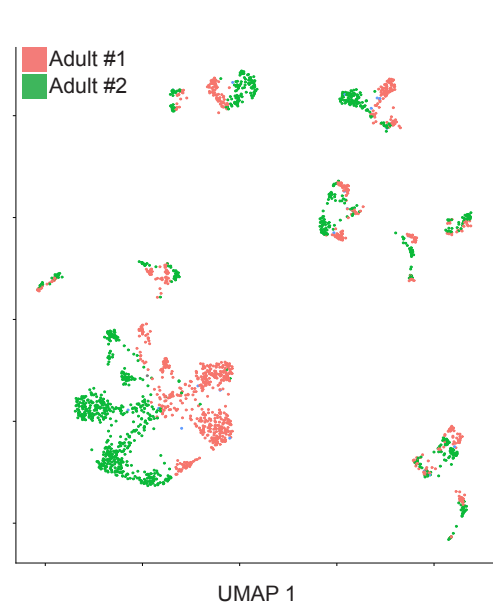

Supplement: Supplementary file 3 — Additional File 3: Figure S2. Adult of origin effect correction using SNPs. A-C) First-pass UMAP plots at the 64-cell (A), 112-cell (B), and mid-gastrula (C) stages where the animal lineages are colored in red. D-F) Dendrograms of heterarchical clustering of genetic relatedness of STAMPs in the 64-cell (D), 112-cell (E), and mid-gastrula (F) stages using SNPs. G-I) UMAP plots at the 64-cell (G), 112-cell (H), and mid-gastrula (I) stages where STAMPs have been colored by the putative adult-of-origin using the clustering in D-F. UMAP space is identical to A-C. [file 12915_2021_1122_MOESM3_ESM.pdf]

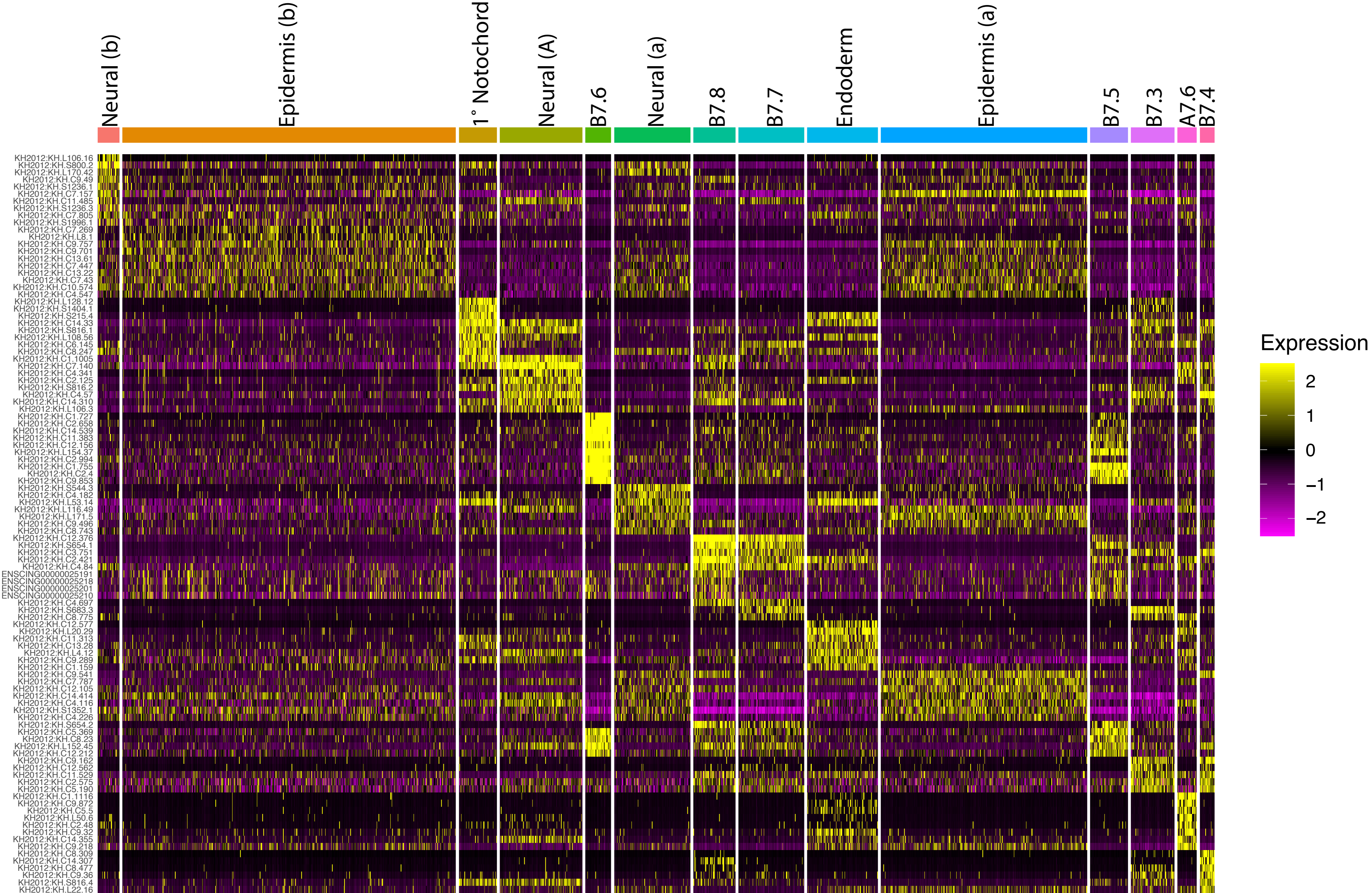

Supplement: Supplementary file 4 — Additional File 4: Figure S3. Marker genes of all cell types at the 64-cell stage. All cells are shown clustered into their assigned cell types. The top 10 marker genes as ranked by Log2 fold-change are shown for each cell type unless used as a marker of a previously clustered cell type. Color scale represents depth-corrected log-scaled expression value in each cell. [file 12915_2021_1122_MOESM4_ESM.pdf]

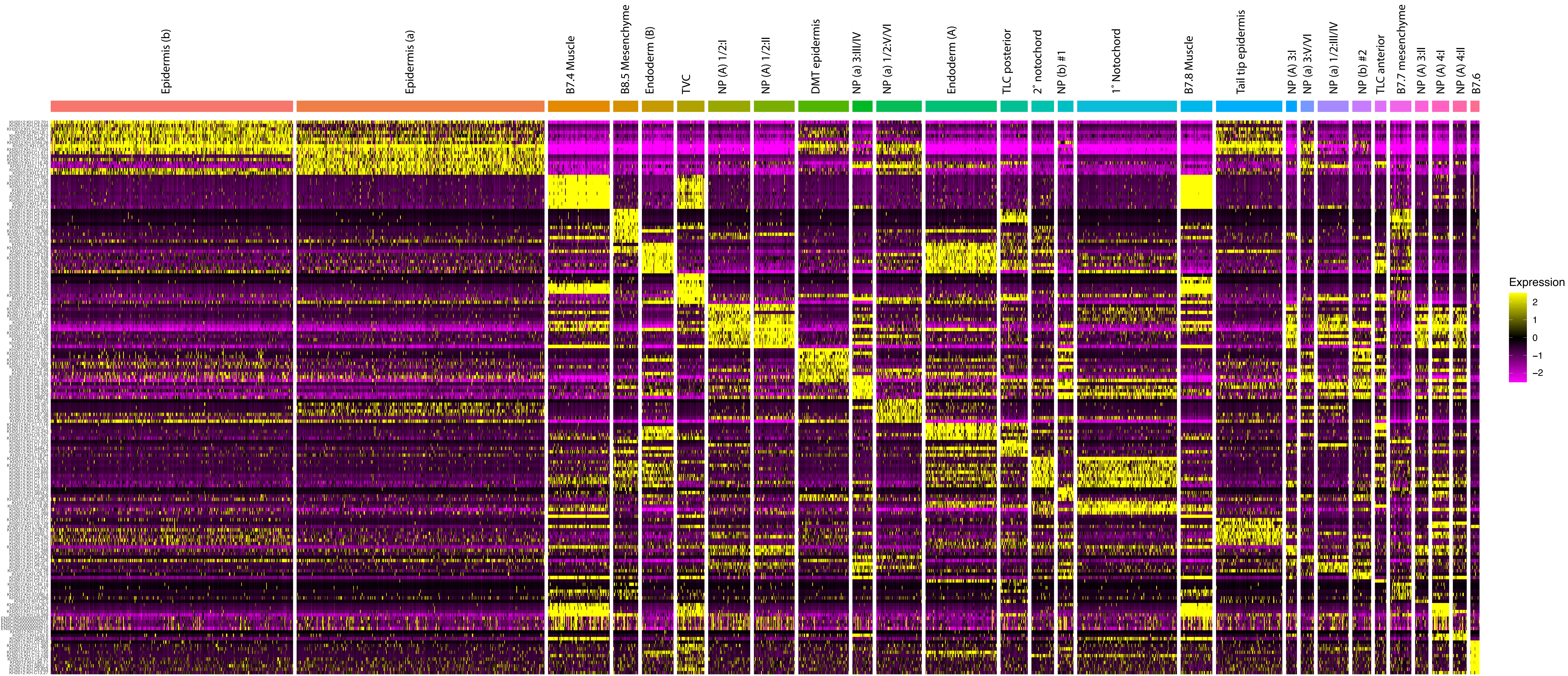

Supplement: Supplementary file 5 — Additional File 5: Figure S4. Marker genes of all cell types at the mid-gastrula stage. All cells are shown clustered into their assigned cell types. The top 10 marker genes as ranked by Log2 fold-change are shown for each cell type unless used as a maker of an earlier cell type. Color scale represents depth-corrected log-scaled expression value in each cell. [file 12915_2021_1122_MOESM5_ESM.pdf]

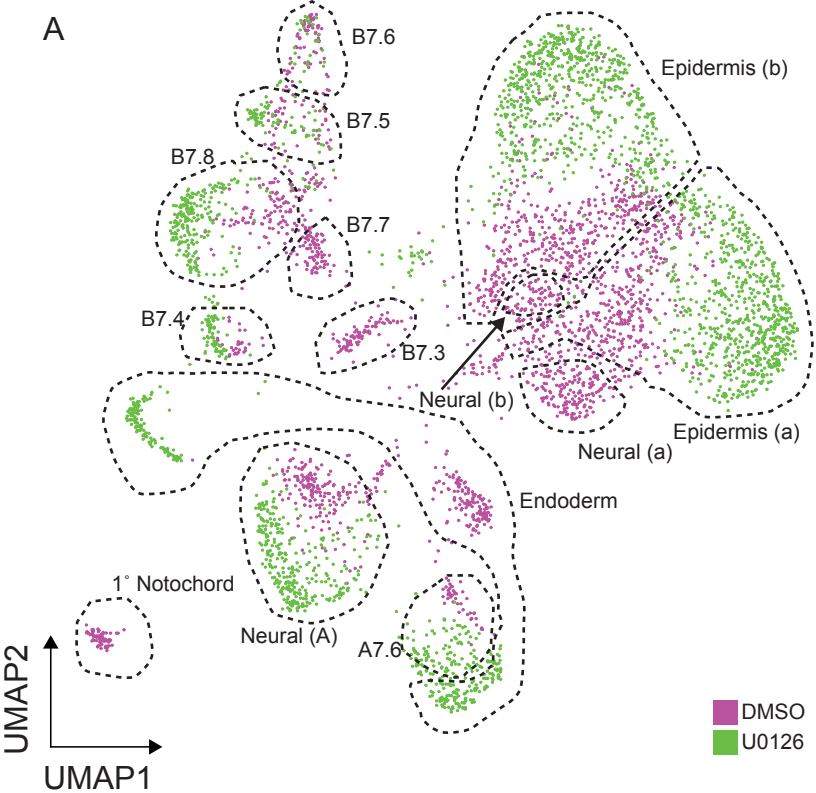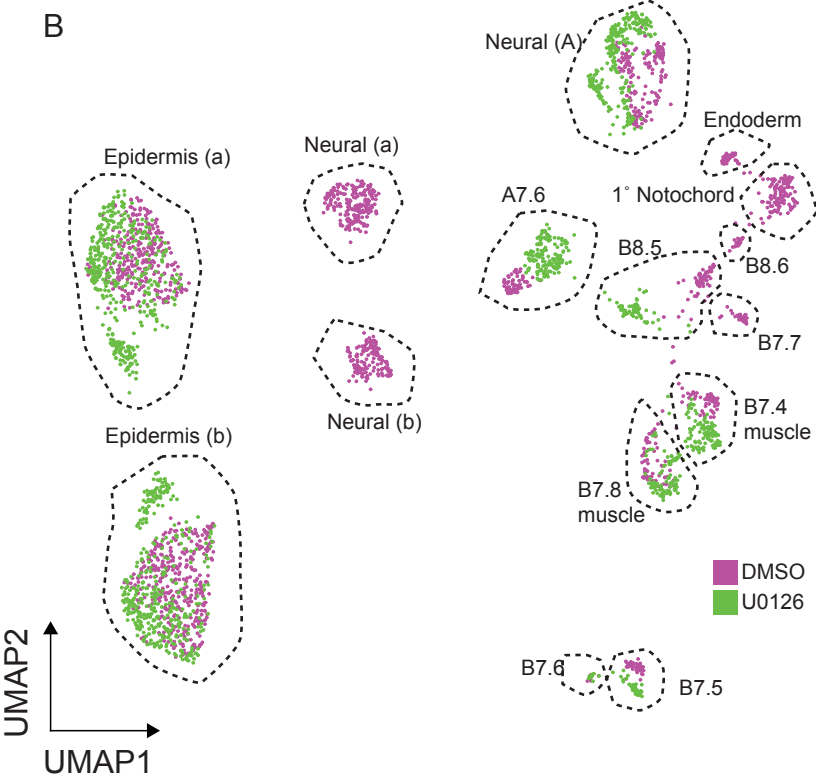

Supplement: Supplementary file 6 — Additional File 6: Figure S5. Differences in transcriptional states vary by stage after U0126 treatment. A-B) UMAP projection of all DMSO and U0126 treated STAMPS at 64 cell stage (A) and 112 cell stage (B) colored by treatment. Dotted lines divide STAMPS into their developmental lineages. [file 12915_2021_1122_MOESM6_ESM.pdf]

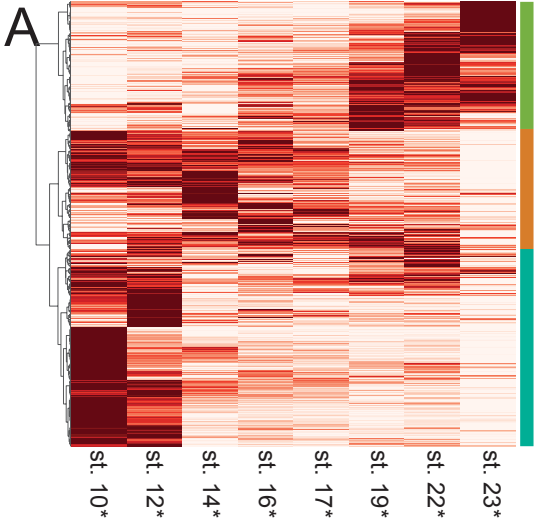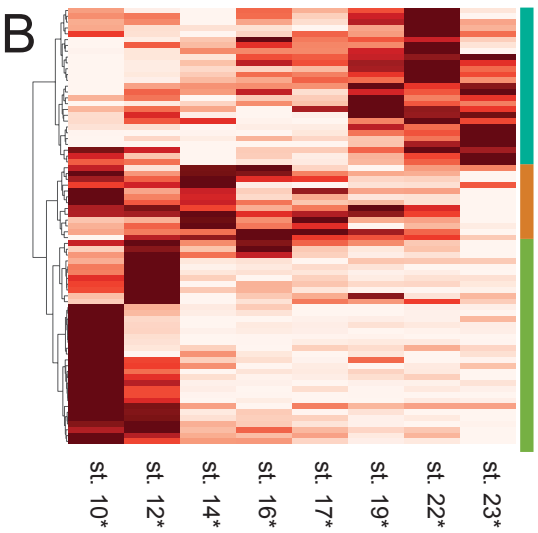

Max mean  
reads/cell

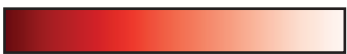

Min mean  
reads/cell

Supplement: Supplementary file 8 — Additional File 8: Figure S7. The secondary notochord also exhibits transcriptional waves. A-B) Temporal expression profiles of all secondary notochord enriched genes (A), and transcription factors (B) reveal distinct waves of expression throughout the course of development similar to the primary notochord. [file 12915_2021_1122_MOESM8_ESM.pdf]

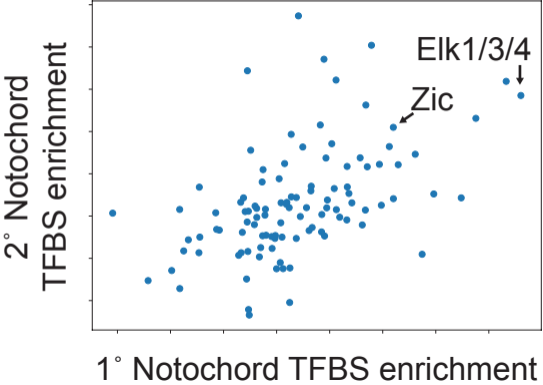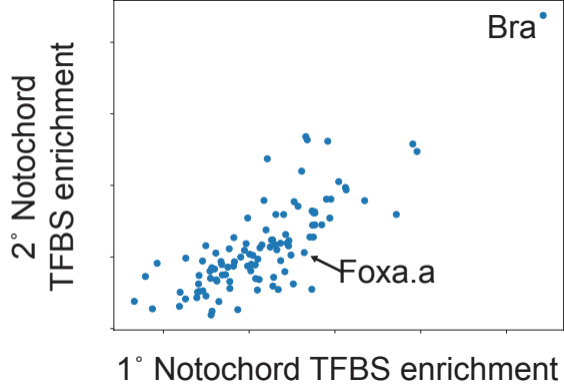

Supplement: Supplementary file 9 — Additional File 9: Figure S8. TFBS enrichment in the primary notochord. Left panel) Average TFBS motif enrichment z-score in the primary and secondary notochord from the 64-cell, 112-cell, and mid-gastrula stages, which represent the early notochord GRN. Right panel) Average TFBS motif enrichment z-score in the primary and secondary notochord from the final three stages of development assayed, which represent the late notochord GRN. [file 12915_2021_1122_MOESM9_ESM.pdf]
