# Supplementary material for: Single-cell analysis of cell fate bifurcation in the chordate Ciona
Source: BMC Biol. 2021 Aug 31;19:180. doi: 10.1186/s12915-021-01122-0 (PMC8408944; doi:10.1186/s12915-021-01122-0)
Supplement: Supplementary file 7 — Additional File 7: Figure S6. Poster image showing differential transcription factor expression and TFBS enrichment for all bifurcations. [file 12915_2021_1122_MOESM7_ESM.pdf]

Epidermis (a)

KH2012 gene model	TF common name	Log2FC Sibling Cluster	-Log(pvalue) Sibling Cluster	DE in Imai	Matched PWM z-score	PWM Cluster z-score
KH2012:KH.C7.157	Emc	1.0858	68.2257	Yes	No PWM match	No PWM match
KH2012:KH.L39.1	FoxH-b	0.4047	14.2280	No	-3.024	0.0940000000000001
KH2012:KH.S544.3	DMRT1	-0.6776	-47.6519	No	1.158	5.344
KH2012:KH.C4.182	Zinc Finger (C3H)	-0.7090	-54.6601	No	No PWM match	No PWM match
KH2012:KH.C8.247	ELK	-0.7300	-42.0388	No	-3.898	-2.0305
KH2012:KH.C4.84	Otx	-0.8333	-72.7302	No	-0.953	0.0940000000000001

JASPAR 2020 PWM cluster	Max Z-score in cluster	Max Z-score in cluster
Rarg	75.60	NR1D1
TFAP2B	30.65	Znf423

Epidermis (a)

KH2012 gene model	TF common name	Log2FC Sibling Cluster	-Log(pvalue) Sibling Cluster	Log2FC Parent Cluster	-Log(pvalue) Parent Cluster	DE in Imai	Matched PWM z-score	PWM Cluster z-score
KH2012:KH.C11.485	Irx-B	1.2003	116.9921	1.0802	158.9679	Yes	No PWM Hits	No PWM Hits
KH2012:KH.L39.1	FoxH-b	0.7741	47.6850	1.0678	107.9231	No	-1.2295	3.2135
KH2012:KH.C1.159	E(spl)/hairy-a	0.6779	38.4790	0.5317	32.6036	Yes	No PWM Hits	No PWM Hits
KH2012:KH.C7.43	AP-2-like2	0.6172	37.6185	0.5806	46.7604	Yes	No PWM Hits	No PWM Hits
KH2012:KH.C1.99	SoxB1	0.5548	30.5887	0.3838	22.9943	No	No PWM match	No PWM match
KH2012:KH.C7.157	Emc	0.5105	23.1007	0.7727	72.3267	Yes	No PWM match	No PWM match
KH2012:KH.L4.17	Zinc Finger (C2H2)-18	0.4052	15.3898	0.4899	27.9922	No	4.424	4.424
KH2012:KH.C9.580	RAR	0.3622	15.3520	0.6032	53.5406	No	0.266	5.3025
KH2012:KH.C13.22	Zinc Finger (C2H2)-2	0.3572	11.7739	0.1995	5.2021	No	-0.837	25.481
KH2012:KH.C4.366	ERF2	-0.5676	-33.3507	-0.1284	-1.8683	No	-12.7385	13.3995
KH2012:KH.C7.523	SoxC	-0.6696	-52.2414	-0.0241	-0.3332	No	No PWM match	No PWM match
KH2012:KH.S816.1	ZicL	-0.9639	-58.6941	0.3218	28.8319	No	-2.3715	-0.869
KH2012:KH.C4.84	Otx	-1.2276	-117.1020	-0.1716	-3.8110	No	2.0395	3.2135
KH2012:KH.C4.182	Zinc Finger (C3H)	-1.3404	-112.7477	0.4777	46.5566	No	No PWM match	No PWM match
KH2012:KH.S544.3	DMRT1	-1.3524	-146.7600	-0.0584	-0.4200	No	4.657	11.041
KH2012:KH.C8.247	ELK	-2.3142	-181.1927	-0.0194	-0.1980	No	-15.6235	13.3995

JASPAR 2020 PWM cluster	Max Z-score in cluster	Max Z-score in cluster
ZNF143	34.71	ZNF143
SP3	25.48	EGR4
PROX1	18.75	PROX1
NFIL3	18.64	NFIL3
Nr2f6	17.53	PPARA::RXRA
POU3F1	17.27	POU2F2
TFAP2B	16.69	Znf423
HOXD10	15.52	HOXD11

Anterior Epidermis

KH2012 gene model	TF common name	Log2FC Sibling Cluster	-Log(pvalue) Sibling Cluster	Log2FC Parent Cluster	-Log(pvalue) Parent Cluster	DE in Imai	Matched PWM z-score	PWM Cluster z-score
KH2012:KH.C1.159	E(spl)/hairy-a	1.1649	67.2323	0.4307	13.0532	Not Analyzed	No PWM Hits	No PWM Hits
KH2012:KH.C1.99	SoxB1	1.0618	80.9041	0.0930	1.6778	Not Analyzed	No PWM match	No PWM match
KH2012:KH.C11.485	Irx-B	1.0589	75.8930	-0.1567	-7.7342	Not Analyzed	No PWM Hits	No PWM Hits
KH2012:KH.C1.1016	GCNF	0.8037	40.9542	1.1058	93.7105	Not Analyzed	No PWM match	No PWM match
KH2012:KH.L4.17	Zinc Finger (C2H2)-18	0.5982	39.1374	0.2139	9.3270	Not Analyzed	2.232	2.232
KH2012:KH.L108.4	CREB/ATF-a	0.5718	38.4389	0.2287	10.0622	Not Analyzed	5.698	8.7635
KH2012:KH.C7.43	AP-2-like2	0.5562	24.6653	-0.0574	-1.6238	Not Analyzed	No PWM Hits	No PWM Hits
KH2012:KH.C9.580	RAR	0.5294	28.0260	0.8181	80.2168	Not Analyzed	-1.67	2.512
KH2012:KH.S164.12	SoxB2	0.4523	21.7556	0.4765	34.1611	Not Analyzed	7.4585	12.9095
KH2012:KH.L40.18	Zinc Finger (C2H2)-6	0.3673	21.0599	0.3624	30.9958	Not Analyzed	-3.1435	-3.1435
KH2012:KH.L13.24	FoxN2/3	0.3408	19.4835	0.7825	95.1463	Not Analyzed	-1.2005	10.899
KH2012:KH.S696.1	GATA-b	0.3255	23.1858	0.2164	12.9716	Not Analyzed	No PWM Hits	No PWM Hits
KH2012:KH.C2.573	Smad1/5	0.2988	12.1254	0.2378	10.7921	Not Analyzed	No PWM Hits	No PWM Hits
KH2012:KH.C7.269	Orphan bHLH1	0.2690	4.7083	0.5549	25.6785	Not Analyzed	No PWM match	No PWM match
KH2012:KH.C9.561	Zinc Finger (C2H2)-9	0.2614	10.3973	0.3130	19.8318	Not Analyzed	4.827	4.827
KH2012:KH.C3.230	Smad6/7	-0.2723	-11.4650	0.0996	1.6316	Not Analyzed	No PWM match	No PWM match
KH2012:KH.C7.157	Emc	-0.2895	-3.0995	-0.1041	-7.9538	Not Analyzed	No PWM match	No PWM match
KH2012:KH.C6.71	Tcf/Lef	-0.3029	-13.5060	-0.4768	-40.4539	Not Analyzed	No PWM match	No PWM match
KH2012:KH.C10.106	MITF	-0.3275	-23.8154	-0.2328	-25.6709	Not Analyzed	No PWM Hits	No PWM Hits
KH2012:KH.C3.348	E12/E47	-0.3362	-12.8691	0.2006	8.4071	Not Analyzed	No PWM Hits	No PWM Hits
KH2012:KH.C3.17	ROR	-0.3840	-13.3981	0.1290	1.4613	Not Analyzed	-0.7675	5.065
KH2012:KH.C10.113	ets/pointed2	-0.5190	-22.8565	-0.3047	-19.0784	Not Analyzed	-14.2875	1.7435
KH2012:KH.C4.182	Zinc Finger (C3H)	-0.5305	-30.4451	0.3941	8.0603	Not Analyzed	No PWM match	No PWM match
KH2012:KH.L57.25	FoxC	-0.7184	-65.5264	0.0057	0.2003	Not Analyzed	6.36	10.899
KH2012:KH.S544.3	DMRT1	-0.9872	-131.7959	-0.1879	-21.4990	Not Analyzed	4.3355	10.007
KH2012:KH.C3.751	Snail	-0.9987	-40.6876	-0.0081	-1.3998	Not Analyzed	No PWM Hits	No PWM Hits
KH2012:KH.C4.366	ERF2	-1.0059	-73.8735	-0.0886	-6.3107	Not Analyzed	-8.2665	1.7435
KH2012:KH.C7.523	SoxC	-1.2005	-116.5671	-0.1514	-11.1984	Not Analyzed	No PWM match	No PWM match
KH2012:KH.S816.1	ZicL	-1.2978	-87.7978	-0.2188	-11.8225	Not Analyzed	-8.4745	-4.764
KH2012:KH.C1.274	MyTF	-1.4181	-55.9129	0.0148	1.1061	Not Analyzed	-5.078	-4.764
KH2012:KH.C8.247	ELK	-2.1780	-111.2186	-0.1709	-10.9920	Not Analyzed	-9.3515	1.7435
KH2012:KH.C4.84	Otx	-2.4872	-188.4332	0.4711	15.4532	Not Analyzed	-3.773	-1.6665

JASPAR 2020 PWM cluster	Max Z-score in cluster	Max Z-score in cluster
POU3F1	17.55	POU2F2
SP3	16.34	SP4
TFAP2B	15.29	TFAP2E

NP (a) 1/2:V/VI

KH2012 gene model	TF common name	Log2FC Sibling Cluster	-Log(pvalue) Sibling Cluster	Log2FC Parent Cluster	-Log(pvalue) Parent Cluster	DE in Imai	Matched PWM z-score	PWM Cluster z-score
KH2012:KH.L57.25	FoxC	1.3711	30.4426	1.3500	84.6722	Yes	4.1045	6.9645
KH2012:KH.L39.1	FoxH-b	1.1852	22.3688	0.8586	24.8722	No	-1.74	3.4855
KH2012:KH.C7.43	AP-2-like2	0.8287	14.1136	0.2912	4.3671	No	No PWM Hits	No PWM Hits
KH2012:KH.C9.717	FoxH-a	0.8234	14.6569	1.5893	99.5026	No	-1.74	3.4855
KH2012:KH.C9.580	RAR	0.5992	11.1327	0.8021	29.6059	No	-4.164	-4.164
KH2012:KH.C7.269	Orphan bHLH1	0.5311	6.1393	0.5835	16.3990	No	No PWM match	No PWM match
KH2012:KH.C7.157	Emc	0.4970	3.1633	0.3161	2.0287	No	No PWM match	No PWM match
KH2012:KH.L13.24	FoxN2/3	0.4811	6.9211	0.5524	14.5316	No	-1.6795	6.9645
KH2012:KH.C2.573	Smad1/5	0.3982	6.0125	0.1662	2.6131	No	No PWM Hits	No PWM Hits
KH2012:KH.C8.162	Zinc Finger (C2H2)-21	0.3317	3.6170	-0.0243	-1.0906	No	No PWM Hits	No PWM Hits
KH2012:KH.C5.50	Zinc Finger (C2H2)-28	0.3010	5.2067	0.7693	32.8337	No	No PWM match	No PWM match
KH2012:KH.C1.1025	NF-X-like	0.2716	3.1865	-0.1125	-2.8882	No	No PWM match	No PWM match
KH2012:KH.C7.127	polybromo	-0.3209	-5.2413	-0.1341	-2.7405	No	No PWM match	No PWM match
KH2012:KH.C10.106	MITF	-0.3571	-4.6537	-0.1568	-3.3425	No	No PWM Hits	No PWM Hits
KH2012:KH.C14.200	ATRX	-0.3660	-6.1899	0.2404	6.2981	No	No PWM match	No PWM match
KH2012:KH.C8.419	HMG2-like	-0.3955	-4.0492	-0.3196	-6.8834	No	No PWM match	No PWM match
KH2012:KH.C10.113	ets/pointed2	-0.4107	-5.9916	0.0408	0.0572	No	-12.567	1.4775
KH2012:KH.C4.366	ERF2	-0.4129	-6.5285	0.1268	0.1576	No	-5.3705	1.4775
KH2012:KH.C3.312	E(spl)/hairy-b	-0.4172	-6.2324	-0.1469	-2.7155	No	No PWM Hits	No PWM Hits
KH2012:KH.C7.523	SoxC	-0.4530	-4.2009	0.3471	2.9509	No	No PWM match	No PWM match
KH2012:KH.C4.509	Zinc Finger (absent, small, or homeotic discs 1)	-0.4539	-6.5683	-0.2187	-3.9231	No	No PWM match	No PWM match
KH2012:KH.C3.348	E12/E47	-0.7200	-16.1015	0.2607	5.8430	No	No PWM Hits	No PWM Hits
KH2012:KH.C4.84	Otx	-0.7217	-18.4794	1.2308	45.6853	No	3.4855	3.4855
KH2012:KH.S816.1	ZicL	-1.5564	-34.1520	-0.8560	-19.0074	No	-2.6885	0.36
KH2012:KH.C8.247	ELK	-2.2194	-40.5574	-1.9075	-67.7247	No	-6.15	1.4775
KH2012:KH.C1.274	MyTF	-2.7992	-51.9338	-0.1349	-4.1301	No	-5.51	0.36

JASPAR 2020 PWM cluster	Max Z-score in cluster	Max Z-score in cluster
TFAP2B	16.92	TFAP2E
NR3C1	16.05	NR3C2

NP (a) 1/2:III/IV

KH2012 gene model	TF common name	Log2FC Sibling Cluster	-Log(pvalue) Sibling Cluster	Log2FC Parent Cluster	-Log(pvalue) Parent Cluster	DE in Imai	Matched PWM z-score	PWM Cluster z-score
KH2012:KH.C1.274	MyTF	2.7992	51.9338	2.6643	88.2468	No	No PWM Hits	11.6
KH2012:KH.C8.247	ELK	2.2194	40.5574	0.3118	4.7330	No	7.475	19.1495
KH2012:KH.S816.1	ZicL	1.5564	34.1520	0.7004	15.7190	Yes	3.1685	11.6
KH2012:KH.C4.84	Otx	0.7217	18.4794	1.9525	57.9226	No	-3.682	6.466
KH2012:KH.C3.348	E12/E47	0.7200	16.1015	0.9808	30.7038	No	No PWM Hits	No PWM Hits
KH2012:KH.C4.509	Zinc Finger (absent, small, or homeotic discs 1)	0.4539	6.5683	0.2352	2.7899	No	No PWM match	No PWM match
KH2012:KH.C7.523	SoxC	0.4530	4.2009	0.8002	13.6404	No	No PWM match	No PWM match
KH2012:KH.C3.312	E(spl)/hairy-b	0.4172	6.2324	0.2704	3.1985	No	No PWM Hits	No PWM Hits
KH2012:KH.C4.366	ERF2	0.4129	6.5285	0.5397	11.7152	No	8.6925	19.1495
KH2012:KH.C10.113	ets/pointed2	0.4107	5.9916	0.4516	9.3698	No	16.2115	19.1495
KH2012:KH.C8.419	HMG2-like	0.3955	4.0492	0.0759	0.2653	No	No PWM match	No PWM match
KH2012:KH.C14.200	ATRX	0.3660	6.1899	0.6064	18.5133	No	No PWM match	No PWM match
KH2012:KH.C10.106	MITF	0.3571	4.6537	0.2003	1.5387	No	No PWM Hits	No PWM Hits
KH2012:KH.C7.127	polybromo	0.3209	5.2413	0.1868	3.1032	No	No PWM match	No PWM match
KH2012:KH.C1.1025	NF-X-like	-0.2716	-3.1865	-0.3841	-10.0589	No	No PWM match	No PWM match
KH2012:KH.C5.50	Zinc Finger (C2H2)-28	-0.3010	-5.2067	0.4683	11.2854	No	No PWM match	No PWM match
KH2012:KH.C8.162	Zinc Finger (C2H2)-21	-0.3317	-3.6170	-0.3560	-8.0174	No	No PWM Hits	No PWM Hits
KH2012:KH.C2.573	Smad1/5	-0.3982	-6.0125	-0.2319	-2.8191	No	No PWM Hits	No PWM Hits
KH2012:KH.L13.24	FoxN2/3	-0.4811	-6.9211	0.0713	0.2689	No	1.8815	4.37
KH2012:KH.C7.157	Emc	-0.4970	-3.1633	-0.1809	-1.3919	No	No PWM match	No PWM match
KH2012:KH.C7.269	Orphan bHLH1	-0.5311	-6.1393	0.0525	0.1592	No	No PWM match	No PWM match
KH2012:KH.C9.580	RAR	-0.5992	-11.1327	0.2029	0.9242	No	3.488	10.694
KH2012:KH.C9.717	FoxH-a	-0.8234	-14.6569	0.7659	45.1696	No	1.9225	6.466
KH2012:KH.C7.43	AP-2-like2	-0.8287	-14.1136	-0.5375	-9.8598	No	No PWM Hits	No PWM Hits
KH2012:KH.L39.1	FoxH-b	-1.1852	-22.3688	-0.3265	-1.8399	No	1.9225	6.466
KH2012:KH.L57.25	FoxC	-1.3711	-30.4426	-0.0211	-0.9475	No	-4.2665	4.37

JASPAR 2020 PWM cluster	Max Z-score in cluster	Max Z-score in cluster
ELK3	19.15	ETV1
E2F7	17.97	E2F8
ZNF282	15.27	ZNF282

Neural (a)

KH2012 gene model	TF common name	Log2FC Sibling Cluster	-Log(pvalue) Sibling Cluster	DE in Imai	Matched PWM z-score	PWM Cluster z-score
KH2012:KH.C4.84	Otx	0.8333	72.7302	Yes	1.306	9.6045
KH2012:KH.C8.247	ELK	0.7300	42.0388	Yes	6.2165	20.1345
KH2012:KH.C4.182	Zinc Finger (C3H)	0.7090	54.6601	No	No PWM match	No PWM match
KH2012:KH.S544.3	DMRT1	0.6776	47.6519	No	0.217	0.217
KH2012:KH.L39.1	FoxH-b	-0.4047	-14.2280	No	6.71	9.6045
KH2012:KH.C7.157	Emc	-1.0858	-68.2257	No	No PWM match	No PWM match

JASPAR 2020 PWM cluster	Max Z-score in cluster	Max Z-score in cluster
HSF2	56.62	ZBTB26
GCM2	40.47	CENPB
Stat5a::Stat5b	20.66	NFATC1
ELK3	20.13	ETS1
E2F7	18.45	E2F2
Zic1::Zic2	17.59	ZKSCAN1
NFKB2	16.24	REL
POU4F1	15.12	POU4F2

Neural (a)

KH2012 gene model	TF common name	Log2FC Sibling Cluster	-Log(pvalue) Sibling Cluster	Log2FC Parent Cluster	-Log(pvalue) Parent Cluster	DE in Imai	Matched PWM z-score	PWM Cluster z-score
KH2012:KH.C8.247	ELK	2.3142	181.1927	1.5649	91.6480	No	20.4155	30.962
KH2012:KH.S544.3	DMRT1	1.3524	146.7600	0.6164	34.3155	Yes	-3.129	-3.129
KH2012:KH.C4.182	Zinc Finger (C3H)	1.3404	112.7477	1.1090	62.1451	No	No PWM match	No PWM match
KH2012:KH.C4.84	Otx	1.2276	117.1020	0.2228	4.9255	Yes	-1.737	6.922
KH2012:KH.S816.1	ZicL	0.9639	58.6941	1.1481	51.4378	No	2.043	8.711
KH2012:KH.C7.523	SoxC	0.6696	52.2414	0.5235	21.8078	No	No PWM match	No PWM match
KH2012:KH.C4.366	ERF2	0.5676	33.3507	0.3648	11.1075	No	15.529	30.962
KH2012:KH.C13.22	Zinc Finger (C2H2)-2	-0.3572	-11.7739	-0.0332	-0.3079	No	0.616	7.004
KH2012:KH.C9.580	RAR	-0.3622	-15.3520	0.2465	7.0312	No	-3.345	-2.8705
KH2012:KH.L4.17	Zinc Finger (C2H2)-18	-0.4052	-15.3898	0.0078	0.5134	No	-3.38	-3.38
KH2012:KH.C7.157	Emc	-0.5105	-23.1007	1.3480	39.8716	No	No PWM match	No PWM match
KH2012:KH.C1.99	SoxB1	-0.5548	-30.5887	0.0021	0.4582	No	No PWM match	No PWM match
KH2012:KH.C7.43	AP-2-like2	-0.6172	-37.6185	0.1417	2.3517	No	No PWM Hits	No PWM Hits
KH2012:KH.C1.159	E(spl)/hairy-a	-0.6779	-38.4790	0.0047	0.4681	No	No PWM Hits	No PWM Hits
KH2012:KH.L39.1	FoxH-b	-0.7741	-47.6850	0.6984	18.2362	No	1.2695	6.922
KH2012:KH.C11.485	Irx-B	-1.2003	-116.9921	0.0153	0.9377	No	No PWM Hits	No PWM Hits

JASPAR 2020 PWM cluster	Max Z-score in cluster	Max Z-score in cluster
ELK3	30.96	ETS1
Esrrg	23.73	ESRRA
ARNT2	16.35	HES1
Arid5a	15.77	Arid5a
BHLHA15_var_2	15.16	PKNOX2

NP (a) 3:V/VI

KH2012 gene model	TF common name	Log2FC Sibling Cluster	-Log(pvalue) Sibling Cluster	Log2FC Parent Cluster	-Log(pvalue) Parent Cluster	DE in Imai	Matched PWM z-score	PWM Cluster z-score
KH2012:KH.L39.1	FoxH-b	1.2630	11.0816	0.8276	9.6999	No	-5.8705	6.1475
KH2012:KH.C9.580	RAR	1.0674	13.1416	1.3275	23.6203	No	4.377	4.377
KH2012:KH.C1.99	SoxB1	0.8866	6.9065	0.1694	0.6113	No	No PWM match	No PWM match
KH2012:KH.C2.573	Smad1/5	0.7611	8.4054	0.4128	5.2255	No	No PWM Hits	No PWM Hits
KH2012:KH.C9.717	FoxH-a	0.6491	4.2905	1.0431	48.2443	No	-5.8705	6.1475
KH2012:KH.C14.586	Zinc Finger (BTB/C2H2)	0.5622	3.2825	0.1514	0.8634	No	-4.9675	-4.9675
KH2012:KH.C9.561	Zinc Finger (C2H2)-9	0.5443	5.3435	0.5941	20.0514	No	-3.61	-2.0165
KH2012:KH.C14.520	Orphan Fox-1	0.4235	9.3827	0.3374	9.2514	No	No PWM Hits	No PWM Hits
KH2012:KH.C14.178	HMG1/2	0.2532	3.2984	0.6355	16.0928	No	No PWM match	No PWM match
KH2012:KH.C1.274	MyTF	-0.2775	-3.2870	-0.1349	-1.6578	No	-2.615	-1.0035
KH2012:KH.C3.697	Zinc Finger (C2H2)-5	-0.2858	-3.2783	-0.9706	-13.2939	No	No PWM Hits	No PWM Hits
KH2012:KH.C3.165	Zinc Finger (C2H2)-26	-0.3219	-3.4693	-0.5519	-7.1828	No	No PWM Hits	No PWM Hits
KH2012:KH.C10.604	Zinc Finger (C2H2)-3	-0.3258	-3.2250	-0.2724	-2.7079	No	-3.5755	-3.5755
KH2012:KH.C13.22	Zinc Finger (C2H2)-2	-0.3626	-3.2951	-0.7511	-9.7323	No	5.1995	11.0845
KH2012:KH.C14.448	AP4	-0.3870	-3.1010	0.0742	0.2282	No	0.819	9.8845
KH2012:KH.C10.49	Bcl3	-0.3890	-3.1140	-0.3061	-2.7643	No	No PWM match	No PWM match
KH2012:KH.C1.27	LAG1-like1	-0.4150	-3.3141	-0.4623	-4.4253	No	No PWM match	No PWM match
KH2012:KH.C7.207	FoxP	-0.4475	-5.2967	-0.0487	-0.9634	No	No PWM Hits	No PWM Hits
KH2012:KH.C1.661	MAD	-0.4507	-3.5649	0.2588	1.0858	No	No PWM match	No PWM match
KH2012:KH.C3.651	IRF-like5	-0.4581	-3.9260	-0.7026	-8.3680	No	No PWM match	No PWM match
KH2012:KH.C2.687	VDR-a	-0.4608	-4.0671	-0.2922	-2.9576	No	No PWM match	No PWM match
KH2012:KH.C5.50	Zinc Finger (C2H2)-28	-0.4678	-5.9445	0.3350	5.9779	No	No PWM match	No PWM match
KH2012:KH.C2.725	Su(H)/RBP-J	-0.5785	-6.7094	-0.4515	-6.0238	No	No PWM Hits	No PWM Hits
KH2012:KH.C1.397	NGFI-A binding protein 43832	-0.5995	-4.6984	-0.5628	-6.6708	No	No PWM match	No PWM match
KH2012:KH.C3.17	ROR	-0.6106	-5.7826	-0.1089	-1.2759	No	-2.667	2.3525
KH2012:KH.C4.182	Zinc Finger (C3H)	-0.6189	-6.0867	-0.7076	-8.2475	No	No PWM match	No PWM match
KH2012:KH.C7.523	SoxC	-0.7270	-9.4902	-0.5801	-6.2169	No	No PWM match	No PWM match
KH2012:KH.C3.348	E12/E47	-0.8619	-10.2214	0.0431	0.3887	No	No PWM Hits	No PWM Hits
KH2012:KH.C3.312	E(spl)/hairy-b	-1.2051	-10.8731	-0.2355	-1.7259	No	No PWM Hits	No PWM Hits
KH2012:KH.C4.366	ERF2	-1.2895	-12.8732	-0.4603	-4.6142	No	-0.171	1.185
KH2012:KH.C10.113	ets/pointed2	-1.3518	-16.0747	-0.5281	-7.6304	No	-6.5265	1.185
KH2012:KH.C4.84	Otx	-2.0319	-21.1430	0.5344	6.4026	No	6.1475	6.1475
KH2012:KH.S816.1	ZicL	-2.1155	-22.2052	-1.2020	-12.2708	No	-3.927	-1.0035
KH2012:KH.C3.751	Snail	-2.6194	-21.5929	0.0223	0.3787	No	No PWM Hits	No PWM Hits
KH2012:KH.C8.247	ELK	-2.8594	-21.8436	-2.0533	-27.2565	No	-1.91	1.185

JASPAR 2020 PWM cluster	Max Z-score in cluster	Max Z-score in cluster

NP (a) 3:III/IV

KH2012 gene model	TF common name	Log2FC Sibling Cluster	-Log(pvalue) Sibling Cluster	Log2FC Parent Cluster	-Log(pvalue) Parent Cluster	DE in Imai	Matched PWM z-score	PWM Cluster z-score
KH2012:KH.C8.247	ELK	2.8594	21.8436	0.8061	24.1298	No	3.2645	18.661
KH2012:KH.C3.751	Snail	2.6194	21.5929	2.6417	100.4557	No	No PWM Hits	No PWM Hits
KH2012:KH.S816.1	ZicL	2.1155	22.2052	0.9134	20.0592	Yes	7.5875	13.3775
KH2012:KH.C4.84	Otx	2.0319	21.1430	2.5663	46.2353	No	-9.6725	10.3865
KH2012:KH.C10.113	ets/pointed2	1.3518	16.0747	0.8238	23.1550	No	12.673	18.661
KH2012:KH.C4.366	ERF2	1.2895	12.8732	0.8292	15.5903	No	0.2275	18.661
KH2012:KH.C3.312	E(spl)/hairy-b	1.2051	10.8731	0.9696	21.3846	No	No PWM Hits	No PWM Hits
KH2012:KH.C3.348	E12/E47	0.8619	10.2214	0.9050	21.5050	No	No PWM Hits	No PWM Hits
KH2012:KH.C7.523	SoxC	0.7270	9.4902	0.1469	2.7367	No	No PWM match	No PWM match
KH2012:KH.C4.182	Zinc Finger (C3H)	0.6189	6.0867	-0.0887	-0.0853	No	No PWM match	No PWM match
KH2012:KH.C3.17	ROR	0.6106	5.7826	0.5017	8.4154	No	4.6105	11.637
KH2012:KH.C1.397	NGFI-A binding protein 43832	0.5995	4.6984	0.0367	0.1496	No	No PWM match	No PWM match
KH2012:KH.C2.725	Su(H)/RBP-J	0.5785	6.7094	0.1270	1.0953	No	No PWM Hits	No PWM Hits
KH2012:KH.C5.50	Zinc Finger (C2H2)-28	0.4678	5.9445	0.8028	22.1515	No	No PWM match	No PWM match
KH2012:KH.C2.687	VDR-a	0.4608	4.0671	0.1686	1.5040	No	No PWM match	No PWM match
KH2012:KH.C3.651	IRF-like5	0.4581	3.9260	-0.2445	-2.2269	No	No PWM match	No PWM match
KH2012:KH.C1.661	MAD	0.4507	3.5649	0.7095	15.1948	No	No PWM match	No PWM match
KH2012:KH.C7.207	FoxP	0.4475	5.2967	0.3988	17.5237	No	No PWM Hits	No PWM Hits
KH2012:KH.C1.27	LAG1-like1	0.4150	3.3141	-0.0472	-0.0252	No	No PWM match	No PWM match
KH2012:KH.C10.49	Bcl3	0.3890	3.1140	0.0830	0.8054	No	No PWM match	No PWM match
KH2012:KH.C14.448	AP4	0.3870	3.1010	0.4613	10.2718	No	-1.151	15.6235
KH2012:KH.C13.22	Zinc Finger (C2H2)-2	0.3626	3.2951	-0.3886	-4.2870	No	-7.018	7.904
KH2012:KH.C10.604	Zinc Finger (C2H2)-3	0.3258	3.2250	0.0533	0.8101	No	9.594	9.594
KH2012:KH.C3.165	Zinc Finger (C2H2)-26	0.3219	3.4693	-0.2299	-2.0655	No	No PWM Hits	No PWM Hits
KH2012:KH.C3.697	Zinc Finger (C2H2)-5	0.2858	3.2783	-0.6849	-10.6393	No	No PWM Hits	No PWM Hits
KH2012:KH.C1.274	MyTF	0.2775	3.2870	0.1426	3.1105	No	No PWM Hits	13.3775
KH2012:KH.C14.178	HMG1/2	-0.2532	-3.2984	0.3824	8.9582	No	No PWM match	No PWM match
KH2012:KH.C14.520	Orphan Fox-1	-0.4235	-9.3827	-0.0860	-0.8368	No	No PWM Hits	No PWM Hits
KH2012:KH.C9.561	Zinc Finger (C2H2)-9	-0.5443	-5.3435	0.0498	1.3199	No	9.0945	9.0945
KH2012:KH.C14.586	Zinc Finger (BTB/C2H2)	-0.5622	-3.2825	-0.4109	-4.9500	No	9.0595	9.0595
KH2012:KH.C9.717	FoxH-a	-0.6491	-4.2905	0.3940	21.7792	No	10.3865	10.3865
KH2012:KH.C2.573	Smad1/5	-0.7611	-8.4054	-0.3484	-3.8772	No	No PWM Hits	No PWM Hits
KH2012:KH.C1.99	SoxB1	-0.8866	-6.9065	-0.7172	-10.8681	No	No PWM match	No PWM match
KH2012:KH.C9.580	RAR	-1.0674	-13.1416	0.2602	4.0915	No	-5.17	3.069
KH2012:KH.L39.1	FoxH-b	-1.2630	-11.0816	-0.4355	-3.1392	No	10.3865	10.3865

JASPAR 2020 PWM cluster	Max Z-score in cluster	Max Z-score in cluster
ZBED1	24.25	ZBTB33
ARNT2	22.97	SOHLH2
NFKB2	21.15	MZF1
ELK3	18.66	SPIB
NR1I2	17.32	NR1I2
PRDM1	16.46	PRDM1
BHLHA15_var_2	15.62	Twist2

Posterior Epidermis

KH2012 gene model	TF common name	Log2FC Sibling Cluster	-Log(pvalue) Sibling Cluster	Log2FC Parent Cluster	-Log(pvalue) Parent Cluster	DE in Imai	Matched PWM z-score	PWM Cluster z-score
KH2012:KH.L142.14	Emx	0.9493	36.8430	0.9491	94.1951	Not Analyzed	3.272	9.2735
KH2012:KH.C11.485	Irx-B	0.6551	22.2530	0.5588	36.6990	Not Analyzed	No PWM Hits	No PWM Hits
KH2012:KH.C9.580	RAR	0.4888	16.0740	0.8891	96.1940	Not Analyzed	-2.227	13.931
KH2012:KH.L39.1	FoxH-b	0.4659	16.3141	-0.0485	-0.4679	Not Analyzed	-0.3845	-0.3845
KH2012:KH.L13.24	FoxN2/3	0.4176	18.1724	0.7133	95.5370	Not Analyzed	-0.102	15.7135
KH2012:KH.C1.159	E(spl)/hairy-a	0.3867	8.1083	0.6373	35.9598	Not Analyzed	No PWM Hits	No PWM Hits
KH2012:KH.C9.561	Zinc Finger (C2H2)-9	0.3373	8.4518	0.1845	3.6082	Not Analyzed	-1.0365	-1.0365
KH2012:KH.C14.520	Orphan Fox-1	0.2937	12.6026	0.1801	10.6336	Not Analyzed	No PWM Hits	No PWM Hits
KH2012:KH.L4.17	Zinc Finger (C2H2)-18	0.2937	8.0610	0.3886	25.7128	Not Analyzed	3.81	3.81
KH2012:KH.C7.269	Orphan bHLH1	-0.2789	-5.5368	0.0149	5.0167	Not Analyzed	No PWM match	No PWM match
KH2012:KH.C4.366	ERF2	-0.3326	-8.7761	-0.0715	-3.8480	Not Analyzed	-5.564	-0.586
KH2012:KH.S816.1	ZicL	-0.3973	-21.5401	-0.0652	-5.9079	Not Analyzed	-3.745	11.4315
KH2012:KH.L8.11	Tbx6a	-0.4689	-49.0921	-0.0980	-11.0838	Not Analyzed	No PWM match	No PWM match
KH2012:KH.C3.312	E(spl)/hairy-b	-0.5201	-10.1067	0.1697	2.7245	Not Analyzed	No PWM Hits	No PWM Hits
KH2012:KH.C7.472	Hox12	-0.7473	-62.4901	0.0767	6.0535	Not Analyzed	No PWM match	No PWM match
KH2012:KH.C8.247	ELK	-1.9714	-150.4527	-0.3239	-18.4511	Not Analyzed	-9.244	-0.586
KH2012:KH.C6.129	Neurogenin	-2.0082	-94.2950	-0.0226	-4.9973	Not Analyzed	No PWM Hits	No PWM Hits

JASPAR 2020 PWM cluster	Max Z-score in cluster	Max Z-score in cluster
POU3F1	18.45	POU2F2
TFAP2B	17.85	TFAP2E
NFIL3	16.51	NFIL3
Foxf1	15.71	FOXB1

Epidermis (b)

KH2012 gene model	TF common name	Log2FC Sibling Cluster	-Log(pvalue) Sibling Cluster	DE in Imai	Matched PWM z-score	PWM Cluster z-score
KH2012:KH.C1.159	E(spl)/hairy-a	0.3985	8.1204	No	No PWM Hits	No PWM Hits
KH2012:KH.C7.269	Orphan bHLH1	0.3626	4.2438	No	No PWM match	No PWM match
KH2012:KH.C2.376	UBF	0.3060	6.1291	No	No PWM match	No PWM match
KH2012:KH.L173.5	Zinc Finger (C2H2)-30	0.2970	4.2071	No	-5.3065	-5.3065
KH2012:KH.C10.92	Baf57	0.2932	3.9769	No	No PWM match	No PWM match
KH2012:KH.L20.26	FoxO	0.2633	4.4457	No	0.8475	4.9805
KH2012:KH.L165.2	HIV TAT specific factor 1 (HTATSF1)	0.2563	3.1197	No	No PWM match	No PWM match
KH2012:KH.C1.1016	GCNF	-0.2790	-6.1524	No	No PWM match	No PWM match
KH2012:KH.C4.366	ERF2	-0.2969	-4.2628	No	-0.723	1.1625
KH2012:KH.C14.105	CNOT7/8	-0.3120	-5.6923	No	No PWM match	No PWM match
KH2012:KH.C12.116	Pax258-B	-0.3250	-4.6178	No	No PWM Hits	No PWM Hits
KH2012:KH.C14.586	Zinc Finger (BTB/C2H2)	-0.3638	-5.2469	No	-1.997	-1.997
KH2012:KH.C14.278	Zinc Finger (C2H2)-34	-0.3672	-7.5855	No	No PWM Hits	No PWM Hits
KH2012:KH.C2.957	msxb	-0.5830	-73.2531	No	-4.0515	4.2195
KH2012:KH.C7.157	Emc	-0.6688	-13.4351	Yes	No PWM match	No PWM match
KH2012:KH.C11.485	Irx-B	-0.7891	-37.2282	No	No PWM Hits	No PWM Hits
KH2012:KH.C8.247	ELK	-0.8817	-38.1342	No	-4.3375	1.1625
KH2012:KH.C4.84	Otx	-0.9127	-40.5739	No	-1.2815	-0.9615

JASPAR 2020 PWM cluster	Max Z-score in cluster	Max Z-score in cluster

Epidermis (b)

KH2012 gene model	TF common name	Log2FC Sibling Cluster	-Log(pvalue) Sibling Cluster	Log2FC Parent Cluster	-Log(pvalue) Parent Cluster	DE in Imai	Matched PWM z-score	PWM Cluster z-score
KH2012:KH.C13.22	Zinc Finger (C2H2)-2	0.9468	68.5708	0.7854	118.3848	No	0.1275	13.432
KH2012:KH.L39.1	FoxH-b	0.8853	46.3871	1.1672	169.2340	No	-3.86	-0.651
KH2012:KH.C7.43	AP-2-like2	0.7219	43.8830	0.8117	121.7644	Yes	No PWM Hits	No PWM Hits
KH2012:KH.C1.159	E(spl)/hairy-a	0.6796	32.9136	0.5599	62.0625	No	No PWM Hits	No PWM Hits
KH2012:KH.C7.269	Orphan bHLH1	0.5461	30.1771	0.0875	5.8624	No	No PWM match	No PWM match
KH2012:KH.C14.520	Orphan Fox-1	0.5284	50.8329	0.6422	157.8614	No	No PWM Hits	No PWM Hits
KH2012:KH.S164.12	SoxB2	0.4868	27.7119	0.4498	73.8197	Yes	4.033	15.598
KH2012:KH.C1.99	SoxB1	0.4831	21.8702	0.5010	54.1797	No	No PWM match	No PWM match
KH2012:KH.L4.17	Zinc Finger (C2H2)-18	0.3464	10.7703	0.2081	7.8584	No	10.637	10.637
KH2012:KH.C9.561	Zinc Finger (C2H2)-9	0.3222	15.4306	0.3274	46.8884	No	-0.1955	3.905
KH2012:KH.C14.278	Zinc Finger (C2H2)-34	-0.2690	-8.8185	-0.0662	-1.2020	No	No PWM Hits	No PWM Hits
KH2012:KH.C4.182	Zinc Finger (C3H)	-0.3242	-21.8321	0.0307	3.6262	No	No PWM match	No PWM match
KH2012:KH.L96.87	Tbx2/3	-0.4622	-41.0861	0.0739	12.5861	No	-2.636	3.5445
KH2012:KH.C3.230	Smad6/7	-0.5119	-31.6973	0.1686	15.1263	No	No PWM match	No PWM match
KH2012:KH.C7.157	Emc	-0.7146	-16.2784	0.8187	60.9695	No	No PWM match	No PWM match
KH2012:KH.C4.366	ERF2	-0.7263	-50.3432	0.0262	1.8456	No	-10.7935	-0.06
KH2012:KH.S816.1	ZicL	-0.8216	-66.9108	-0.0971	-4.3653	No	-2.6345	5.559
KH2012:KH.C11.485	Irx-B	-1.1399	-78.8473	0.1785	19.3886	No	No PWM Hits	No PWM Hits
KH2012:KH.C4.84	Otx	-1.2274	-137.7462	-0.3109	-14.3845	No	-5.529	-0.651
KH2012:KH.C8.247	ELK	-1.7508	-118.3519	0.1097	1.3101	No	-14.351	-0.06
KH2012:KH.C2.957	msxb	-2.0953	-207.1596	0.0592	5.9439	No	5.6745	8.357

JASPAR 2020 PWM cluster	Max Z-score in cluster	Max Z-score in cluster
ZNF143	23.62	ZNF143
POU3F1	23.54	POU2F3
TFAP2B	23.30	Znf423
Nr2f6	22.54	THRB
SOX8	15.60	Sox6
POU4F1	15.32	BARX2

Tail tip Epidermis

KH2012 gene model	TF common name	Log2FC Sibling Cluster	-Log(pvalue) Sibling Cluster	Log2FC Parent Cluster	-Log(pvalue) Parent Cluster	DE in Imai	Matched PWM z-score	PWM Cluster z-score
KH2012:KH.C6.129	Neurogenin	2.0082	94.2950	1.9856	69.5679	Not Analyzed	No PWM Hits	No PWM Hits
KH2012:KH.C8.247	ELK	1.9714	150.4527	1.6475	92.9586	Not Analyzed	12.1545	19.3595
KH2012:KH.C7.472	Hox12	0.7473	62.4901	0.8240	83.7812	Not Analyzed	No PWM match	No PWM match
KH2012:KH.C3.312	E(spl)/hairy-b	0.5201	10.1067	0.6898	16.2750	Not Analyzed	No PWM Hits	No PWM Hits
KH2012:KH.L8.11	Tbx6a	0.4689	49.0921	0.3709	18.7010	Not Analyzed	No PWM match	No PWM match
KH2012:KH.S816.1	ZicL	0.3973	21.5401	0.3321	7.6933	Not Analyzed	6.9285	6.9285
KH2012:KH.C4.366	ERF2	0.3326	8.7761	0.2611	4.2059	Not Analyzed	6.7915	19.3595
KH2012:KH.C7.269	Orphan bHLH1	0.2789	5.5368	0.2937	1.6591	Not Analyzed	No PWM match	No PWM match
KH2012:KH.L4.17	Zinc Finger (C2H2)-18	-0.2937	-8.0610	0.0949	1.7671	Not Analyzed	-4.0495	-4.0495
KH2012:KH.C14.520	Orphan Fox-1	-0.2937	-12.6026	-0.1136	-3.9397	Not Analyzed	No PWM Hits	No PWM Hits
KH2012:KH.C9.561	Zinc Finger (C2H2)-9	-0.3373	-8.4518	-0.1528	-4.5232	Not Analyzed	2.0015	2.2205
KH2012:KH.C1.159	E(spl)/hairy-a	-0.3867	-8.1083	0.2506	1.8322	Not Analyzed	No PWM Hits	No PWM Hits
KH2012:KH.L13.24	FoxN2/3	-0.4176	-18.1724	0.2956	7.1737	Not Analyzed	-0.175	4.687
KH2012:KH.L39.1	FoxH-b	-0.4659	-16.3141	-0.5144	-17.2873	Not Analyzed	0.8265	17.963
KH2012:KH.C9.580	RAR	-0.4888	-16.0740	0.4003	10.7577	Not Analyzed	2.105	2.105
KH2012:KH.C11.485	Irx-B	-0.6551	-22.2530	-0.0963	-2.2775	Not Analyzed	No PWM Hits	No PWM Hits
KH2012:KH.L142.14	Emx	-0.9493	-36.8430	-0.0002	-0.2847	Not Analyzed	-3.418	3.713

JASPAR 2020 PWM cluster	Max Z-score in cluster	Max Z-score in cluster
FOSL2::JUNB_var_2_	20.96	ATF7
ELK3	19.36	ELK4
ARNT2	18.58	SOHLH2
SP3	18.18	KLF11
PITX3	17.96	Dmbx1
FOS::JUNB	16.96	MAFA
E2F7	16.83	E2F1
Nr2f6	16.34	TCF7

NP (b) posterior

KH2012 gene model	TF common name	Log2FC Sibling Cluster	-Log(pvalue) Sibling Cluster	Log2FC Parent Cluster	-Log(pvalue) Parent Cluster	DE in Imai	Matched PWM z-score	PWM Cluster z-score
KH2012:KH.C14.408	Cdx	1.2303	21.7741	1.2303	68.4349	Not Analyzed	No PWM Hits	No PWM Hits
KH2012:KH.C3.59	Zinc finger protein-like 1	0.5554	5.0295	0.0328	0.1507	Not Analyzed	No PWM match	No PWM match
KH2012:KH.C1.669	Zinc Finger (C2H2)-25	0.5406	6.2765	0.5831	10.2113	Not Analyzed	4.509	4.509
KH2012:KH.C14.307	MyoD	0.5339	6.0515	0.9720	55.0033	Not Analyzed	No PWM Hits	No PWM Hits
KH2012:KH.C7.472	Hox12	0.5313	7.4848	0.6109	29.6191	Not Analyzed	No PWM match	No PWM match
KH2012:KH.C7.111	Transcription initiation protein SPT4 homolog	0.4181	3.2169	-0.3555	-4.3083	Not Analyzed	No PWM match	No PWM match
KH2012:KH.C2.290	GABPα	0.4026	3.2953	0.2847	2.7819	Not Analyzed	22.2675	23.658
KH2012:KH.C12.67	four and a half LIM domains1/2/3	0.3870	4.4302	0.2361	1.7979	Not Analyzed	No PWM match	No PWM match
KH2012:KH.L153.114	Zinc Finger (C2H2)-19	0.3662	4.0941	0.1165	0.8952	Not Analyzed	3.5945	3.5945
KH2012:KH.C5.362	CBF1 interacting corepressor-like	0.3607	4.5951	0.3061	5.4756	Not Analyzed	No PWM match	No PWM match
KH2012:KH.C11.620	ubiquitin-like, containing PHD and RING finger domains, 1	0.3479	4.6934	0.0261	0.2817	Not Analyzed	No PWM match	No PWM match
KH2012:KH.C13.51	REV-ERB	0.3412	5.2849	-0.0254	-0.2486	Not Analyzed	-4.429	13.281
KH2012:KH.S215.9	Pbx	0.3403	3.3469	0.1817	2.0526	Not Analyzed	7.157	21.999
KH2012:KH.L7.6	Sex comb on midleg-like1	0.3259	3.1232	0.2054	1.9888	Not Analyzed	No PWM match	No PWM match
KH2012:KH.S2060.1	Zinc Finger-like2	0.3259	3.6981	0.1152	1.4356	Not Analyzed	3.5945	3.5945
KH2012:KH.C12.26	Smad2/3b	0.2996	5.2383	-0.0224	-0.0281	Not Analyzed	8.4125	8.4125
KH2012:KH.S654.3	Tbx6b/c/d	0.2956	4.4963	0.4078	20.8585	Not Analyzed	No PWM match	No PWM match
KH2012:KH.L153.110	Zinc Finger (C2H2)-7	0.2894	3.3069	-0.0756	-0.2955	Not Analyzed	No PWM Hits	No PWM Hits
KH2012:KH.C4.430	Prox-B	0.2538	4.4379	0.0558	0.8275	Not Analyzed	No PWM match	No PWM match
KH2012:KH.L122.11	metastasis associated protein	-0.3850	-3.7003	-0.5619	-8.9293	Not Analyzed	No PWM match	No PWM match
KH2012:KH.C3.152	SSRP	-0.4146	-3.5537	-0.3055	-3.4424	Not Analyzed	No PWM match	No PWM match
KH2012:KH.C11.706	Mlx	-0.4200	-3.0409	-0.2814	-1.6890	Not Analyzed	0.273	7.2655
KH2012:KH.C12.370	FoxK	-0.4996	-3.8687	-0.6078	-7.1017	Not Analyzed	No PWM Hits	No PWM Hits
KH2012:KH.C1.661	MAD	-0.5384	-4.5330	-0.0692	-0.3334	Not Analyzed	No PWM match	No PWM match
KH2012:KH.C1.99	SoxB1	-0.6807	-5.6601	-0.3926	-3.4857	Not Analyzed	No PWM match	No PWM match
KH2012:KH.C9.580	RAR	-0.7106	-6.9469	-0.1134	-1.0272	Not Analyzed	-8.744	5.8705
KH2012:KH.L39.1	FoxH-b	-0.7629	-6.6932	-0.6665	-4.3789	Not Analyzed	4.6575	11.1805
KH2012:KH.C7.157	Emc	-1.4986	-13.7727	-1.4117	-11.9753	Not Analyzed	No PWM match	No PWM match

JASPAR 2020 PWM cluster	Max Z-score in cluster	Max Z-score in cluster
TP73	37.22	TP63
OVOL2	33.21	MYBL2
GATA2	29.69	Mecom
FOS::JUNB	26.95	MAF
NFIL3	25.60	CEBPA
IRF8	24.01	IRF3
ELK3	23.66	ETS1
GRHL2	22.93	SIX2
NKX2-3	22.43	MSANTD3
GCM2	22.16	CENPB
MEIS1_var_2_	22.00	PBX2
Foxf1	20.86	Foxf1
PAX1	18.42	PAX1
NFKB2	17.36	NFKB1
GLIS2	17.16	ZIC5
POU3F1	17.08	Pou5f1::Sox2
TBX21	15.93	SREBF2

NP (b) anterior

KH2012 gene model	TF common name	Log2FC Sibling Cluster	-Log(pvalue) Sibling Cluster	Log2FC Parent Cluster	-Log(pvalue) Parent Cluster	DE in Imai	Matched PWM z-score	PWM Cluster z-score
KH2012:KH.C7.157	Emc	1.4986	13.7727	0.0869	0.7460	Not Analyzed	No PWM match	No PWM match
KH2012:KH.L39.1	FoxH-b	0.7629	6.6932	0.0964	1.3353	Not Analyzed	-2.353	3.4025
KH2012:KH.C9.580	RAR	0.7106	6.9469	0.5972	9.6253	Not Analyzed	1.0805	1.0805
KH2012:KH.C1.99	SoxB1	0.6807	5.6601	0.2881	2.6966	Not Analyzed	No PWM match	No PWM match
KH2012:KH.C1.661	MAD	0.5384	4.5330	0.4692	6.4741	Not Analyzed	No PWM match	No PWM match
KH2012:KH.C12.370	FoxK	0.4996	3.8687	-0.1082	-0.6816	Not Analyzed	No PWM Hits	No PWM Hits
KH2012:KH.C11.706	Mlx	0.4200	3.0409	0.1387	1.2821	Not Analyzed	-0.373	10.52
KH2012:KH.C3.152	SSRP	0.4146	3.5537	0.1092	0.7929	Not Analyzed	No PWM match	No PWM match
KH2012:KH.L122.11	metastasis associated protein	0.3850	3.7003	-0.1769	-1.7799	Not Analyzed	No PWM match	No PWM match
KH2012:KH.C4.430	Prox-B	-0.2538	-4.4379	-0.1979	-3.4146	Not Analyzed	No PWM match	No PWM match
KH2012:KH.L153.110	Zinc Finger (C2H2)-7	-0.2894	-3.3069	-0.3650	-5.2116	Not Analyzed	No PWM Hits	No PWM Hits
KH2012:KH.S654.3	Tbx6b/c/d	-0.2956	-4.4963	0.1122	2.2918	Not Analyzed	No PWM match	No PWM match
KH2012:KH.C12.26	Smad2/3b	-0.2996	-5.2383	-0.3219	-5.4921	Not Analyzed	-1.451	6.924
KH2012:KH.L7.6	Sex comb on midleg-like1	-0.3259	-3.1232	-0.1205	-1.4722	Not Analyzed	No PWM match	No PWM match
KH2012:KH.S2060.1	Zinc Finger-like2	-0.3259	-3.6981	-0.2107	-2.3745	Not Analyzed	-1.752	-1.752
KH2012:KH.S215.9	Pbx	-0.3403	-3.3469	-0.1586	-1.3627	Not Analyzed	-3.283	-3.283
KH2012:KH.C13.51	REV-ERB	-0.3412	-5.2849	-0.3666	-5.2993	Not Analyzed	2.527	5.5745
KH2012:KH.C11.620	ubiquitin-like, containing PHD and RING finger domains, 1	-0.3479	-4.6934	-0.3217	-4.9829	Not Analyzed	No PWM match	No PWM match
KH2012:KH.C5.362	CBF1 interacting corepressor-like	-0.3607	-4.5951	-0.0546	-0.5145	Not Analyzed	No PWM match	No PWM match
KH2012:KH.L153.114	Zinc Finger (C2H2)-19	-0.3662	-4.0941	-0.2497	-3.8467	Not Analyzed	-1.752	-1.752
KH2012:KH.C12.67	four and a half LIM domains1/2/3	-0.3870	-4.4302	-0.1509	-2.7574	Not Analyzed	No PWM match	No PWM match
KH2012:KH.C2.290	GABPα	-0.4026	-3.2953	-0.1180	-1.3922	Not Analyzed	-9.056	2.5645
KH2012:KH.C7.111	Transcription initiation protein SPT4 homolog	-0.4181	-3.2169	-0.7736	-14.8927	Not Analyzed	No PWM match	No PWM match
KH2012:KH.C7.472	Hox12	-0.5313	-7.4848	0.0796	1.3806	Not Analyzed	No PWM match	No PWM match
KH2012:KH.C14.307	MyoD	-0.5339	-6.0515	0.4381	20.3279	Not Analyzed	No PWM Hits	No PWM Hits
KH2012:KH.C1.669	Zinc Finger (C2H2)-25	-0.5406	-6.2765	0.0424	0.4246	Not Analyzed	-2.2405	-2.2405
KH2012:KH.C3.59	Zinc finger protein-like 1	-0.5554	-5.0295	-0.5226	-7.5218	Not Analyzed	No PWM match	No PWM match
KH2012:KH.C14.408	Cdx	-1.2303	-21.7741	0.0000	0.0000	Not Analyzed	No PWM Hits	No PWM Hits

JASPAR 2020 PWM cluster	Max Z-score in cluster	Max Z-score in cluster

Neural (b)

KH2012 gene model	TF common name	Log2FC Sibling Cluster	-Log(pvalue) Sibling Cluster	DE in Imai	Matched PWM z-score	PWM Cluster z-score
KH2012:KH.C4.84	Otx	0.9127	40.5739	Yes	1.9625	9.115
KH2012:KH.C8.247	ELK	0.8817	38.1342	Yes	7.0505	20.948
KH2012:KH.C11.485	Irx-B	0.7891	37.2282	Yes	No PWM Hits	No PWM Hits
KH2012:KH.C7.157	Emc	0.6688	13.4351	No	No PWM match	No PWM match
KH2012:KH.C2.957	msxb	0.5830	73.2531	Yes	5.7105	8.6195
KH2012:KH.C14.278	Zinc Finger (C2H2)-34	0.3672	7.5855	No	No PWM Hits	No PWM Hits
KH2012:KH.C14.586	Zinc Finger (BTB/C2H2)	0.3638	5.2469	No	3.4785	3.4785
KH2012:KH.C12.116	Pax258-B	0.3250	4.6178	No	No PWM Hits	No PWM Hits
KH2012:KH.C14.105	CNOT7/8	0.3120	5.6923	No	No PWM match	No PWM match
KH2012:KH.C4.366	ERF2	0.2969	4.2628	No	1.3	20.948
KH2012:KH.C1.1016	GCNF	0.2790	6.1524	No	No PWM match	No PWM match
KH2012:KH.L165.2	HIV TAT specific factor 1 (HTATSF1)	-0.2563	-3.1197	No	No PWM match	No PWM match
KH2012:KH.L20.26	FoxO	-0.2633	-4.4457	No	-1.0735	8.386
KH2012:KH.C10.92	Baf57	-0.2932	-3.9769	No	No PWM match	No PWM match
KH2012:KH.L173.5	Zinc Finger (C2H2)-30	-0.2970	-4.2071	No	21.065	21.065
KH2012:KH.C2.376	UBF	-0.3060	-6.1291	No	No PWM match	No PWM match
KH2012:KH.C7.269	Orphan bHLH1	-0.3626	-4.2438	No	No PWM match	No PWM match
KH2012:KH.C1.159	E(spl)/hairy-a	-0.3985	-8.1204	No	No PWM Hits	No PWM Hits

JASPAR 2020 PWM cluster	Max Z-score in cluster	Max Z-score in cluster
SCRT1	31.19	SCRT2
HINFP	28.65	HINFP
PBX3	27.29	PBX3
SP3	21.58	ZBTB14
CTCF	21.07	CTCF
ELK3	20.95	ELK4
ZBED1	15.84	ZBED1

Neural (b)

KH2012 gene model	TF common name	Log2FC Sibling Cluster	-Log(pvalue) Sibling Cluster	Log2FC Parent Cluster	-Log(pvalue) Parent Cluster	DE in Imai	Matched PWM z-score	PWM Cluster z-score
KH2012:KH.C2.957	msxb	2.0953	207.1596	1.5715	33.7620	Yes	-5.3305	5.818
KH2012:KH.C8.247	ELK	1.7508	118.3519	0.9787	13.9149	No	23.341	23.341
KH2012:KH.C4.84	Otx	1.2274	137.7462	0.0038	0.3108	No	5.0975	8.5595
KH2012:KH.C11.485	Irx-B	1.1399	78.8473	0.5293	6.7584	No	No PWM Hits	No PWM Hits
KH2012:KH.S816.1	ZicL	0.8216	66.9108	0.8356	12.4014	No	3.2255	10.6575
KH2012:KH.C4.366	ERF2	0.7263	50.3432	0.4556	9.4053	No	17.0915	23.341
KH2012:KH.C7.157	Emc	0.7146	16.2784	0.8645	7.5725	No	No PWM match	No PWM match
KH2012:KH.C3.230	Smad6/7	0.5119	31.6973	0.4717	8.2286	No	No PWM match	No PWM match
KH2012:KH.L96.87	Tbx2/3	0.4622	41.0861	0.5160	10.5226	No	2.6315	9.876
KH2012:KH.C4.182	Zinc Finger (C3H)	0.3242	21.8321	0.1153	1.9478	No	No PWM match	No PWM match
KH2012:KH.C14.278	Zinc Finger (C2H2)-34	0.2690	8.8185	-0.1644	-1.1921	No	No PWM Hits	No PWM Hits
KH2012:KH.C9.561	Zinc Finger (C2H2)-9	-0.3222	-15.4306	0.0632	0.8239	No	0.247	0.247
KH2012:KH.L4.17	Zinc Finger (C2H2)-18	-0.3464	-10.7703	-0.1222	-1.6818	No	-5.432	-5.432
KH2012:KH.C1.99	SoxB1	-0.4831	-21.8702	-0.0336	-0.2273	No	No PWM match	No PWM match
KH2012:KH.S164.12	SoxB2	-0.4868	-27.7119	-0.0148	-0.4124	No	-3.1565	3.3115
KH2012:KH.C14.520	Orphan Fox-1	-0.5284	-50.8329	0.1348	0.9270	No	No PWM Hits	No PWM Hits
KH2012:KH.C7.269	Orphan bHLH1	-0.5461	-30.1771	-0.0960	-2.3915	No	No PWM match	No PWM match
KH2012:KH.C1.159	E(spl)/hairy-a	-0.6796	-32.9136	0.2789	5.7325	No	No PWM Hits	No PWM Hits
KH2012:KH.C7.43	AP-2-like2	-0.7219	-43.8830	0.1202	1.0719	No	No PWM Hits	No PWM Hits
KH2012:KH.L39.1	FoxH-b	-0.8853	-46.3871	0.4967	3.7705	No	3.985	8.5595
KH2012:KH.C13.22	Zinc Finger (C2H2)-2	-0.9468	-68.5708	-0.1148	-1.2711	No	-0.07	9.921

JASPAR 2020 PWM cluster	Max Z-score in cluster	Max Z-score in cluster
BHLHA15_var_2	24.23	PKNOX2
ELK3	23.34	ELK1
ZNF135	21.96	ZNF135
Esrrg	19.82	ESRRA
OVOL2	15.09	MYBL2

Dorsal midline tail epidermis

KH2012 gene model	TF common name	Log2FC Sibling Cluster	-Log(pvalue) Sibling Cluster	Log2FC Parent Cluster	-Log(pvalue) Parent Cluster	DE in Imai	Matched PWM z-score	PWM Cluster z-score
KH2012:KH.C7.157	Emc	1.4793	31.3794	1.0625	30.4186	Not Analyzed	No PWM match	No PWM match
KH2012:KH.L39.1	FoxH-b	1.1702	26.7187	0.9674	30.0871	Not Analyzed	1.0805	6.717
KH2012:KH.C1.1016	GCNF	0.8289	12.2925	0.9200	23.8765	Not Analyzed	No PWM match	No PWM match
KH2012:KH.C7.269	Orphan bHLH1	0.7045	13.1695	0.6618	21.0759	Not Analyzed	No PWM match	No PWM match
KH2012:KH.L4.17	Zinc Finger (C2H2)-18	0.6391	10.3072	0.4730	10.2016	Not Analyzed	0.575	0.575
KH2012:KH.C7.43	AP-2-like2	0.5666	8.1894	0.0930	0.2017	Not Analyzed	No PWM Hits	No PWM Hits
KH2012:KH.C11.485	Irx-B	0.5617	8.8682	-0.7176	-16.7663	Not Analyzed	No PWM Hits	No PWM Hits
KH2012:KH.C9.580	RAR	0.5390	7.9151	0.8544	25.5630	Not Analyzed	-2.429	-2.429
KH2012:KH.C2.573	Smad1/5	0.5005	7.4012	-0.0929	-0.6527	Not Analyzed	No PWM Hits	No PWM Hits
KH2012:KH.C9.717	FoxH-a	0.4456	9.8333	0.4743	20.6229	Not Analyzed	1.0805	6.717
KH2012:KH.C7.782	POU-like	0.4270	8.5419	0.1171	2.1266	Not Analyzed	No PWM match	No PWM match
KH2012:KH.C14.178	HMG1/2	0.4061	12.9998	0.6265	33.7392	Not Analyzed	No PWM match	No PWM match
KH2012:KH.C1.99	SoxB1	0.3902	5.6810	0.4067	8.5584	Not Analyzed	No PWM match	No PWM match
KH2012:KH.L108.4	CREB/ATF-a	0.3732	7.2026	0.2865	5.5887	Not Analyzed	6.019	10.352
KH2012:KH.C9.561	Zinc Finger (C2H2)-9	0.3647	4.0078	0.5413	15.5035	Not Analyzed	0.725	0.725
KH2012:KH.S654.3	Tbx6b/c/d	-0.2756	-10.3059	-0.0211	-1.3055	Not Analyzed	No PWM match	No PWM match
KH2012:KH.L155.15	SWI/SNF	-0.2805	-3.4869	-0.1249	-1.3946	Not Analyzed	No PWM match	No PWM match
KH2012:KH.C6.71	Tcf/Lef	-0.2865	-6.2351	-0.4116	-11.3459	Not Analyzed	No PWM match	No PWM match
KH2012:KH.S455.6	MEF2	-0.2942	-3.8496	-0.2323	-4.7550	Not Analyzed	No PWM Hits	No PWM Hits
KH2012:KH.C4.390	NFYA	-0.2986	-4.9618	-0.1557	-3.1376	Not Analyzed	No PWM Hits	No PWM Hits
KH2012:KH.C1.27	LAG1-like1	-0.2987	-3.8453	-0.3360	-6.2630	Not Analyzed	No PWM match	No PWM match
KH2012:KH.C8.143	PPAR	-0.3159	-3.3955	-0.4208	-8.7918	Not Analyzed	No PWM Hits	2.676
KH2012:KH.L154.23	Trh	-0.3257	-4.3283	-0.4443	-10.8792	Not Analyzed	No PWM match	No PWM match
KH2012:KH.C1.669	Zinc Finger (C2H2)-25	-0.3593	-4.5324	-0.0450	-0.8439	Not Analyzed	-0.88	-0.88
KH2012:KH.C9.616	Lmx	-0.4890	-14.8921	0.0000	0.0000	Not Analyzed	No PWM Hits	No PWM Hits
KH2012:KH.C3.17	ROR	-0.5191	-5.7038	0.1819	2.3643	Not Analyzed	1.8515	7.6465
KH2012:KH.C14.408	Cdx	-0.5900	-13.5315	0.1007	5.5418	Not Analyzed	No PWM Hits	No PWM Hits
KH2012:KH.C2.957	msxb	-0.6397	-16.4443	1.2022	47.9108	Not Analyzed	9.428	12.29
KH2012:KH.C14.307	MyoD	-0.7063	-25.0059	0.0000	0.0000	Not Analyzed	No PWM Hits	No PWM Hits
KH2012:KH.S816.1	ZicL	-1.6641	-47.7591	-0.8669	-23.7760	Not Analyzed	-7.955	-2.268
KH2012:KH.C8.247	ELK	-2.5159	-52.3058	-1.6482	-47.2068	Not Analyzed	-9.183	2.1615
KH2012:KH.C3.751	Snail	-2.5905	-61.2396	0.0633	2.7454	Not Analyzed	No PWM Hits	No PWM Hits

JASPAR 2020 PWM cluster	Max Z-score in cluster	Max Z-score in cluster
POU3F1	15.17	POU2F1

anterior TLC

KH2012 gene model	TF common name	Log2FC Sibling Cluster	-Log(pvalue) Sibling Cluster	Log2FC Parent Cluster	-Log(pvalue) Parent Cluster	DE in Imai	Matched PWM z-score	PWM Cluster z-score
KH2012:KH.C7.157	Emc	3.5529	24.9029	2.6887	23.3201	Not Analyzed	No PWM match	No PWM match
KH2012:KH.C1.159	E(spl)/hairy-a	1.3219	12.8792	0.8511	9.0443	Not Analyzed	No PWM Hits	No PWM Hits
KH2012:KH.C1.99	SoxB1	1.1747	13.0132	0.7579	7.9318	Not Analyzed	No PWM match	No PWM match
KH2012:KH.L4.17	Zinc Finger (C2H2)-18	0.9881	10.4282	1.4189	16.7559	Not Analyzed	-0.581	-0.581
KH2012:KH.C1.1116	NoTrlc	0.9601	10.2183	0.6133	6.2125	Not Analyzed	No PWM match	No PWM match
KH2012:KH.C4.182	Zinc Finger (C3H)	0.9051	8.0106	2.6406	24.6213	Not Analyzed	No PWM match	No PWM match
KH2012:KH.C11.313	FoxA-a	0.8740	7.4906	1.1550	12.1742	Not Analyzed	No PWM Hits	No PWM Hits
KH2012:KH.C14.448	AP4	0.8289	12.4578	0.7366	10.3794	Not Analyzed	-1.3665	6.5035
KH2012:KH.C10.92	Baf57	0.6496	3.3804	0.0173	0.0687	Not Analyzed	No PWM match	No PWM match
KH2012:KH.C9.717	FoxH-a	0.4753	7.3753	0.5070	13.3232	Not Analyzed	-4.76	5.98
KH2012:KH.C7.556	ATF2/7	0.4581	3.3741	0.5091	5.7264	Not Analyzed	9.534	10.987
KH2012:KH.C3.732	LAG1-like 2	0.3028	3.5268	0.2007	1.8492	Not Analyzed	No PWM match	No PWM match
KH2012:KH.C14.178	HMG1/2	-0.2595	-3.8458	-0.0872	-0.7574	Not Analyzed	No PWM match	No PWM match
KH2012:KH.C3.230	Smad6/7	-0.2895	-3.2626	-0.2313	-2.5074	Not Analyzed	No PWM match	No PWM match
KH2012:KH.C6.129	Neurogenin	-0.3155	-3.2584	-0.6329	-6.6157	Not Analyzed	No PWM Hits	No PWM Hits
KH2012:KH.C3.773	Ci-mT	-0.3168	-3.2541	-0.9155	-9.4877	Not Analyzed	No PWM match	No PWM match
KH2012:KH.C1.516	HMG20	-0.3195	-3.2651	-0.4289	-2.7854	Not Analyzed	No PWM match	No PWM match
KH2012:KH.C3.877	Zinc Finger (C2H2)-1	-0.3921	-4.1393	-0.7244	-7.7109	Not Analyzed	No PWM Hits	No PWM Hits
KH2012:KH.C5.213	ARNT	-0.4082	-3.5052	-0.3873	-2.2916	Not Analyzed	-0.2245	8.9155
KH2012:KH.L122.11	metastasis associated protein	-0.4341	-3.7635	-0.8575	-9.7794	Not Analyzed	No PWM match	No PWM match
KH2012:KH.C10.148	Fli/ERG3	-0.4389	-4.2623	-0.1009	-1.2311	Not Analyzed	-4.0485	2.642
KH2012:KH.C4.504	FoxM	-0.4464	-4.0878	-0.5255	-4.4797	Not Analyzed	-4.569	3.934
KH2012:KH.C1.1016	GCNF	-0.5081	-5.3505	-0.1009	-1.4500	Not Analyzed	No PWM match	No PWM match
KH2012:KH.C1.669	Zinc Finger (C2H2)-25	-0.5110	-3.6644	-0.3489	-2.8978	Not Analyzed	-2.568	-2.568
KH2012:KH.C6.22	Smad2/3a	-0.5179	-4.0253	-0.7244	-7.7757	Not Analyzed	No PWM match	No PWM match
KH2012:KH.C1.27	LAG1-like1	-0.5247	-4.6237	-0.5644	-4.2920	Not Analyzed	No PWM match	No PWM match
KH2012:KH.C10.604	Zinc Finger (C2H2)-3	-0.5337	-5.2001	-0.3320	-3.2327	Not Analyzed	1.0395	1.0395
KH2012:KH.L41.39	Orphan bHLH-4	-0.5519	-4.7439	-0.4533	-3.8537	Not Analyzed	No PWM match	No PWM match
KH2012:KH.C4.84	Otx	-0.5525	-5.4011	0.3315	2.2389	Not Analyzed	1.413	5.98
KH2012:KH.C3.152	SSRP	-0.5850	-4.7131	-0.5497	-5.0175	Not Analyzed	No PWM match	No PWM match
KH2012:KH.C3.697	Zinc Finger (C2H2)-5	-0.5880	-4.6211	-1.1750	-12.8181	Not Analyzed	No PWM Hits	No PWM Hits
KH2012:KH.C3.312	E(spl)/hairy-b	-0.6348	-4.9688	-0.1159	-0.2293	Not Analyzed	No PWM Hits	No PWM Hits
KH2012:KH.C5.202	TWIST-like-2	-0.6630	-3.3930	0.0532	1.0912	Not Analyzed	No PWM match	No PWM match
KH2012:KH.C10.113	ets/pointed2	-0.6890	-7.2377	-0.6496	-4.8067	Not Analyzed	-5.188	2.642
KH2012:KH.C1.275	STAT-b	-0.7396	-7.5884	-0.5503	-5.5088	Not Analyzed	No PWM match	No PWM match
KH2012:KH.C4.539	Fli/ERG1	-0.9175	-9.6441	-0.0208	-0.4795	Not Analyzed	-2.9075	2.642
KH2012:KH.C8.247	ELK	-1.9536	-15.6321	-0.7244	-5.7154	Not Analyzed	-2.6945	2.642
KH2012:KH.C5.416	TWIST-like-1a/b	-2.0550	-17.1601	-0.3009	-2.9341	Not Analyzed	No PWM match	No PWM match
KH2012:KH.C1.274	MyTF	-2.3020	-21.0950	-0.7340	-5.2026	Not Analyzed	10.97	10.97

JASPAR 2020 PWM cluster	Max Z-score in cluster	Max Z-score in cluster

A7.6

A7.6

KH2012 gene model	TF common name	Log2FC Sibling Cluster	-Log(pvalue) Sibling Cluster	DE in Imai	Matched PWM z-score	PWM Cluster z-score
KH2012:KH.C1.1116	NoTrlc	1.2872	47.5438	Yes	No PWM match	No PWM match
KH2012:KH.C11.485	Irx-B	0.9699	29.3901	No	No PWM Hits	No PWM Hits
KH2012:KH.C4.341	FoxB	0.8429	36.2956	Yes	5.3475	14.246
KH2012:KH.L39.1	FoxH-b	0.6023	11.5088	Yes	-2.781	2.092
KH2012:KH.C1.274	MyTF	0.4464	19.1451	No	No PWM Hits	3.314
KH2012:KH.C5.90	core-binding factor, β subunit	0.3292	3.8220	No	No PWM match	No PWM match
KH2012:KH.C4.366	ERF2	0.3075	5.0917	No	-6.7205	10.961
KH2012:KH.C4.504	FoxM	0.2671	4.0215	No	6.705	14.246
KH2012:KH.L87.58	Zinc Finger (C2H2)-4	0.2546	3.3338	No	No PWM Hits	No PWM Hits
KH2012:KH.C10.338	TTF1	-0.2886	-5.6683	No	No PWM Hits	No PWM Hits
KH2012:KH.C3.751	Snail	-0.3383	-5.0494	No	No PWM Hits	No PWM Hits
KH2012:KH.C4.182	Zinc Finger (C3H)	-0.3597	-4.0128	No	No PWM match	No PWM match
KH2012:KH.C11.313	FoxA-a	-0.4864	-5.7832	No	No PWM Hits	No PWM Hits
KH2012:KH.C3.312	E(spl)/hairy-b	-0.5060	-6.4242	No	No PWM Hits	No PWM Hits
KH2012:KH.C8.247	ELK	-0.6761	-7.2333	No	-7.73	10.961
KH2012:KH.S215.4	Lhx3	-0.8746	-12.3900	No	-5.5045	7.3165

JASPAR 2020 PWM cluster	Max Z-score in cluster	Max Z-score in cluster
FOS::JUNB	32.57	Nfe2l2
SOX8	31.32	SOX21
IRF8	27.26	IRF2
HOXD10	20.32	CDX1
RFX2	20.11	RFX2
POU4F1	19.12	HNF1B
BHLHA15_var_2	17.47	NEUROD2
SP3	17.18	KLF14
RARA_var_2	16.26	RARA::RXRA
MAFG	15.68	MAFG

KH2012 gene model	TF common name	Log2FC Sibling Cluster	-Log(pvalue) Sibling Cluster	Log2FC Parent Cluster	-Log(pvalue) Parent Cluster	DE in Imai	Matched PWM z-score	PWM Cluster z-score
KH2012:KH.C1.1116	NoTrlc	1.2388	17.8563	0.5317	5.5486	Yes	No PWM match	No PWM match
KH2012:KH.C1.274	MyTF	1.0881	15.5661	0.6229	5.0981	No	2.057	3.741
KH2012:KH.L39.1	FoxH-b	0.8988	12.3923	-0.0889	-0.7687	Yes	-5.1075	1.1115
KH2012:KH.C4.341	FoxB	0.8573	14.7571	-0.1043	-1.1134	Yes	-5.385	4.114
KH2012:KH.C1.99	SoxB1	0.8029	12.1050	0.1067	0.3596	Yes	No PWM match	No PWM match
KH2012:KH.C14.520	Orphan Fox-1	0.7061	16.6750	0.1743	3.5739	No	No PWM Hits	No PWM Hits
KH2012:KH.C4.366	ERF2	0.7000	8.7362	0.4176	3.9609	No	-3.8635	-1.0185
KH2012:KH.C2.573	Smad1/5	0.4891	5.9527	-0.1764	-0.4200	No	No PWM Hits	No PWM Hits
KH2012:KH.C3.773	Ci-mT	0.4623	4.7831	-0.7738	-9.5567	No	No PWM match	No PWM match
KH2012:KH.C14.105	CNOT7/8	0.4586	4.9555	-0.2110	-2.8628	No	No PWM match	No PWM match
KH2012:KH.C7.157	Emc	0.4384	6.6051	0.7090	7.9317	No	No PWM match	No PWM match
KH2012:KH.C11.485	Irx-B	0.4315	5.8331	-0.5525	-6.8022	No	No PWM Hits	No PWM Hits
KH2012:KH.C7.269	Orphan bHLH1	0.3415	3.6237	0.2389	1.4597	No	No PWM match	No PWM match
KH2012:KH.S899.4	BTF3	0.3039	4.0825	-0.1206	-0.9859	No	No PWM match	No PWM match
KH2012:KH.C5.416	TWIST-like-1a/b	0.3009	4.5751	0.3009	4.9920	Yes	No PWM match	No PWM match
KH2012:KH.C2.1038	DEAF-1	-0.2636	-6.9504	0.0412	1.2105	No	No PWM match	No PWM match
KH2012:KH.C2.957	msxb	-0.2710	-3.6091	0.1009	1.2104	No	2.492	7.289
KH2012:KH.C13.110	Zinc Finger (C2H2)-22	-0.2786	-3.4210	-0.0615	-0.1913	No	2.4845	10.0345
KH2012:KH.C11.735	Zinc Finger (C2H2)-29	-0.2841	-3.2065	-0.0456	-0.0697	No	0.112	0.112
KH2012:KH.C4.390	NFYA	-0.2896	-5.5631	-0.1825	-1.3763	No	No PWM Hits	No PWM Hits
KH2012:KH.C3.17	ROR	-0.2973	-3.2515	-0.1585	-1.8897	No	-1.1025	5.6025
KH2012:KH.L20.34	Mnt-like	-0.3002	-5.8916	0.0916	0.7495	No	No PWM match	No PWM match
KH2012:KH.L7.6	Sex comb on midleg-like1	-0.3060	-5.1646	-0.3484	-3.5974	No	No PWM match	No PWM match
KH2012:KH.L60.12	transcription factor Dp-1	-0.3132	-4.1729	-0.5189	-7.0467	No	No PWM match	No PWM match
KH2012:KH.L34.7	Zinc Finger (absent, small, or homeotic discs 2)	-0.3272	-3.6182	0.1394	1.2141	No	No PWM match	No PWM match
KH2012:KH.C1.677	Smcx/SMCY/PLU-1/RBP-2 like 1	-0.3347	-5.5924	0.1602	0.3528	No	No PWM match	No PWM match
KH2012:KH.C14.200	ATRX	-0.3348	-3.5907	-0.2719	-3.7732	No	No PWM match	No PWM match
KH2012:KH.C7.556	ATF2/7	-0.3352	-6.9821	0.0635	1.1177	No	10.9295	17.604
KH2012:KH.C7.207	FoxP	-0.3388	-11.6406	-0.1096	-3.9617	No	No PWM Hits	No PWM Hits
KH2012:KH.L24.23	Myc	-0.3457	-3.4786	-0.0964	-0.0550	No	No PWM Hits	No PWM Hits
KH2012:KH.C2.961	Zinc Finger (C2H2)-12	-0.3657	-3.4405	-0.1121	-1.1706	No	0.112	0.112
KH2012:KH.S1272.2	ALR-like	-0.3671	-5.5066	-0.0964	-0.3462	No	No PWM match	No PWM match
KH2012:KH.C7.215	NFκBp105	-0.3690	-3.6780	0.0963	0.4865	No	2.292	2.292
KH2012:KH.C3.751	Snail	-0.3966	-10.1235	0.1009	1.6176	No	No PWM Hits	No PWM Hits
KH2012:KH.L173.5	Zinc Finger (C2H2)-30	-0.4061	-4.1744	-0.1927	-1.8741	No	1.5025	1.5025
KH2012:KH.L13.24	FoxN2/3	-0.4467	-7.3012	-0.0211	-0.0552	No	4.114	4.114
KH2012:KH.C1.159	E(spl)/hairy-a	-0.4916	-7.6645	0.4661	2.3484	No	No PWM Hits	No PWM Hits
KH2012:KH.L154.23	Trh	-0.4975	-8.4605	-0.2819	-2.6755	No	No PWM match	No PWM match
KH2012:KH.C4.509	Zinc Finger (absent, small, or homeotic discs 1)	-0.5369	-7.2380	0.0298	0.1963	No	No PWM match	No PWM match
KH2012:KH.C2.687	VDR-a	-0.5723	-9.5866	-0.3922	-5.4209	No	No PWM match	No PWM match
KH2012:KH.C3.230	Smad6/7	-0.6585	-12.1761	0.1217	1.1570	No	No PWM match	No PWM match
KH2012:KH.L4.17	Zinc Finger (C2H2)-18	-0.7987	-8.1519	0.3073	0.8645	No	-6.685	-6.685
KH2012:KH.C11.313	FoxA-a	-0.9936	-18.5645	-0.0869	-0.4093	No	No PWM Hits	No PWM Hits
KH2012:KH.C4.84	Otx	-1.0254	-16.5218	0.8575	9.5868	No	-0.3575	1.1115
KH2012:KH.C4.182	Zinc Finger (C3H)	-1.4141	-15.2711	0.3093	3.7282	No	No PWM match	No PWM match
KH2012:KH.C10.338	TTF1	-1.4361	-36.3826	0.0000	0.0000	No	No PWM Hits	No PWM Hits
KH2012:KH.S215.4	Lhx3	-1.9431	-38.0832	-0.2274	-2.8589	No	-5.6075	7.289
KH2012:KH.C8.247	ELK	-2.4594	-33.1357	0.5200	3.8347	No	-6.631	-1.0185

JASPAR 2020 PWM cluster	Max Z-score in cluster	Max Z-score in cluster
FOSL2::JUNB_var_2	17.60	CREM
Six3	16.19	Six3

posterior TLC

KH2012 gene model	TF common name	Log2FC Sibling Cluster	-Log(pvalue) Sibling Cluster	Log2FC Parent Cluster	-Log(pvalue) Parent Cluster	DE in Imai	Matched PWM z-score	PWM Cluster z-score
KH2012:KH.C1.274	MyTF	2.3020	21.0950	1.5681	32.1669	Not Analyzed	-7.959	11.341
KH2012:KH.C5.416	TWIST-like-1a/b	2.0550	17.1601	1.7542	32.3362	Not Analyzed	No PWM match	No PWM match
KH2012:KH.C8.247	ELK	1.9536	15.6321	1.2292	18.9279	Not Analyzed	4.1475	13.215
KH2012:KH.C4.539	Fli/ERG1	0.9175	9.6441	0.8968	24.4566	Not Analyzed	4.184	13.215
KH2012:KH.C1.275	STAT-b	0.7396	7.5884	0.1893	1.9725	Not Analyzed	No PWM match	No PWM match
KH2012:KH.C10.113	ets/pointed2	0.6890	7.2377	0.0394	0.9532	Not Analyzed	8.0725	13.215
KH2012:KH.C5.202	TWIST-like-2	0.6630	3.3930	0.7162	11.2881	Not Analyzed	No PWM match	No PWM match
KH2012:KH.C3.312	E(spl)/hairy-b	0.6348	4.9688	0.5188	8.2042	Not Analyzed	No PWM Hits	No PWM Hits
KH2012:KH.C3.697	Zinc Finger (C2H2)-5	0.5880	4.6211	-0.5871	-8.9105	Not Analyzed	No PWM Hits	No PWM Hits
KH2012:KH.C3.152	SSRP	0.5850	4.7131	0.0353	0.1555	Not Analyzed	No PWM match	No PWM match
KH2012:KH.C4.84	Otx	0.5525	5.4011	0.8841	18.8893	Not Analyzed	-2.1805	7.5295
KH2012:KH.L41.39	Orphan bHLH-4	0.5519	4.7439	0.0986	0.6239	Not Analyzed	No PWM match	No PWM match
KH2012:KH.C10.604	Zinc Finger (C2H2)-3	0.5337	5.2001	0.2016	2.0253	Not Analyzed	-7.891	-7.891
KH2012:KH.C1.27	LAG1-like1	0.5247	4.6237	-0.0397	-0.2138	Not Analyzed	No PWM match	No PWM match
KH2012:KH.C6.22	Smad2/3a	0.5179	4.0253	-0.2065	-2.1298	Not Analyzed	No PWM match	No PWM match
KH2012:KH.C1.669	Zinc Finger (C2H2)-25	0.5110	3.6644	0.1621	0.8518	Not Analyzed	3.218	3.218
KH2012:KH.C1.1016	GCNF	0.5081	5.3505	0.4072	8.1940	Not Analyzed	No PWM match	No PWM match
KH2012:KH.C4.504	FoxM	0.4464	4.0878	-0.0791	-0.2647	Not Analyzed	7.4815	8.532
KH2012:KH.C10.148	Fli/ERG3	0.4389	4.2623	0.3380	6.3445	Not Analyzed	6.0845	13.215
KH2012:KH.L122.11	metastasis associated protein	0.4341	3.7635	-0.4233	-6.5353	Not Analyzed	No PWM match	No PWM match
KH2012:KH.C5.213	ARNT	0.4082	3.5052	0.0208	0.8076	Not Analyzed	-0.3285	15.452
KH2012:KH.C3.877	Zinc Finger (C2H2)-1	0.3921	4.1393	-0.3323	-4.2620	Not Analyzed	No PWM Hits	No PWM Hits
KH2012:KH.C1.516	HMG20	0.3195	3.2651	-0.1094	-0.0410	Not Analyzed	No PWM match	No PWM match
KH2012:KH.C3.773	Ci-mT	0.3168	3.2541	-0.5987	-8.2131	Not Analyzed	No PWM match	No PWM match
KH2012:KH.C6.129	Neurogenin	0.3155	3.2584	-0.3174	-3.4786	Not Analyzed	No PWM Hits	No PWM Hits
KH2012:KH.C3.230	Smad6/7	0.2895	3.2626	0.0582	0.8075	Not Analyzed	No PWM match	No PWM match
KH2012:KH.C14.178	HMG1/2	0.2595	3.8458	0.1723	3.3630	Not Analyzed	No PWM match	No PWM match
KH2012:KH.C3.732	LAG1-like 2	-0.3028	-3.5268	-0.1021	-1.2885	Not Analyzed	No PWM match	No PWM match
KH2012:KH.C7.556	ATF2/7	-0.4581	-3.3741	0.0511	0.7309	Not Analyzed	-13.8365	-0.807
KH2012:KH.C9.717	FoxH-a	-0.4753	-7.3753	0.0317	1.5036	Not Analyzed	7.5295	7.5295
KH2012:KH.C10.92	Baf57	-0.6496	-3.3804	-0.6322	-7.8867	Not Analyzed	No PWM match	No PWM match
KH2012:KH.C14.448	AP4	-0.8289	-12.4578	-0.0923	-1.5467	Not Analyzed	1.842	11.4645
KH2012:KH.C11.313	FoxA-a	-0.8740	-7.4906	0.2811	3.2560	Not Analyzed	No PWM Hits	No PWM Hits
KH2012:KH.C4.182	Zinc Finger (C3H)	-0.9051	-8.0106	1.7355	31.2453	Not Analyzed	No PWM match	No PWM match
KH2012:KH.C1.1116	NoTrlc	-0.9601	-10.2183	-0.3468	-3.5228	Not Analyzed	No PWM match	No PWM match
KH2012:KH.L4.17	Zinc Finger (C2H2)-18	-0.9881	-10.4282	0.4308	5.7251	Not Analyzed	-2.4235	-2.4235
KH2012:KH.C1.99	SoxB1	-1.1747	-13.0132	-0.4168	-4.6714	Not Analyzed	No PWM match	No PWM match
KH2012:KH.C1.159	E(spl)/hairy-a	-1.3219	-12.8792	-0.4708	-2.7277	Not Analyzed	No PWM Hits	No PWM Hits
KH2012:KH.C7.157	Emc	-3.5529	-24.9029	-0.8641	-13.0799	Not Analyzed	No PWM match	No PWM match

JASPAR 2020 PWM cluster	Max Z-score in cluster	Max Z-score in cluster
NFKB2	23.02	NFKB2
POU3F1	19.26	Pou5f1::Sox2
IRF8	17.48	Stat2
ZNF282	15.91	ZNF282
ARNT2	15.45	MYC

A Endoderm

KH2012 gene model	TF common name	Log2FC Sibling Cluster	-Log(pvalue) Sibling Cluster	Log2FC Parent Cluster	-Log(pvalue) Parent Cluster	DE in Imai	Matched PWM z-score	PWM Cluster z-score
KH2012:KH.C4.182	Zinc Finger (C3H)	1.6845	11.4481	2.1133	24.7287	No	No PWM match	No PWM match
KH2012:KH.C1.1116	NoTrlc	1.2922	8.6138	1.1972	19.7534	No	No PWM match	No PWM match
KH2012:KH.C4.366	ERF2	1.0621	4.8554	0.5530	2.3260	No	4.9545	7.791
KH2012:KH.C1.159	E(spl)/hairy-a	0.6781	4.3931	0.9101	9.2604	No	No PWM Hits	No PWM Hits
KH2012:KH.C4.84	Otx	0.4796	3.5309	2.4180	21.9554	No	12.0955	16.3985
KH2012:KH.C3.177	IRF-like8	0.4504	3.5924	0.4150	5.8547	No	No PWM match	No PWM match
KH2012:KH.C10.106	MITF	0.3301	3.0726	0.2345	2.9797	No	No PWM Hits	No PWM Hits
KH2012:KH.C1.99	SoxB1	0.2801	3.3326	-0.3720	-1.6789	No	No PWM match	No PWM match
KH2012:KH.C11.488	Pax19	0.2801	3.3326	0.2596	9.6769	No	-7.21	-2.834
KH2012:KH.C6.71	Tcf/Lef	-0.3479	-3.2038	-0.2073	-1.5043	No	No PWM match	No PWM match
KH2012:KH.C2.724	small optic lobes homolog-like	-0.3785	-3.0033	-0.2544	-2.4495	No	No PWM match	No PWM match
KH2012:KH.C1.573	p53/p73-a	-0.3859	-3.2894	-0.1635	-1.2600	No	No PWM match	No PWM match
KH2012:KH.S899.4	BTF3	-0.4288	-3.6038	-0.6373	-8.1314	No	No PWM match	No PWM match
KH2012:KH.C11.658	Zinc finger (RING)	-0.4365	-3.3214	-0.1635	-1.2600	No	No PWM match	No PWM match
KH2012:KH.C2.822	E2F1/2/3/6	-0.4381	-3.1609	-0.1915	-1.1351	No	No PWM match	No PWM match
KH2012:KH.C2.725	Su(H)/RBP-J	-0.4424	-3.0810	-0.3321	-1.8197	No	No PWM Hits	No PWM Hits
KH2012:KH.C3.59	Zinc finger protein-like 1	-0.4489	-3.0744	-0.2388	-1.3746	No	No PWM match	No PWM match
KH2012:KH.C11.535	Zinc Finger (C2H2)-17	-0.4551	-3.2221	-0.5525	-3.6359	No	No PWM match	No PWM match
KH2012:KH.C3.751	Snail	-0.4854	-3.4504	-0.1457	-0.3319	No	No PWM Hits	No PWM Hits
KH2012:KH.C10.604	Zinc Finger (C2H2)-3	-0.5250	-3.2458	-0.0238	-0.1670	No	-9.996	-9.996
KH2012:KH.S215.9	Pbx	-0.5317	-5.8875	-0.2446	-1.4751	No	-4.2035	-2.561
KH2012:KH.C7.702	Zinc Finger (BTB/POZ)	-0.5541	-3.6011	-0.4159	-2.6643	No	No PWM Hits	No PWM Hits
KH2012:KH.S1482.3	NFRKB	-0.5634	-3.5893	-0.8701	-8.2034	No	No PWM match	No PWM match
KH2012:KH.L96.11	Groucho2	-0.6753	-4.1715	-0.4822	-3.5715	No	No PWM match	No PWM match

JASPAR 2020 PWM cluster	Max Z-score in cluster	Max Z-score in cluster
ZBED1	24.92	ZBED1
Nr2f6	22.40	LEF1
ZNF143	19.65	ZNF143
TBX19	18.89	TBX19
PITX3	16.40	GSC2
NR1I2	15.83	RXRA::VDR

A Endoderm

KH2012 gene model	TF common name	Log2FC Sibling Cluster	-Log(pvalue) Sibling Cluster	Log2FC Parent Cluster	-Log(pvalue) Parent Cluster	DE in Imai	Matched PWM z-score	PWM Cluster z-score
KH2012:KH.C1.1116	NoTrlc	2.3391	38.4544	0.9765	4.7816	Not Analyzed	No PWM match	No PWM match
KH2012:KH.C2.573	Smad1/5	0.5688	11.3726	0.0435	0.0443	Not Analyzed	No PWM Hits	No PWM Hits
KH2012:KH.C14.520	Orphan Fox-1	0.5056	11.4846	0.3674	2.6088	Not Analyzed	No PWM Hits	No PWM Hits
KH2012:KH.C9.580	RAR	0.4940	9.3583	0.8884	7.4463	Not Analyzed	0.014	0.014
KH2012:KH.C4.366	ERF2	0.4355	5.6283	-0.5762	-2.5530	Not Analyzed	-2.2105	5.0065
KH2012:KH.L24.23	Myc	0.4150	4.7073	0.2065	0.8474	Not Analyzed	No PWM Hits	No PWM Hits
KH2012:KH.L39.1	FoxH-b	0.3446	6.2823	0.4288	3.0700	Not Analyzed	0.4265	5.192
KH2012:KH.S980.1	Zinc finger (PHD)-2	0.2946	3.6663	-0.0959	-0.8075	Not Analyzed	No PWM match	No PWM match
KH2012:KH.C8.419	HMG2-like	0.2782	4.1460	0.4270	3.2590	Not Analyzed	No PWM match	No PWM match
KH2012:KH.C1.99	SoxB1	0.2775	6.5497	0.0783	0.4607	Not Analyzed	No PWM match	No PWM match
KH2012:KH.C3.348	E12/E47	0.2754	3.6461	0.3684	2.7311	Not Analyzed	No PWM Hits	No PWM Hits
KH2012:KH.C11.485	Irx-B	0.2611	3.4000	0.1754	1.3131	Not Analyzed	No PWM Hits	No PWM Hits
KH2012:KH.C14.408	Cdx	-0.2630	-8.9806	0.0365	0.2903	Not Analyzed	No PWM Hits	No PWM Hits
KH2012:KH.L150.2	Orphan Fox-2	-0.2668	-5.4907	-0.2854	-4.7476	Not Analyzed	No PWM Hits	No PWM Hits
KH2012:KH.C12.457	Orphan HMG-1	-0.2725	-3.9755	0.1493	0.5245	Not Analyzed	No PWM match	No PWM match
KH2012:KH.C3.644	C/EBPα/γ	-0.2813	-5.8710	-0.1349	-1.3248	Not Analyzed	No PWM Hits	No PWM Hits
KH2012:KH.L153.85	Zinc Finger (C2H2)-8	-0.2866	-3.7856	-0.4932	-4.4450	Not Analyzed	2.7105	3.6385
KH2012:KH.C1.26	CREB/ATF-c	-0.2917	-3.1530	-0.2779	-2.5412	Not Analyzed	No PWM Hits	No PWM Hits
KH2012:KH.L153.110	Zinc Finger (C2H2)-7	-0.2938	-5.1903	-0.1310	-0.9174	Not Analyzed	No PWM Hits	No PWM Hits
KH2012:KH.S215.4	Lhx3	-0.3000	-5.7702	-1.7043	-24.0661	Not Analyzed	-2.6125	5.847
KH2012:KH.L9.33	E(Y)2	-0.3150	-5.9005	-0.4500	-4.4217	Not Analyzed	No PWM match	No PWM match
KH2012:KH.C14.247	Elf	-0.3151	-4.8961	0.2589	1.5400	Not Analyzed	-0.816	5.0065
KH2012:KH.L153.117	Zinc Finger (C2H2)-14	-0.3428	-9.4669	-0.4516	-3.9333	Not Analyzed	-4.6485	-4.6485
KH2012:KH.C4.85	POU2	-0.3440	-6.2132	-0.6356	-7.4276	Not Analyzed	1.6415	5.833
KH2012:KH.C1.397	NGFI-A binding protein 43832	-0.3484	-4.5199	-0.0274	-0.1468	Not Analyzed	No PWM match	No PWM match
KH2012:KH.C4.84	Otx	-0.3485	-5.5947	-2.0280	-19.8128	Not Analyzed	1.1265	5.192
KH2012:KH.C14.105	CNOT7/8	-0.3563	-6.5893	0.3355	2.1825	Not Analyzed	No PWM match	No PWM match
KH2012:KH.S406.15	Serum response factor	-0.3580	-8.5055	-0.4597	-4.6398	Not Analyzed	-0.393	-0.393
KH2012:KH.C11.313	FoxA-a	-0.3908	-5.6909	-0.5588	-5.9375	Not Analyzed	No PWM Hits	No PWM Hits
KH2012:KH.C10.367	Six36	-0.4369	-8.8910	0.0721	0.8239	Not Analyzed	3.841	3.841
KH2012:KH.S899.4	BTF3	-0.5644	-14.9255	0.3781	2.5697	Not Analyzed	No PWM match	No PWM match
KH2012:KH.C8.396	FoxD-a/b	-0.9757	-13.5478	-0.1618	-2.6284	Not Analyzed	No PWM Hits	No PWM Hits

JASPAR 2020 PWM cluster	Max Z-score in cluster	Max Z-score in cluster
FOSL2::JUNB_var_2	19.47	CREM

Endoderm

KH2012 gene model	TF common name	Log2FC Sibling Cluster	-Log(pvalue) Sibling Cluster	DE in Imai	Matched PWM z-score	PWM Cluster z-score
KH2012:KH.S215.4	Lhx3	0.8746	12.3900	Yes	4.038	10.771
KH2012:KH.C8.247	ELK	0.6761	7.2333	Yes	6.4065	24.528
KH2012:KH.C3.312	E(spl)/hairy-b	0.5060	6.4242	No	No PWM Hits	No PWM Hits
KH2012:KH.C11.313	FoxA-a	0.4864	5.7832	Yes	No PWM Hits	No PWM Hits
KH2012:KH.C4.182	Zinc Finger (C3H)	0.3597	4.0128	Yes	No PWM match	No PWM match
KH2012:KH.C3.751	Snail	0.3383	5.0494	Yes	No PWM Hits	No PWM Hits
KH2012:KH.C10.338	TTF1	0.2886	5.6683	Yes	No PWM Hits	No PWM Hits
KH2012:KH.L87.58	Zinc Finger (C2H2)-4	-0.2546	-3.3338	No	No PWM Hits	No PWM Hits
KH2012:KH.C4.504	FoxM	-0.2671	-4.0215	No	-4.353	3.9785
KH2012:KH.C4.366	ERF2	-0.3075	-5.0917	No	5.4425	24.528
KH2012:KH.C5.90	core-binding factor, β subunit	-0.3292	-3.8220	No	No PWM match	No PWM match
KH2012:KH.C1.274	MyTF	-0.4464	-19.1451	No	No PWM Hits	14.877
KH2012:KH.L39.1	FoxH-b	-0.6023	-11.5088	No	2.32	7.305
KH2012:KH.C4.341	FoxB	-0.8429	-36.2956	No	-3.595	3.9785
KH2012:KH.C11.485	Irx-B	-0.9699	-29.3901	No	No PWM Hits	No PWM Hits
KH2012:KH.C1.1116	NoTrlc	-1.2872	-47.5438	No	No PWM match	No PWM match

JASPAR 2020 PWM cluster	Max Z-score in cluster	Max Z-score in cluster
ELK3	24.53	GABPA

B Endoderm

KH2012 gene model	TF common name	Log2FC Sibling Cluster	-Log(pvalue) Sibling Cluster	Log2FC Parent Cluster	-Log(pvalue) Parent Cluster	DE in Imai	Matched PWM z-score	PWM Cluster z-score
KH2012:KH.L96.11	Groucho2	0.6753	4.1715	0.1930	1.0930	No	No PWM match	No PWM match
KH2012:KH.S1482.3	NFRKB	0.5634	3.5893	-0.3067	-2.3701	No	No PWM match	No PWM match
KH2012:KH.C7.702	Zinc Finger (BTB/POZ)	0.5541	3.6011	0.1382	1.1927	No	No PWM Hits	No PWM Hits
KH2012:KH.S215.9	Pbx	0.5317	5.8875	0.2871	4.7418	No	3.4655	3.4655
KH2012:KH.C10.604	Zinc Finger (C2H2)-3	0.5250	3.2458	0.5011	6.9128	No	18.534	18.534
KH2012:KH.C3.751	Snail	0.4854	3.4504	0.3397	6.2938	No	No PWM Hits	No PWM Hits
KH2012:KH.C11.535	Zinc Finger (C2H2)-17	0.4551	3.2221	-0.0975	-0.1238	No	No PWM match	No PWM match
KH2012:KH.C3.59	Zinc finger protein-like 1	0.4489	3.0744	0.2101	2.2977	No	No PWM match	No PWM match
KH2012:KH.C2.725	Su(H)/RBP-J	0.4424	3.0810	0.1103	1.4080	No	No PWM Hits	No PWM Hits
KH2012:KH.C2.822	E2F1/2/3/6	0.4381	3.1609	0.2466	2.8825	No	No PWM match	No PWM match
KH2012:KH.C11.658	Zinc finger (RING)	0.4365	3.3214	0.2730	3.6135	No	No PWM match	No PWM match
KH2012:KH.S899.4	BTF3	0.4288	3.6038	-0.2084	-2.3545	No	No PWM match	No PWM match
KH2012:KH.C1.573	p53/p73-a	0.3859	3.2894	0.2224	3.3793	No	No PWM match	No PWM match
KH2012:KH.C2.724	small optic lobes homolog-like	0.3785	3.0033	0.1241	0.8793	No	No PWM match	No PWM match
KH2012:KH.C6.71	Tcf/Lef	0.3479	3.2038	0.1406	1.9804	No	No PWM match	No PWM match
KH2012:KH.C1.99	SoxB1	-0.2801	-3.3326	-0.6521	-6.4203	No	No PWM match	No PWM match
KH2012:KH.C11.488	Pax19	-0.2801	-3.3326	-0.0205	-0.5090	No	7.741	8.3285
KH2012:KH.C10.106	MITF	-0.3301	-3.0726	-0.0957	-0.7931	No	No PWM Hits	No PWM Hits
KH2012:KH.C3.177	IRF-like8	-0.4504	-3.5924	-0.0354	-0.2164	No	No PWM match	No PWM match
KH2012:KH.C4.84	Otx	-0.4796	-3.5309	1.9384	25.4887	No	-7.9525	0.9685
KH2012:KH.C1.159	E(spl)/hairy-a	-0.6781	-4.3931	0.2320	2.7332	No	No PWM Hits	No PWM Hits
KH2012:KH.C4.366	ERF2	-1.0621	-4.8554	-0.5090	-4.4046	No	-3.979	6.4745
KH2012:KH.C1.1116	NoTrlc	-1.2922	-8.6138	-0.0949	-0.6594	No	No PWM match	No PWM match
KH2012:KH.C4.182	Zinc Finger (C3H)	-1.6845	-11.4481	0.4288	4.3454	No	No PWM match	No PWM match

JASPAR 2020 PWM cluster	Max Z-score in cluster	Max Z-score in cluster
CTCF	18.53	CTCF
SOX8	16.83	Sox11
Esrrg	15.04	NR6A1

B Endoderm

KH2012 gene model	TF common name	Log2FC Sibling Cluster	-Log(pvalue) Sibling Cluster	Log2FC Parent Cluster	-Log(pvalue) Parent Cluster	DE in Imai	Matched PWM z-score	PWM Cluster z-score
KH2012:KH.C8.396	FoxD-a/b	0.9757	13.5478	0.7720	1.5524	Not Analyzed	No PWM Hits	No PWM Hits
KH2012:KH.S899.4	BTF3	0.5644	14.9255	0.5137	6.5846	Not Analyzed	No PWM match	No PWM match
KH2012:KH.C10.367	Six36	0.4369	8.8910	0.5090	3.8853	Not Analyzed	-5.637	3.43
KH2012:KH.C11.313	FoxA-a	0.3908	5.6909	0.1800	0.7930	Not Analyzed	No PWM Hits	No PWM Hits
KH2012:KH.S406.15	Serum response factor	0.3580	8.5055	-0.0159	-0.0110	Not Analyzed	-4.043	-4.043
KH2012:KH.C14.105	CNOT7/8	0.3563	6.5893	0.4180	3.6921	Not Analyzed	No PWM match	No PWM match
KH2012:KH.C4.84	Otx	0.3485	5.5947	-1.2000	-14.5770	Not Analyzed	-1.704	2.4025
KH2012:KH.C1.397	NGFI-A binding protein 43832	0.3484	4.5199	-0.0441	-0.4242	Not Analyzed	No PWM match	No PWM match
KH2012:KH.C4.85	POU2	0.3440	6.2132	0.1740	0.2424	Not Analyzed	-0.7465	9.642
KH2012:KH.L153.117	Zinc Finger (C2H2)-14	0.3428	9.4669	-0.2317	-1.1904	Not Analyzed	6.936	6.936
KH2012:KH.C14.247	Elf	0.3151	4.8961	0.1761	0.6991	Not Analyzed	5.662	21.692
KH2012:KH.L9.33	E(Y)2	0.3150	5.9005	0.1094	0.7662	Not Analyzed	No PWM match	No PWM match
KH2012:KH.S215.4	Lhx3	0.3000	5.7702	-1.2931	-12.3089	Not Analyzed	4.456	11.9835
KH2012:KH.L153.110	Zinc Finger (C2H2)-7	0.2938	5.1903	0.0673	0.2910	Not Analyzed	No PWM Hits	No PWM Hits
KH2012:KH.C1.26	CREB/ATF-c	0.2917	3.1530	0.1263	0.1788	Not Analyzed	No PWM Hits	No PWM Hits
KH2012:KH.L153.85	Zinc Finger (C2H2)-8	0.2866	3.7856	0.0601	0.0389	Not Analyzed	-4.229	-2.348
KH2012:KH.C3.644	C/EBPα/γ	0.2813	5.8710	-0.2135	-0.8269	Not Analyzed	No PWM Hits	No PWM Hits
KH2012:KH.C12.457	Orphan HMG-1	0.2725	3.9755	0.4866	4.2701	Not Analyzed	No PWM match	No PWM match
KH2012:KH.L150.2	Orphan Fox-2	0.2668	5.4907	0.0809	0.0419	Not Analyzed	No PWM Hits	No PWM Hits
KH2012:KH.C14.408	Cdx	0.2630	8.9806	0.2996	2.7057	Not Analyzed	No PWM Hits	No PWM Hits
KH2012:KH.C11.485	Irx-B	-0.2611	-3.4000	-0.0306	-0.2709	Not Analyzed	No PWM Hits	No PWM Hits
KH2012:KH.C3.348	E12/E47	-0.2754	-3.6461	-0.1839	-1.1913	Not Analyzed	No PWM Hits	No PWM Hits
KH2012:KH.C1.99	SoxB1	-0.2775	-6.5497	0.0809	1.2589	Not Analyzed	No PWM match	No PWM match
KH2012:KH.C8.419	HMG2-like	-0.2782	-4.1460	-0.1072	-1.1744	Not Analyzed	No PWM match	No PWM match
KH2012:KH.S980.1	Zinc finger (PHD)-2	-0.2946	-3.6663	-0.2139	-1.5529	Not Analyzed	No PWM match	No PWM match
KH2012:KH.L39.1	FoxH-b	-0.3446	-6.2823	0.2065	1.4769	Not Analyzed	-0.9305	2.4025
KH2012:KH.L24.23	Myc	-0.4150	-4.7073	-0.1721	-1.3357	Not Analyzed	No PWM Hits	No PWM Hits
KH2012:KH.C4.366	ERF2	-0.4355	-5.6283	0.0503	0.2823	Not Analyzed	3.878	21.692
KH2012:KH.C9.580	RAR	-0.4940	-9.3583	0.3527	1.0323	Not Analyzed	0.3405	4.155
KH2012:KH.C14.520	Orphan Fox-1	-0.5056	-11.4846	-0.4438	-2.2084	Not Analyzed	No PWM Hits	No PWM Hits
KH2012:KH.C2.573	Smad1/5	-0.5688	-11.3726	-0.1859	-1.4473	Not Analyzed	No PWM Hits	No PWM Hits
KH2012:KH.C1.1116	NoTrlc	-2.3391	-38.4544	-0.0704	-2.1735	Not Analyzed	No PWM match	No PWM match

JASPAR 2020 PWM cluster	Max Z-score in cluster	Max Z-score in cluster
ARNT2	30.36	MYCN
GLIS2	22.66	ZIC5
ELK3	21.69	SPI1
BHLHA15_var_2	19.52	OLIG2
MTF1	18.40	MTF1
Plagl1	18.15	PLAGL2
OVOL2	16.45	MYBL1
SMAD3	16.10	ZSCAN29

A7.3 & A7.7

KH2012 gene model	TF common name	Log2FC Sibling Cluster	-Log(pvalue) Sibling Cluster	DE in Imai	Matched PWM z-score	PWM Cluster z-score
KH2012:KH.L128.12	Mnx	1.4244	77.3644	Yes	4.6355	8.7775
KH2012:KH.S215.4	Lhx3	1.3648	71.8561	Yes	4.665	8.7775
KH2012:KH.C8.247	ELK	1.3201	51.9200	Yes	5.2435	13.3495
KH2012:KH.S1404.1	Brachyury	1.1711	70.8956	Yes	1.7545	1.982
KH2012:KH.C11.313	FoxA-a	0.5575	7.3507	Yes	No PWM Hits	No PWM Hits
KH2012:KH.C4.509	Zinc Finger (absent, small, or homeotic discs 1)	0.4979	9.5414	No	No PWM match	No PWM match
KH2012:KH.S1272.2	ALR-like	0.3581	4.9484	No	No PWM match	No PWM match
KH2012:KH.C4.182	Zinc Finger (C3H)	0.3581	4.8832	No	No PWM match	No PWM match
KH2012:KH.C3.312	E(spl)/hairy-b	0.3372	8.0416	No	No PWM Hits	No PWM Hits
KH2012:KH.C14.449	CBP	0.3223	6.8396	No	No PWM match	No PWM match
KH2012:KH.C12.116	Pax258-B	0.3029	6.3070	No	No PWM Hits	No PWM Hits
KH2012:KH.C3.103	RBP1-like	0.2918	8.5341	No	No PWM match	No PWM match
KH2012:KH.C14.105	CNOT7/8	-0.2607	-3.4516	No	No PWM match	No PWM match
KH2012:KH.C11.735	Zinc Finger (C2H2)-29	-0.2852	-5.9628	No	-1.6385	-1.6385
KH2012:KH.C2.290	GABPα	-0.2966	-7.3008	No	10.1325	13.3495
KH2012:KH.C5.50	Zinc Finger (C2H2)-28	-0.2998	-6.9499	No	No PWM match	No PWM match
KH2012:KH.S899.4	BTF3	-0.3064	-8.5329	No	No PWM match	No PWM match
KH2012:KH.L153.110	Zinc Finger (C2H2)-7	-0.3145	-7.2689	No	No PWM Hits	No PWM Hits
KH2012:KH.C9.892	RXR	-0.3690	-9.9066	No	1.4985	1.933
KH2012:KH.C7.157	Emc	-0.5057	-5.0708	No	No PWM match	No PWM match
KH2012:KH.C3.773	Ci-mT	-0.5064	-13.0630	No	No PWM match	No PWM match
KH2012:KH.L39.1	FoxH-b	-0.6186	-14.7250	No	5.573	9.3465
KH2012:KH.C1.99	SoxB1	-0.6284	-16.0921	No	No PWM match	No PWM match
KH2012:KH.C11.485	Irx-B	-0.7314	-21.9981	No	No PWM Hits	No PWM Hits
KH2012:KH.C4.341	FoxB	-0.9117	-33.3546	No	6.586	13.553

JASPAR 2020 PWM cluster	Max Z-score in cluster	Max Z-score in cluster
NFIC_var_2_	15.44	NFIC::TLX1
RARA_var_2_	15.17	RARA::RXRG

Primary Notochord

KH2012 gene model	TF common name	Log2FC Sibling Cluster	-Log(pvalue) Sibling Cluster	Log2FC Parent Cluster	-Log(pvalue) Parent Cluster	DE in Imai	Matched PWM z-score	PWM Cluster z-score
KH2012:KH.S1404.1	Brachyury	2.4695	112.9035	1.3058	36.1786	Yes	-2.242	4.3455
KH2012:KH.C8.247	ELK	2.2307	95.2289	1.4766	49.3924	No	14.261	29.627
KH2012:KH.L128.12	Mnx	1.7221	102.5684	0.3135	4.3588	Yes	0.0924999999999998	10.3615
KH2012:KH.C11.313	FoxA-a	1.4754	85.9782	0.6386	16.8814	Yes	No PWM Hits	No PWM Hits
KH2012:KH.S215.4	Lhx3	0.8850	57.2897	-0.4747	-9.3575	No	-3.803	10.3615
KH2012:KH.C3.312	E(spl)/hairy-b	0.6642	21.2020	0.6319	12.9863	No	No PWM Hits	No PWM Hits
KH2012:KH.C4.182	Zinc Finger (C3H)	0.6258	26.9408	0.3224	7.4335	No	No PWM match	No PWM match
KH2012:KH.C3.230	Smad6/7	0.5061	14.2005	0.7370	17.3326	No	No PWM match	No PWM match
KH2012:KH.C1.159	E(spl)/hairy-a	0.4984	25.9594	0.3513	6.8140	No	No PWM Hits	No PWM Hits
KH2012:KH.C4.366	ERF2	0.3043	5.9435	0.2726	4.3202	Yes	12.1735	29.627
KH2012:KH.C1.661	MAD	0.2798	6.2502	0.0896	0.7185	No	No PWM match	No PWM match
KH2012:KH.L13.24	FoxN2/3	0.2694	6.2397	0.2030	3.4221	No	-1.5075	9.192
KH2012:KH.S2060.1	Zinc Finger-like2	0.2523	7.6897	0.2265	5.0866	No	-2.6705	-2.6705
KH2012:KH.C7.523	SoxC	-0.2549	-5.6112	-0.0038	-0.4806	Yes	No PWM match	No PWM match
KH2012:KH.C3.348	E12/E47	-0.2719	-8.4693	0.1151	1.3747	Yes	No PWM Hits	No PWM Hits
KH2012:KH.C14.520	Orphan Fox-1	-0.3253	-18.8477	0.0637	0.7154	Yes	No PWM Hits	No PWM Hits
KH2012:KH.C11.485	Irx-B	-0.3794	-15.5766	-0.2565	-7.6931	No	No PWM Hits	No PWM Hits
KH2012:KH.L39.1	FoxH-b	-0.4194	-10.3451	0.0530	0.5688	No	2.4285	6.411
KH2012:KH.S816.1	ZicL	-0.5235	-18.0603	-0.5091	-9.5277	No	5.271	5.271
KH2012:KH.C3.773	Ci-mT	-0.6283	-18.0447	-0.7168	-21.3715	No	No PWM match	No PWM match
KH2012:KH.C7.157	Emc	-0.6329	-6.3916	0.2752	1.4511	No	No PWM match	No PWM match
KH2012:KH.C6.129	Neurogenin	-0.8310	-20.3951	0.1035	3.3601	No	No PWM Hits	No PWM Hits
KH2012:KH.C4.341	FoxB	-0.9344	-52.4219	-0.0051	-0.3422	Yes	0.7585	9.192
KH2012:KH.C1.99	SoxB1	-1.0691	-53.3296	-0.2192	-2.0485	Yes	No PWM match	No PWM match

JASPAR 2020 PWM cluster	Max Z-score in cluster	Max Z-score in cluster
ELK3	29.63	ELF2
PBX3	29.27	PBX3
Arid5a	20.50	Arid5a

Primary Notochord

KH2012 gene model	TF common name	Log2FC Sibling Cluster	-Log(pvalue) Sibling Cluster	Log2FC Parent Cluster	-Log(pvalue) Parent Cluster	DE in Imai	Matched PWM z-score	PWM Cluster z-score
KH2012:KH.S1404.1	Brachyury	2.9610	171.9572	0.5078	14.9623	Not Analyzed	15.8735	15.8735
KH2012:KH.C7.269	Orphan bHLH1	1.2880	52.6089	1.9511	64.2676	Not Analyzed	No PWM match	No PWM match
KH2012:KH.C11.313	FoxA-a	1.0888	55.9461	-0.1602	-5.5904	Not Analyzed	No PWM Hits	No PWM Hits
KH2012:KH.L108.4	CREB/ATF-a	0.5395	23.8849	0.1123	1.1773	Not Analyzed	1.4015	8.15
KH2012:KH.L4.17	Zinc Finger (C2H2)-18	0.4672	19.8291	1.2398	62.3631	Not Analyzed	2.751	2.751
KH2012:KH.C8.247	ELK	0.4607	17.3814	-1.1149	-53.3436	Not Analyzed	8.622	18.5765
KH2012:KH.C4.182	Zinc Finger (C3H)	0.4511	12.0529	0.0298	0.7231	Not Analyzed	No PWM match	No PWM match
KH2012:KH.L13.24	FoxN2/3	0.3865	18.0120	0.2795	7.4762	Not Analyzed	2.428	8.7795
KH2012:KH.S164.12	SoxB2	-0.2646	-13.7427	0.0197	0.1017	Not Analyzed	-4.496	3.589
KH2012:KH.C7.702	Zinc Finger (BTB/POZ)	-0.2824	-7.8900	-0.7178	-33.4399	Not Analyzed	No PWM Hits	No PWM Hits
KH2012:KH.C3.348	E12/E47	-0.2861	-11.3413	0.2198	4.9346	Not Analyzed	No PWM Hits	No PWM Hits
KH2012:KH.L9.33	E(Y)2	-0.2898	-9.2211	-0.2785	-8.1982	Not Analyzed	No PWM match	No PWM match
KH2012:KH.L96.11	Groucho2	-0.3165	-6.6847	-0.4041	-10.6757	Not Analyzed	No PWM match	No PWM match
KH2012:KH.C2.573	Smad1/5	-0.3187	-10.3905	-0.2122	-3.9522	Not Analyzed	No PWM Hits	No PWM Hits
KH2012:KH.C6.117	Smad4	-0.3198	-9.1595	-0.1928	-4.2086	Not Analyzed	2.2695	2.2695
KH2012:KH.C14.178	HMG1/2	-0.4249	-32.6316	0.0064	0.2225	Not Analyzed	No PWM match	No PWM match
KH2012:KH.C7.523	SoxC	-0.4534	-16.1866	-0.3289	-12.5984	Not Analyzed	No PWM match	No PWM match
KH2012:KH.C14.278	Zinc Finger (C2H2)-34	-0.4624	-15.8425	-1.3224	-67.3633	Not Analyzed	No PWM Hits	No PWM Hits
KH2012:KH.C11.485	Irx-B	-0.6382	-34.1108	-0.0209	-2.1568	Not Analyzed	No PWM Hits	No PWM Hits
KH2012:KH.C14.307	MyoD	-0.6465	-9.7983	0.0229	1.1784	Not Analyzed	No PWM Hits	No PWM Hits
KH2012:KH.S654.3	Tbx6b/c/d	-0.6873	-12.3534	-0.0062	-0.2723	Not Analyzed	No PWM match	No PWM match
KH2012:KH.C14.520	Orphan Fox-1	-0.7217	-56.4074	-0.1658	-6.6926	Not Analyzed	No PWM Hits	No PWM Hits
KH2012:KH.C1.274	MyTF	-0.7545	-23.8867	-0.0634	-1.5425	Not Analyzed	-3.222	1.935
KH2012:KH.C14.408	Cdx	-0.7803	-27.3591	0.0143	0.2899	Not Analyzed	No PWM Hits	No PWM Hits
KH2012:KH.S816.1	ZicL	-0.8444	-46.9600	-0.8052	-41.9163	Not Analyzed	-0.445	1.935
KH2012:KH.C4.84	Otx	-0.9669	-35.2924	-0.0864	-3.9035	Not Analyzed	-4.193	3.02
KH2012:KH.C10.113	ets/pointed2	-0.9681	-55.6000	-0.3623	-11.1016	Not Analyzed	11.991	18.5765
KH2012:KH.C3.751	Snail	-1.0169	-11.7681	-0.3202	-10.7082	Not Analyzed	No PWM Hits	No PWM Hits
KH2012:KH.C1.99	SoxB1	-1.1332	-66.2337	0.0519	0.1153	Not Analyzed	No PWM match	No PWM match
KH2012:KH.C6.129	Neurogenin	-1.1780	-22.3867	-0.0774	-2.6375	Not Analyzed	No PWM Hits	No PWM Hits
KH2012:KH.C1.777	Zinc Finger (C2H2)-33	-1.6861	-60.5867	-0.0672	-5.6081	Not Analyzed	No PWM Hits	No PWM Hits

JASPAR 2020 PWM cluster	Max Z-score in cluster	Max Z-score in cluster
ELK3	18.58	ETV2
TBX19	15.87	TBXT

A9.14 & A9.16

KH2012 gene model	TF common name	Log2FC Sibling Cluster	-Log(pvalue) Sibling Cluster	Log2FC Parent Cluster	-Log(pvalue) Parent Cluster	DE in Imai	Matched PWM z-score	PWM Cluster z-score
KH2012:KH.C7.157	Emc	0.8612	6.6870	1.9990	33.0615	No	No PWM match	No PWM match
KH2012:KH.C7.269	Orphan bHLH1	0.6475	6.6512	1.3351	30.6414	No	No PWM match	No PWM match
KH2012:KH.S215.9	Pbx	0.4505	10.0920	0.1178	1.7566	No	0.0695000000000001	14.9065
KH2012:KH.C6.22	Smad2/3a	0.4268	9.1694	-0.1183	-0.8926	No	No PWM match	No PWM match
KH2012:KH.L153.85	Zinc Finger (C2H2)-8	0.3835	8.1034	-0.1154	-0.8685	No	3.276	3.276
KH2012:KH.C14.520	Orphan Fox-1	0.3799	11.3766	0.5650	24.0645	No	No PWM Hits	No PWM Hits
KH2012:KH.L153.110	Zinc Finger (C2H2)-7	0.3608	8.1569	-0.1034	-0.6198	No	No PWM Hits	No PWM Hits
KH2012:KH.C2.290	GABPα	0.3466	6.5169	0.2979	4.6407	No	-8.5805	10.295
KH2012:KH.C3.330	CAGF9	0.3450	8.1015	-0.3341	-5.6331	No	No PWM match	No PWM match
KH2012:KH.C8.920	TR2/4	0.3262	5.0542	-0.0533	-0.7547	No	8.9785	8.9785
KH2012:KH.C3.402	IRF-like7	0.3082	5.4299	0.0862	0.5645	No	No PWM match	No PWM match
KH2012:KH.C10.113	ets/pointed2	0.3036	5.0167	0.5249	13.1270	No	-11.484	10.295
KH2012:KH.C14.306	IRF-like 2	0.2970	6.2300	-0.0411	-0.0882	No	No PWM match	No PWM match
KH2012:KH.C3.59	Zinc finger protein-like 1	0.2918	3.0104	-0.0952	-1.7662	No	No PWM match	No PWM match
KH2012:KH.S406.15	Serum response factor	0.2762	3.9258	0.1281	1.0527	No	6.54	6.54
KH2012:KH.C2.822	E2F1/2/3/6	0.2719	3.6691	-0.0263	-0.0926	No	No PWM match	No PWM match
KH2012:KH.C1.26	CREB/ATF-c	0.2687	4.6064	0.1233	1.3986	No	No PWM Hits	No PWM Hits
KH2012:KH.C2.183	male-specific lethal 3-like	0.2669	4.6000	0.0593	0.4440	No	No PWM match	No PWM match
KH2012:KH.L39.1	FoxH-b	-0.2580	-5.0233	-0.0835	-0.5978	No	3.5335	5.7695
KH2012:KH.C4.509	Zinc Finger (absent, small, or homeotic discs 1)	-0.2614	-3.2511	-0.1496	-1.2195	No	No PWM match	No PWM match
KH2012:KH.C7.782	POU-like	-0.2843	-3.9438	-0.1278	-1.5500	No	No PWM match	No PWM match
KH2012:KH.L13.24	FoxN2/3	-0.3184	-4.6153	-0.0431	-0.4519	No	-6.0925	13.089
KH2012:KH.C11.485	Irx-B	-0.3803	-3.1453	-0.0750	-2.0999	No	No PWM Hits	No PWM Hits
KH2012:KH.C3.312	E(spl)/hairy-b	-0.4044	-3.1928	0.6912	10.3562	No	No PWM Hits	No PWM Hits
KH2012:KH.C1.777	Zinc Finger (C2H2)-33	-0.4530	-5.1063	1.8796	47.4876	No	No PWM Hits	No PWM Hits
KH2012:KH.C9.580	RAR	-0.4780	-7.8131	0.1167	0.9849	No	-6.319	-6.319
KH2012:KH.C14.408	Cdx	-0.8915	-15.0772	-0.0127	-0.8027	No	No PWM Hits	No PWM Hits
KH2012:KH.C11.313	FoxA-a	-1.1125	-16.0139	-0.1970	-4.2344	No	No PWM Hits	No PWM Hits
KH2012:KH.C1.274	MyTF	-1.1800	-18.4457	0.2236	4.4443	No	-8.248	11.3055
KH2012:KH.C8.247	ELK	-1.5779	-27.7879	-0.2912	-8.1823	No	-12.1035	10.295
KH2012:KH.L128.12	Mnx	-2.0181	-30.1107	0.0254	0.6647	No	0.129	12.7225

JASPAR 2020 PWM cluster	Max Z-score in cluster	Max Z-score in cluster
CUX2	26.31	ONECUT1
Hmx1	25.52	Hmx3
Rarg_var_2	20.69	Nr1h3::Rxra
POU4F1	20.55	HNF1B
GATA2	20.07	GATA1
ZNF384	18.71	ZNF384
HOXD10	18.51	HOXB13
FOS::JUNB	18.35	Nfe2l2
POU3F1	17.08	POU5F1
PHOX2A	15.59	PROP1

A8.7 & A8.8

KH2012 gene model	TF common name	Log2FC Sibling Cluster	-Log(pvalue) Sibling Cluster	Log2FC Parent Cluster	-Log(pvalue) Parent Cluster	DE in Imai	Matched PWM z-score	PWM Cluster z-score
KH2012:KH.C6.22	Smad2/3a	0.2902	3.3866	0.0513	0.2712	No	No PWM match	No PWM match
KH2012:KH.C3.877	Zinc Finger (C2H2)-1	-0.3179	-4.8215	0.3503	8.1312	No	No PWM Hits	No PWM Hits
KH2012:KH.C8.247	ELK	-0.5004	-6.3842	0.3803	7.4165	No	-4.107	10.599
KH2012:KH.C3.230	Smad6/7	-0.6718	-21.4146	-0.0600	-2.0223	No	No PWM match	No PWM match
KH2012:KH.C7.157	Emc	-0.9478	-13.2230	0.0152	0.7171	No	No PWM match	No PWM match
KH2012:KH.C6.129	Neurogenin	-1.2186	-21.1776	0.3737	14.9347	No	No PWM Hits	No PWM Hits
KH2012:KH.C3.751	Snail	-1.7344	-53.6571	0.3576	12.6373	No	No PWM Hits	No PWM Hits

JASPAR 2020 PWM cluster	Max Z-score in cluster	Max Z-score in cluster
Hmx1	20.52	Hmx2
Nr2f6	16.10	THRB

A9.13 & A9.15

KH2012 gene model	TF common name	Log2FC Sibling Cluster	-Log(pvalue) Sibling Cluster	Log2FC Parent Cluster	-Log(pvalue) Parent Cluster	DE in Imai	Matched PWM z-score	PWM Cluster z-score
KH2012:KH.L128.12	Mnx	2.0181	30.1107	2.0435	46.1799	Yes	1.1725	7.716
KH2012:KH.C8.247	ELK	1.5779	27.7879	1.2867	22.9762	No	8.899	11.71
KH2012:KH.C1.274	MyTF	1.1800	18.4457	1.4035	38.0925	No	11.375	11.375
KH2012:KH.C11.313	FoxA-a	1.1125	16.0139	0.9154	11.7281	No	No PWM Hits	No PWM Hits
KH2012:KH.C14.408	Cdx	0.8915	15.0772	0.8788	21.8043	No	No PWM Hits	No PWM Hits
KH2012:KH.C9.580	RAR	0.4780	7.8131	0.5946	14.4350	No	3.75	9.441
KH2012:KH.C1.777	Zinc Finger (C2H2)-33	0.4530	5.1063	2.3326	62.6833	No	No PWM Hits	No PWM Hits
KH2012:KH.C3.312	E(spl)/hairy-b	0.4044	3.1928	1.0956	19.5953	No	No PWM Hits	No PWM Hits
KH2012:KH.C11.485	Irx-B	0.3803	3.1453	0.3052	1.1886	No	No PWM Hits	No PWM Hits
KH2012:KH.L13.24	FoxN2/3	0.3184	4.6153	0.2753	4.3193	No	4.008	9.8555
KH2012:KH.C7.782	POU-like	0.2843	3.9438	0.1565	1.8321	No	No PWM match	No PWM match
KH2012:KH.C4.509	Zinc Finger (absent, small, or homeotic discs 1)	0.2614	3.2511	0.1119	1.4624	No	No PWM match	No PWM match
KH2012:KH.L39.1	FoxH-b	0.2580	5.0233	0.1745	3.9071	No	-2.6695	6.926
KH2012:KH.C2.183	male-specific lethal 3-like	-0.2669	-4.6000	-0.2076	-3.9183	No	No PWM match	No PWM match
KH2012:KH.C1.26	CREB/ATF-c	-0.2687	-4.6064	-0.1454	-2.6860	No	No PWM Hits	No PWM Hits
KH2012:KH.C2.822	E2F1/2/3/6	-0.2719	-3.6691	-0.2983	-4.4559	No	No PWM match	No PWM match
KH2012:KH.S406.15	Serum response factor	-0.2762	-3.9258	-0.1482	-2.6171	No	-3.105	-3.105
KH2012:KH.C3.59	Zinc finger protein-like 1	-0.2918	-3.0104	-0.3870	-8.2242	No	No PWM match	No PWM match
KH2012:KH.C14.306	IRF-like 2	-0.2970	-6.2300	-0.3381	-7.2865	No	No PWM match	No PWM match
KH2012:KH.C10.113	ets/pointed2	-0.3036	-5.0167	0.2213	2.0285	No	8.013	11.71
KH2012:KH.C3.402	IRF-like7	-0.3082	-5.4299	-0.2220	-4.8214	No	No PWM match	No PWM match
KH2012:KH.C8.920	TR2/4	-0.3262	-5.0542	-0.3795	-8.5415	No	-3.783	6.105
KH2012:KH.C3.330	CAGF9	-0.3450	-8.1015	-0.6791	-19.3797	No	No PWM match	No PWM match
KH2012:KH.C2.290	GABPα	-0.3466	-6.5169	-0.0487	-1.7307	No	5.678	11.71
KH2012:KH.L153.110	Zinc Finger (C2H2)-7	-0.3608	-8.1569	-0.4642	-11.3683	No	No PWM Hits	No PWM Hits
KH2012:KH.C14.520	Orphan Fox-1	-0.3799	-11.3766	0.1851	3.9270	No	No PWM Hits	No PWM Hits
KH2012:KH.L153.85	Zinc Finger (C2H2)-8	-0.3835	-8.1034	-0.4989	-11.8182	No	-1.644	6.2845
KH2012:KH.C6.22	Smad2/3a	-0.4268	-9.1694	-0.5450	-13.6704	No	No PWM match	No PWM match
KH2012:KH.S215.9	Pbx	-0.4505	-10.0920	-0.3327	-6.6215	No	-0.2725	-0.2725
KH2012:KH.C7.269	Orphan bHLH1	-0.6475	-6.6512	0.6876	11.2746	No	No PWM match	No PWM match
KH2012:KH.C7.157	Emc	-0.8612	-6.6870	1.1378	23.6235	No	No PWM match	No PWM match

JASPAR 2020 PWM cluster	Max Z-score in cluster	Max Z-score in cluster
TFAP2B	23.00	TFAP2A
FOSL2::JUNB_var_2_	17.16	ATF7

A7.4 & A7.8

KH2012 gene model	TF common name	Log2FC Sibling Cluster	-Log(pvalue) Sibling Cluster	DE in Imai	Matched PWM z-score	PWM Cluster z-score
KH2012:KH.C4.341	FoxB	0.9117	33.3546	Yes	-5.8835	0.85
KH2012:KH.C11.485	Irx-B	0.7314	21.9981	Yes	No PWM Hits	No PWM Hits
KH2012:KH.C1.99	SoxB1	0.6284	16.0921	No	No PWM match	No PWM match
KH2012:KH.L39.1	FoxH-b	0.6186	14.7250	No	-5.7865	2.9485
KH2012:KH.C3.773	Ci-mT	0.5064	13.0630	No	No PWM match	No PWM match
KH2012:KH.C7.157	Emc	0.5057	5.0708	No	No PWM match	No PWM match
KH2012:KH.C9.892	RXR	0.3690	9.9066	No	-1.5395	4.8995
KH2012:KH.L153.110	Zinc Finger (C2H2)-7	0.3145	7.2689	No	No PWM Hits	No PWM Hits
KH2012:KH.S899.4	BTF3	0.3064	8.5329	No	No PWM match	No PWM match
KH2012:KH.C5.50	Zinc Finger (C2H2)-28	0.2998	6.9499	No	No PWM match	No PWM match
KH2012:KH.C2.290	GABPα	0.2966	7.3008	No	-8.8415	1.1845
KH2012:KH.C11.735	Zinc Finger (C2H2)-29	0.2852	5.9628	No	1.7665	1.7665
KH2012:KH.C14.105	CNOT7/8	0.2607	3.4516	No	No PWM match	No PWM match
KH2012:KH.C3.103	RBP1-like	-0.2918	-8.5341	No	No PWM match	No PWM match
KH2012:KH.C12.116	Pax258-B	-0.3029	-6.3070	No	No PWM Hits	No PWM Hits
KH2012:KH.C14.449	CBP	-0.3223	-6.8396	No	No PWM match	No PWM match
KH2012:KH.C3.312	E(spl)/hairy-b	-0.3372	-8.0416	No	No PWM Hits	No PWM Hits
KH2012:KH.C4.182	Zinc Finger (C3H)	-0.3581	-4.8832	No	No PWM match	No PWM match
KH2012:KH.S1272.2	ALR-like	-0.3581	-4.9484	No	No PWM match	No PWM match
KH2012:KH.C4.509	Zinc Finger (absent, small, or homeotic discs 1)	-0.4979	-9.5414	No	No PWM match	No PWM match
KH2012:KH.C11.313	FoxA-a	-0.5575	-7.3507	No	No PWM Hits	No PWM Hits
KH2012:KH.S1404.1	Brachyury	-1.1711	-70.8956	No	-1.7935	-1.7935
KH2012:KH.C8.247	ELK	-1.3201	-51.9200	No	-4.9525	1.1845
KH2012:KH.S215.4	Lhx3	-1.3648	-71.8561	No	-4.3015	2.3335
KH2012:KH.L128.12	Mnx	-1.4244	-77.3644	No	-4.9395	2.3335

JASPAR 2020 PWM cluster	Max Z-score in cluster	Max Z-score in cluster
FOS::JUNB	22.89	MAF::NFE2
BHLHA15_var_2_	19.40	MXI1
OVOL2	17.82	MYBL2
ARNT2	17.23	MAX::MYC

A9.29

KH2012 gene model	TF common name	Log2FC Sibling Cluster	-Log(pvalue) Sibling Cluster	Log2FC Parent Cluster	-Log(pvalue) Parent Cluster	DE in Imai	Matched PWM z-score	PWM Cluster z-score
KH2012:KH.C8.247	ELK	1.6720	13.4005	0.7398	6.0802	No	-6.0155	12.36
KH2012:KH.C14.408	Cdx	1.2895	12.7017	1.4739	30.6292	No	No PWM Hits	No PWM Hits
KH2012:KH.C1.274	MyTF	1.1964	11.3820	1.2964	26.1843	No	No PWM Hits	11.875
KH2012:KH.S816.1	ZicL	0.6955	5.2135	0.0434	0.2187	Yes	4.815	11.875
KH2012:KH.C3.312	E(spl)/hairy-b	0.6239	5.7069	0.9104	12.8936	No	No PWM Hits	No PWM Hits
KH2012:KH.C11.535	Zinc Finger (C2H2)-17	0.6036	4.5034	0.2906	1.9703	No	No PWM match	No PWM match
KH2012:KH.L37.41	TCFL1	0.4874	3.2997	-0.1216	-0.4793	No	No PWM match	No PWM match
KH2012:KH.L9.33	E(Y)2	0.4456	3.1671	-0.1752	-0.8797	No	No PWM match	No PWM match
KH2012:KH.C2.161	FXR	0.4098	3.1322	0.2152	1.0682	No	No PWM Hits	No PWM Hits
KH2012:KH.C1.573	p53/p73-a	0.2895	3.0887	0.1610	1.6245	No	No PWM match	No PWM match
KH2012:KH.C7.111	Transcription initiation protein SPT4 homolog	0.2604	3.2655	-0.0425	-0.0867	No	No PWM match	No PWM match
KH2012:KH.C7.782	POU-like	-0.3695	-3.9267	-0.2491	-2.6139	No	No PWM match	No PWM match
KH2012:KH.C9.68	Pax6	-0.5585	-3.4277	0.1520	4.3583	No	1.1715	1.1715
KH2012:KH.C7.157	Emc	-1.1936	-8.8059	-0.6647	-3.1013	No	No PWM match	No PWM match

JASPAR 2020 PWM cluster	Max Z-score in cluster	Max Z-score in cluster
RFX2	35.84	RFX1
POU4F1	25.79	POU4F2
NKX2-3	24.44	NKX2-2
MEIS1_var_2_	24.36	PBX2
Esrrg	23.93	ESRRA
ARNT2	22.61	MYCN
GATA2	22.57	Mecom
BHLHA15_var_2_	21.55	OLIG3
PDX1	21.30	NFYB
FOS::JUNB	20.64	MAFK
RARA_var_2	18.13	RARA::RXRA
FOSL2::JUNB_var_2_	17.36	ATF2
SP3	16.66	EGR1
ESR1	15.13	ESR2
INSM1	15.01	INSM1

A9.30

KH2012 gene model	TF common name	Log2FC Sibling Cluster	-Log(pvalue) Sibling Cluster	Log2FC Parent Cluster	-Log(pvalue) Parent Cluster	DE in Imai	Matched PWM z-score	PWM Cluster z-score
KH2012:KH.C7.157	Emc	1.1936	8.8059	0.5289	3.9698	No	No PWM match	No PWM match
KH2012:KH.C9.68	Pax6	0.5585	3.4277	0.7105	14.5667	Yes	-0.5495	4.7395
KH2012:KH.C7.782	POU-like	0.3695	3.9267	0.1204	1.0958	No	No PWM match	No PWM match
KH2012:KH.C7.111	Transcription initiation protein SPT4 homolog	-0.2604	-3.2655	-0.3028	-2.7663	No	No PWM match	No PWM match
KH2012:KH.C1.573	p53/p73-a	-0.2895	-3.0887	-0.1285	-1.4587	No	No PWM match	No PWM match
KH2012:KH.C2.161	FXR	-0.4098	-3.1322	-0.1946	-2.0299	No	No PWM Hits	No PWM Hits
KH2012:KH.L9.33	E(Y)2	-0.4456	-3.1671	-0.6208	-6.5790	No	No PWM match	No PWM match
KH2012:KH.L37.41	TCFL1	-0.4874	-3.2997	-0.6090	-6.3767	No	No PWM match	No PWM match
KH2012:KH.C11.535	Zinc Finger (C2H2)-17	-0.6036	-4.5034	-0.3130	-2.4915	No	No PWM match	No PWM match
KH2012:KH.C3.312	E(spl)/hairy-b	-0.6239	-5.7069	0.2865	1.6850	No	No PWM Hits	No PWM Hits
KH2012:KH.S816.1	ZicL	-0.6955	-5.2135	-0.6521	-7.5768	No	-4.007	5.516
KH2012:KH.C1.274	MyTF	-1.1964	-11.3820	0.1000	1.9806	No	-3.103	5.516
KH2012:KH.C14.408	Cdx	-1.2895	-12.7017	0.1844	2.1445	No	No PWM Hits	No PWM Hits
KH2012:KH.C8.247	ELK	-1.6720	-13.4005	-0.9322	-7.9501	No	3.067	17.316

JASPAR 2020 PWM cluster	Max Z-score in cluster	Max Z-score in cluster
ELK3	17.32	SPIB

A8.15 & A8.16

KH2012 gene model	TF common name	Log2FC Sibling Cluster	-Log(pvalue) Sibling Cluster	Log2FC Parent Cluster	-Log(pvalue) Parent Cluster	DE in Imai	Matched PWM z-score	PWM Cluster z-score
KH2012:KH.C3.751	Snail	1.7344	53.6571	2.0920	95.4680	Yes	No PWM Hits	No PWM Hits
KH2012:KH.C6.129	Neurogenin	1.2186	21.1776	1.5923	58.7156	Yes	No PWM Hits	No PWM Hits
KH2012:KH.C7.157	Emc	0.9478	13.2230	0.9631	13.4789	No	No PWM match	No PWM match
KH2012:KH.C3.230	Smad6/7	0.6718	21.4146	0.6118	18.7326	No	No PWM match	No PWM match
KH2012:KH.C8.247	ELK	0.5004	6.3842	0.8806	19.7373	No	8.266	9.857
KH2012:KH.C3.877	Zinc Finger (C2H2)-1	0.3179	4.8215	0.6682	17.9085	No	No PWM Hits	No PWM Hits
KH2012:KH.C6.22	Smad2/3a	-0.2902	-3.3866	-0.2388	-3.2093	No	No PWM match	No PWM match

JASPAR 2020 PWM cluster	Max Z-score in cluster	Max Z-score in cluster
SP3	37.74	KLF15
PROX1	32.06	PROX1
Rarg_var_2_	26.49	Nr1h3::Rxra
NKX2-3	23.08	NKX2-5
Zic1::Zic2	23.04	ZKSCAN1
MAFG	22.59	Mafb
BHLHA15_var_2	20.93	FERD3L
ARNT2	19.40	MYC
PITX3	15.87	Dmbx1
INSM1	15.50	CTCFL

A9.31

KH2012 gene model	TF common name	Log2FC Sibling Cluster	-Log(pvalue) Sibling Cluster	Log2FC Parent Cluster	-Log(pvalue) Parent Cluster	DE in Imai	Matched PWM z-score	PWM Cluster z-score
KH2012:KH.S654.3	Tbx6b/c/d	2.1312	20.2251	2.5146	41.7932	Not Analyzed	No PWM match	No PWM match
KH2012:KH.C14.307	MyoD	2.1039	20.1438	2.3877	39.1631	Not Analyzed	No PWM Hits	No PWM Hits
KH2012:KH.C14.408	Cdx	1.6740	16.0234	1.8509	34.3134	Not Analyzed	No PWM Hits	No PWM Hits
KH2012:KH.C8.247	ELK	1.4299	13.4251	1.2701	17.8942	Not Analyzed	-6.997	12.3405
KH2012:KH.C1.274	MyTF	1.0000	10.7034	0.9745	19.5798	Not Analyzed	No PWM Hits	10.249
KH2012:KH.C4.366	ERF2	0.7776	6.9223	0.8845	13.1796	Not Analyzed	-2.0255	12.3405
KH2012:KH.S423.6	SMYD1	0.7162	5.0146	0.7776	12.9427	Not Analyzed	No PWM match	No PWM match
KH2012:KH.C3.137	ATF4/5	0.5959	6.4391	0.2756	2.2770	Not Analyzed	No PWM match	No PWM match
KH2012:KH.C5.213	ARNT	0.5907	6.8882	-0.0388	-0.1549	Not Analyzed	-8.5455	28.934
KH2012:KH.C4.83	HIF	0.5642	4.0566	0.3798	2.7404	Not Analyzed	No PWM match	No PWM match
KH2012:KH.C2.290	GABPα	0.5527	4.7041	0.0893	0.0466	Not Analyzed	7.6315	12.3405
KH2012:KH.S1482.3	NFRKB	0.5372	5.1619	0.0555	0.3149	Not Analyzed	No PWM match	No PWM match
KH2012:KH.C1.27	LAG1-like1	0.5291	4.9959	-0.1172	-0.3742	Not Analyzed	No PWM match	No PWM match
KH2012:KH.C11.535	Zinc Finger (C2H2)-17	0.5236	3.8308	0.3964	2.7435	Not Analyzed	No PWM match	No PWM match
KH2012:KH.C14.306	IRF-like 2	0.5047	6.9340	-0.0498	-0.1473	Not Analyzed	No PWM match	No PWM match
KH2012:KH.S215.9	Pbx	0.4956	4.8542	-0.0734	-0.0796	Not Analyzed	4.2335	34.3445
KH2012:KH.C11.735	Zinc Finger (C2H2)-29	0.4849	5.4538	0.0973	1.2223	Not Analyzed	8.7705	8.7705
KH2012:KH.C2.687	VDR-a	0.4532	3.2944	0.2176	0.4549	Not Analyzed	No PWM match	No PWM match
KH2012:KH.L96.52	transcription factor4	0.4375	3.8639	-0.2088	-1.0636	Not Analyzed	0.9965	28.934
KH2012:KH.C14.520	Orphan Fox-1	0.4227	5.5300	-0.1039	-1.2892	Not Analyzed	No PWM Hits	No PWM Hits
KH2012:KH.C8.162	Zinc Finger (C2H2)-21	0.4128	3.6053	0.1795	1.1551	Not Analyzed	No PWM Hits	No PWM Hits
KH2012:KH.L96.11	Groucho2	0.4030	4.2216	0.6350	10.9810	Not Analyzed	No PWM match	No PWM match
KH2012:KH.C2.161	FXR	0.3792	4.2706	0.1818	1.9396	Not Analyzed	No PWM Hits	No PWM Hits
KH2012:KH.C4.504	FoxM	0.3792	4.2706	-0.3520	-3.7830	Not Analyzed	-13.1385	18.743
KH2012:KH.C3.644	C/EBPα/γ	0.3578	3.9523	0.0566	0.3258	Not Analyzed	No PWM Hits	No PWM Hits
KH2012:KH.C3.84	HNF4	0.3219	4.5030	-0.2106	-2.0235	Not Analyzed	No PWM Hits	No PWM Hits
KH2012:KH.C11.381	prep	0.3203	3.9230	0.0385	0.5901	Not Analyzed	6.454	27.2485
KH2012:KH.C9.717	FoxH-a	0.3189	3.6108	0.5502	14.2990	Not Analyzed	2.214	20.1305
KH2012:KH.S437.3	Zinc Finger (C2H2)-10	0.3177	3.8272	-0.1988	-1.5600	Not Analyzed	-13.5	-13.5
KH2012:KH.C10.604	Zinc Finger (C2H2)-3	0.3012	3.3679	0.0956	0.7127	Not Analyzed	-6.2825	-6.2825
KH2012:KH.C6.22	Smad2/3a	0.2856	3.4349	0.0000	0.0000	Not Analyzed	No PWM match	No PWM match
KH2012:KH.C3.604	LAG1-like3	0.2818	3.3668	0.1009	0.7742	Not Analyzed	No PWM match	No PWM match
KH2012:KH.L7.6	Sex comb on midleg-like1	0.2801	3.9036	-0.0623	-0.5115	Not Analyzed	No PWM match	No PWM match
KH2012:KH.C2.480	LXR	0.2687	3.3387	-0.1988	-1.2477	Not Analyzed	-0.805	20.029
KH2012:KH.C9.580	RAR	-0.4621	-4.3305	0.0385	0.4236	Not Analyzed	-15.342	-15.342
KH2012:KH.L39.1	FoxH-b	-0.4968	-3.9485	-0.2410	-1.2565	Not Analyzed	2.214	20.1305
KH2012:KH.S1272.2	ALR-like	-0.6131	-3.5556	-0.1903	-2.0587	Not Analyzed	No PWM match	No PWM match
KH2012:KH.C6.129	Neurogenin	-0.6766	-3.1422	0.7650	12.1590	Not Analyzed	No PWM Hits	No PWM Hits
KH2012:KH.C7.269	Orphan bHLH1	-0.6859	-5.3597	-0.0255	-0.7025	Not Analyzed	No PWM match	No PWM match
KH2012:KH.C3.751	Snail	-0.8082	-8.0829	0.2298	2.0846	Not Analyzed	No PWM Hits	No PWM Hits
KH2012:KH.L24.10	COE	-1.3800	-10.6023	0.0506	1.8027	Not Analyzed	No PWM Hits	No PWM Hits
KH2012:KH.C7.157	Emc	-3.1388	-21.1473	-1.6741	-14.9891	Not Analyzed	No PWM match	No PWM match

JASPAR 2020 PWM cluster	Max Z-score in cluster	Max Z-score in cluster
Nr2f6	60.41	Nr2f6
HOXD10	40.57	HOXB13
TBX19	39.19	TBXT
OVOL2	36.28	MYBL2
MEIS1_var_2_	34.34	PBX2
NFIL3	30.38	CEBPD
ARNT2	28.93	HES7
POU4F1	27.88	HNF1B
BHLHA15_var_2	27.25	MXI1
Rarg_var_2_	24.31	Nr1h3::Rxra
Rarg	24.19	Rarg
SMAD3	23.53	ZSCAN29
CUX2	23.43	ONECUT1
FOS::JUNB	22.37	Nfe2l2
NFKB2	20.90	NFKB1
SP3	20.22	KLF3
PITX3	20.13	Dmbx1
Esrrg	20.03	NR2F2
ZNF449	19.65	ZNF449
GATA2	18.80	Mecom
Foxf1	18.74	FOXD2
NKX2-3	18.64	MSANTD3
TEAD4	18.01	TEAD2
GRHL2	16.69	SIX2
GCM2	15.77	CENPB
PHOX2A	15.65	PROP1

A9.32

KH2012 gene model	TF common name	Log2FC Sibling Cluster	-Log(pvalue) Sibling Cluster	Log2FC Parent Cluster	-Log(pvalue) Parent Cluster	DE in Imai	Matched PWM z-score	PWM Cluster z-score
KH2012:KH.C7.157	Emc	3.1388	21.1473	1.4648	11.9208	No	No PWM match	No PWM match
KH2012:KH.L24.10	COE	1.3800	10.6023	1.4306	20.5172	Yes	No PWM Hits	No PWM Hits
KH2012:KH.C3.751	Snail	0.8082	8.0829	1.0380	13.5173	No	No PWM Hits	No PWM Hits
KH2012:KH.C7.269	Orphan bHLH1	0.6859	5.3597	0.6604	7.5642	No	No PWM match	No PWM match
KH2012:KH.C6.129	Neurogenin	0.6766	3.1422	1.4416	13.9110	No	No PWM Hits	No PWM Hits
KH2012:KH.S1272.2	ALR-like	0.6131	3.5556	0.4228	1.8030	No	No PWM match	No PWM match
KH2012:KH.L39.1	FoxH-b	0.4968	3.9485	0.2558	2.2567	No	0.026	5.9235
KH2012:KH.C9.580	RAR	0.4621	4.3305	0.5006	5.1439	No	1.5075	4.0405
KH2012:KH.C2.480	LXR	-0.2687	-3.3387	-0.4675	-5.9061	No	0.2825	3.8735
KH2012:KH.L7.6	Sex comb on midleg-like1	-0.2801	-3.9036	-0.3424	-5.0686	No	No PWM match	No PWM match
KH2012:KH.C3.604	LAG1-like3	-0.2818	-3.3668	-0.1809	-2.5109	No	No PWM match	No PWM match
KH2012:KH.C6.22	Smad2/3a	-0.2856	-3.4349	-0.2856	-4.0466	No	No PWM match	No PWM match
KH2012:KH.C10.604	Zinc Finger (C2H2)-3	-0.3012	-3.3679	-0.2056	-2.5977	No	2.1355	2.1355
KH2012:KH.S437.3	Zinc Finger (C2H2)-10	-0.3177	-3.8272	-0.5164	-6.4789	No	15.194	15.194
KH2012:KH.C9.717	FoxH-a	-0.3189	-3.6108	0.2313	3.5854	No	0.026	5.9235
KH2012:KH.C11.381	prep	-0.3203	-3.9230	-0.2818	-3.1291	No	-2.406	10.475
KH2012:KH.C3.84	HNF4	-0.3219	-4.5030	-0.5325	-8.0659	No	No PWM Hits	No PWM Hits
KH2012:KH.C3.644	C/EBPα/γ	-0.3578	-3.9523	-0.3012	-3.7032	No	No PWM Hits	No PWM Hits
KH2012:KH.C2.161	FXR	-0.3792	-4.2706	-0.1973	-2.1390	No	No PWM Hits	No PWM Hits
KH2012:KH.C4.504	FoxM	-0.3792	-4.2706	-0.7312	-10.6356	No	5.4155	8.2065
KH2012:KH.L96.11	Groucho2	-0.4030	-4.2216	0.2320	0.6896	No	No PWM match	No PWM match
KH2012:KH.C8.162	Zinc Finger (C2H2)-21	-0.4128	-3.6053	-0.2333	-2.1755	No	No PWM Hits	No PWM Hits
KH2012:KH.C14.520	Orphan Fox-1	-0.4227	-5.5300	-0.5266	-10.8911	No	No PWM Hits	No PWM Hits
KH2012:KH.L96.52	transcription factor4	-0.4375	-3.8639	-0.6463	-6.2466	No	-0.528	9.634
KH2012:KH.C2.687	VDR-a	-0.4532	-3.2944	-0.2356	-3.2829	No	No PWM match	No PWM match
KH2012:KH.C11.735	Zinc Finger (C2H2)-29	-0.4849	-5.4538	-0.3876	-3.5934	No	-3.0765	-3.0765
KH2012:KH.S215.9	Pbx	-0.4956	-4.8542	-0.5690	-5.1555	No	-0.7695	-0.7695
KH2012:KH.C14.306	IRF-like 2	-0.5047	-6.9340	-0.5545	-6.8209	No	No PWM match	No PWM match
KH2012:KH.C11.535	Zinc Finger (C2H2)-17	-0.5236	-3.8308	-0.1271	-1.1023	No	No PWM match	No PWM match
KH2012:KH.C1.27	LAG1-like1	-0.5291	-4.9959	-0.6463	-5.9756	No	No PWM match	No PWM match
KH2012:KH.S1482.3	NFRKB	-0.5372	-5.1619	-0.4817	-5.5496	No	No PWM match	No PWM match
KH2012:KH.C2.290	GABPα	-0.5527	-4.7041	-0.4634	-5.8139	No	-2.729	4.988
KH2012:KH.C4.83	HIF	-0.5642	-4.0566	-0.1844	-1.5346	No	No PWM match	No PWM match
KH2012:KH.C5.213	ARNT	-0.5907	-6.8882	-0.6295	-6.6945	No	2.462	9.634
KH2012:KH.C3.137	ATF4/5	-0.5959	-6.4391	-0.3203	-3.8255	No	No PWM match	No PWM match
KH2012:KH.S423.6	SMYD1	-0.7162	-5.0146	0.0614	2.0763	No	No PWM match	No PWM match
KH2012:KH.C4.366	ERF2	-0.7776	-6.9223	0.1069	0.7032	No	0.362	4.988
KH2012:KH.C1.274	MyTF	-1.0000	-10.7034	-0.0255	-0.6164	No	No PWM Hits	3.3705
KH2012:KH.C8.247	ELK	-1.4299	-13.4251	-0.1598	-1.1626	No	2.1075	4.988
KH2012:KH.C14.408	Cdx	-1.6740	-16.0234	0.1769	3.5850	No	No PWM Hits	No PWM Hits
KH2012:KH.C14.307	MyoD	-2.1039	-20.1438	0.2838	1.4663	No	No PWM Hits	No PWM Hits
KH2012:KH.S654.3	Tbx6b/c/d	-2.1312	-20.2251	0.3833	3.5850	No	No PWM match	No PWM match

JASPAR 2020 PWM cluster	Max Z-score in cluster	Max Z-score in cluster
ZNF143	15.19	ZNF143

B7.4

KH2012 gene model	TF common name	Log2FC Sibling Cluster	-Log(pvalue) Sibling Cluster	DE in Imai	Matched PWM z-score	PWM Cluster z-score
KH2012:KH.C14.307	MyoD	1.0630	35.5728	Yes	No PWM Hits	No PWM Hits
KH2012:KH.S423.6	SMYD1	0.8093	23.1876	Yes	No PWM match	No PWM match
KH2012:KH.C3.773	Ci-mT	0.6972	10.0735	No	No PWM match	No PWM match
KH2012:KH.C4.341	FoxB	0.4473	15.7021	No	-4.3345	4.1945
KH2012:KH.S654.3	Tbx6b/c/d	0.4281	9.1086	No	No PWM match	No PWM match
KH2012:KH.C14.520	Orphan Fox-1	0.3943	7.6506	No	No PWM Hits	No PWM Hits
KH2012:KH.C5.130	Zinc Finger (C2H2)-27	0.3843	4.3306	No	No PWM Hits	No PWM Hits
KH2012:KH.C1.264	Y-box protein1/2/3	0.2929	3.7661	No	No PWM match	No PWM match
KH2012:KH.C6.22	Smad2/3a	0.2897	3.8543	No	No PWM match	No PWM match
KH2012:KH.S816.1	ZicL	0.2845	3.1093	No	12.3765	12.3765
KH2012:KH.C3.17	ROR	0.2579	3.0918	Yes	-0.5895	17.078
KH2012:KH.C11.481	IRF-like6	-0.2775	-3.1072	No	No PWM match	No PWM match
KH2012:KH.C11.450	CBF	-0.2816	-3.1843	No	No PWM match	No PWM match
KH2012:KH.C1.27	LAG1-like1	-0.3814	-3.4259	No	No PWM match	No PWM match
KH2012:KH.C3.697	Zinc Finger (C2H2)-5	-0.3881	-4.2418	No	No PWM Hits	No PWM Hits
KH2012:KH.C8.247	ELK	-0.3911	-3.4746	No	3.322	18.028
KH2012:KH.S1404.1	Brachyury	-0.3964	-4.5174	No	-5.119	1.468
KH2012:KH.S215.4	Lhx3	-0.5045	-7.9421	No	-1.9595	8.9245
KH2012:KH.L128.12	Mnx	-0.5361	-7.8130	No	0.7885	8.9245
KH2012:KH.C11.485	Irx-B	-0.7235	-10.1545	No	No PWM Hits	No PWM Hits

JASPAR 2020 PWM cluster	Max Z-score in cluster	Max Z-score in cluster
TFAP2B	20.54	TFAP2B
ELK3	18.03	ELF4
Esrrg	17.08	NR2F1
PAX1	17.01	PAX9
Rarg	16.90	Rarg

B7.4 Muscle

KH2012 gene model	TF common name	Log2FC Sibling Cluster	-Log(pvalue) Sibling Cluster	Log2FC Parent Cluster	-Log(pvalue) Parent Cluster	DE in Imai	Matched PWM z-score	PWM Cluster z-score
KH2012:KH.S423.6	SMYD1	2.8034	62.8682	2.6508	26.8647	Yes	No PWM match	No PWM match
KH2012:KH.C14.307	MyoD	1.9502	67.7817	1.0597	17.4201	Yes	No PWM Hits	No PWM Hits
KH2012:KH.S654.3	Tbx6b/c/d	1.8811	54.7737	1.5962	23.9272	Yes	No PWM match	No PWM match
KH2012:KH.C3.773	Ci-mT	1.1579	30.5712	0.3282	3.1279	No	No PWM match	No PWM match
KH2012:KH.C3.751	Snail	0.9989	30.8376	1.1502	21.0271	No	No PWM Hits	No PWM Hits
KH2012:KH.S816.1	ZicL	0.9551	24.4081	0.3344	2.6361	No	29.4925	29.4925
KH2012:KH.C4.341	FoxB	0.8051	29.3442	0.4719	4.0620	No	-5.1785	4.5055
KH2012:KH.C14.377	Otp	0.7323	30.3260	0.7851	12.2852	Yes	No PWM Hits	No PWM Hits
KH2012:KH.C3.17	ROR	0.4578	9.6959	0.2604	2.1912	Yes	4.7285	20.1775
KH2012:KH.C5.90	core-binding factor, β subunit	0.3666	6.8769	-0.0217	-0.0047	No	No PWM match	No PWM match
KH2012:KH.C1.264	Y-box protein1/2/3	0.3566	4.8308	0.1165	0.2620	No	No PWM match	No PWM match
KH2012:KH.C12.370	FoxK	0.3480	4.4778	0.4547	3.6541	No	No PWM Hits	No PWM Hits
KH2012:KH.C2.573	Smad1/5	0.3308	7.0963	-0.0462	-0.1559	No	No PWM Hits	No PWM Hits
KH2012:KH.C3.230	Smad6/7	0.3015	8.2424	0.5975	8.6273	No	No PWM match	No PWM match
KH2012:KH.C10.113	ets/pointed2	0.2915	3.0348	0.4084	3.1900	No	-6.408	0.255
KH2012:KH.L154.23	Trh	0.2902	4.5951	0.4077	3.9270	No	No PWM match	No PWM match
KH2012:KH.C10.367	Six36	0.2884	14.9562	0.2131	2.8001	No	0.87	3.1595
KH2012:KH.C11.535	Zinc Finger (C2H2)-17	0.2855	5.3799	0.1138	0.6951	No	No PWM match	No PWM match
KH2012:KH.C11.238	RelA	0.2790	5.0842	0.1309	0.7167	No	-9.8115	13.8365
KH2012:KH.S1272.2	ALR-like	-0.2640	-4.1735	0.2171	2.2924	No	No PWM match	No PWM match
KH2012:KH.C1.99	SoxB1	-0.2683	-4.6034	0.0000	0.0000	No	No PWM match	No PWM match
KH2012:KH.S215.4	Lhx3	-0.2835	-5.2566	0.0614	1.3222	No	1.2615	8.7175
KH2012:KH.C14.200	ATRX	-0.3099	-4.0270	-0.1343	-1.0276	No	No PWM match	No PWM match
KH2012:KH.L39.1	FoxH-b	-0.4178	-5.9938	-0.1985	-2.9739	No	3.1915	3.1915
KH2012:KH.C4.84	Otx	-0.4494	-3.4867	0.0714	0.2134	No	0.41	3.1915
KH2012:KH.C11.485	Irx-B	-0.5084	-7.2900	0.2578	3.0920	No	No PWM Hits	No PWM Hits
KH2012:KH.C5.416	TWIST-like-1a/b	-0.5363	-6.7531	0.0310	0.8135	No	No PWM match	No PWM match
KH2012:KH.L128.12	Mnx	-0.5790	-9.3220	0.0422	0.5514	No	-3.6665	8.7175
KH2012:KH.C11.313	FoxA-a	-0.7310	-8.7562	-0.2596	-2.4424	No	No PWM Hits	No PWM Hits
KH2012:KH.C7.157	Emc	-0.8678	-14.1890	-0.1420	-3.5710	No	No PWM match	No PWM match
KH2012:KH.S1404.1	Brachyury	-1.0358	-10.5476	-0.0652	-1.1381	No	15.169	15.169

JASPAR 2020 PWM cluster	Max Z-score in cluster	Max Z-score in cluster
NR3C1	41.87	Ar
GLIS2	29.49	ZIC4
ZSCAN4	28.35	ZSCAN4
Nr2f6	25.12	RXRB
BHLHA15_var_2	23.11	TGIF2
Esrrg	20.18	NR2F2
TBX21	19.93	TBX2
E2F7	17.96	E2F4
Stat5a::Stat5b	17.85	BCL6
RFX2	17.07	RFX4
SRF	17.01	SRF
ARNT2	15.99	MYCN
Rhox11	15.24	ZNF341
TBX19	15.17	TBXT

B7.4 Muscle

KH2012 gene model	TF common name	Log2FC Sibling Cluster	-Log(pvalue) Sibling Cluster	Log2FC Parent Cluster	-Log(pvalue) Parent Cluster	DE in Imai	Matched PWM z-score	PWM Cluster z-score
KH2012:KH.S423.6	SMYD1	4.6959	72.4371	1.4250	31.6885	Not Analyzed	No PWM match	No PWM match
KH2012:KH.S654.3	Tbx6b/c/d	2.5178	60.5389	0.3552	5.1287	Not Analyzed	No PWM match	No PWM match
KH2012:KH.C14.377	Otp	2.0273	54.4010	1.2117	20.2431	Not Analyzed	No PWM Hits	No PWM Hits
KH2012:KH.C14.307	MyoD	1.9528	58.1689	-0.2031	-3.2503	Not Analyzed	No PWM Hits	No PWM Hits
KH2012:KH.C3.751	Snail	1.7155	46.7687	0.6622	12.1708	Not Analyzed	No PWM Hits	No PWM Hits
KH2012:KH.L128.12	Mnx	1.5846	42.6783	1.6343	30.5835	Not Analyzed	5.3605	13.92
KH2012:KH.C1.264	Y-box protein1/2/3	0.9285	29.5449	0.9192	20.7759	Not Analyzed	No PWM match	No PWM match
KH2012:KH.C3.17	ROR	0.6989	16.2873	1.0537	22.6633	Not Analyzed	0.0685	12.2915
KH2012:KH.S899.4	BTF3	0.6060	21.1254	0.4254	9.9324	Not Analyzed	No PWM match	No PWM match
KH2012:KH.C4.83	HIF	0.5983	17.7843	0.3049	3.8741	Not Analyzed	No PWM match	No PWM match
KH2012:KH.C11.485	Irx-B	0.5980	9.4290	0.6987	8.3355	Not Analyzed	No PWM Hits	No PWM Hits
KH2012:KH.C9.717	FoxH-a	0.4527	12.0499	0.4711	8.6745	Not Analyzed	-6.179	-1.6025
KH2012:KH.L8.11	Tbx6a	0.3989	10.8082	-0.0715	-0.9983	Not Analyzed	No PWM match	No PWM match
KH2012:KH.C1.661	MAD	0.3981	5.7736	0.6135	7.3133	Not Analyzed	No PWM match	No PWM match
KH2012:KH.S816.1	ZicL	0.3803	7.6023	-1.0144	-24.3547	Not Analyzed	2.949	11.057
KH2012:KH.S406.15	Serum response factor	0.3476	8.8349	-0.0751	-0.7363	Not Analyzed	0.151	0.151
KH2012:KH.L13.24	FoxN2/3	0.3356	8.6929	0.5547	13.7308	Not Analyzed	-4.44	-1.4485
KH2012:KH.C10.113	ets/pointed2	0.3335	4.4732	-0.0873	-0.7840	Not Analyzed	-16.8915	0.417
KH2012:KH.C14.306	IRF-like 2	0.3320	5.1510	0.1159	0.4346	Not Analyzed	No PWM match	No PWM match
KH2012:KH.L96.52	transcription factor4	0.3181	6.3979	-0.3145	-4.2608	Not Analyzed	6.484	7.244
KH2012:KH.C3.165	Zinc Finger (C2H2)-26	0.2882	5.9395	-0.0790	-0.3316	Not Analyzed	No PWM Hits	No PWM Hits
KH2012:KH.C1.685	Prd-B	0.2856	8.5763	-0.2098	-2.7971	Not Analyzed	No PWM match	No PWM match
KH2012:KH.C3.713	p53/p73-b	-0.2544	-3.2738	-0.4917	-9.0483	Not Analyzed	No PWM match	No PWM match
KH2012:KH.C14.586	Zinc Finger (BTB/C2H2)	-0.2654	-4.1174	-0.2763	-4.5591	Not Analyzed	-1.1015	-1.1015
KH2012:KH.C1.99	SoxB1	-0.2838	-8.6761	0.2122	5.4199	Not Analyzed	No PWM match	No PWM match
KH2012:KH.L41.71	Zinc Finger (C2H2)-32	-0.3079	-4.7522	-0.8055	-22.9024	Not Analyzed	No PWM match	No PWM match
KH2012:KH.C7.207	FoxP	-0.3162	-7.4551	-0.0781	-2.0140	Not Analyzed	No PWM Hits	No PWM Hits
KH2012:KH.L96.11	Groucho2	-0.3220	-3.8274	-0.2602	-2.5437	Not Analyzed	No PWM match	No PWM match
KH2012:KH.C9.38	FUBP	-0.3542	-4.3098	-0.1160	-1.5991	Not Analyzed	No PWM match	No PWM match
KH2012:KH.C2.573	Smad1/5	-0.3728	-8.1941	-0.6434	-16.3524	Not Analyzed	No PWM Hits	No PWM Hits
KH2012:KH.L39.1	FoxH-b	-0.3728	-9.4982	0.4193	10.2540	Not Analyzed	-6.179	-1.6025
KH2012:KH.C12.116	Pax258-B	-0.3780	-6.4412	-0.1753	-1.8349	Not Analyzed	No PWM Hits	No PWM Hits
KH2012:KH.C4.84	Otx	-0.3833	-3.6292	-0.5285	-13.2944	Not Analyzed	-3.6335	-1.6025
KH2012:KH.L171.10	Hex	-0.4425	-13.9010	-0.0310	-1.9495	Not Analyzed	No PWM match	No PWM match
KH2012:KH.L108.4	CREB/ATF-a	-0.4650	-9.7664	0.0019	0.0314	Not Analyzed	-0.131	7.244
KH2012:KH.C11.657	Hlx	-0.5090	-11.5010	0.0000	0.0000	Not Analyzed	No PWM Hits	No PWM Hits
KH2012:KH.C1.959	Hox4	-0.5333	-16.4935	-0.0168	-0.5551	Not Analyzed	No PWM Hits	No PWM Hits
KH2012:KH.C7.157	Emc	-0.5383	-10.6758	0.8416	16.5105	Not Analyzed	No PWM match	No PWM match
KH2012:KH.C1.159	E(spl)/hairy-a	-0.5849	-12.9992	0.0718	0.2294	Not Analyzed	No PWM Hits	No PWM Hits
KH2012:KH.C4.182	Zinc Finger (C3H)	-0.6490	-13.5463	0.0789	1.1339	Not Analyzed	No PWM match	No PWM match
KH2012:KH.C3.312	E(spl)/hairy-b	-0.7259	-11.8914	0.2056	1.4126	Not Analyzed	No PWM Hits	No PWM Hits
KH2012:KH.C7.269	Orphan bHLH1	-1.1728	-7.9049	0.1181	2.3028	Not Analyzed	No PWM match	No PWM match
KH2012:KH.C11.314	Fos	-1.2680	-20.6461	0.0219	0.3596	Not Analyzed	No PWM Hits	No PWM Hits
KH2012:KH.C8.247	ELK	-1.3103	-35.3315	-0.4712	-8.9945	Not Analyzed	-12.0365	0.417
KH2012:KH.C4.539	Fli/ERG1	-1.3658	-29.2101	0.0283	0.6751	Not Analyzed	-18.0915	0.417
KH2012:KH.C11.313	FoxA-a	-1.7286	-46.5338	0.0201	1.1382	Not Analyzed	No PWM Hits	No PWM Hits
KH2012:KH.C5.202	TWIST-like-2	-1.9001	-30.4407	0.0142	0.6751	Not Analyzed	No PWM match	No PWM match
KH2012:KH.C5.416	TWIST-like-1a/b	-1.9623	-28.6308	0.0522	0.2435	Not Analyzed	No PWM match	No PWM match
KH2012:KH.S1404.1	Brachyury	-2.1781	-31.7672	-0.0310	-1.9495	Not Analyzed	13.761	13.761

JASPAR 2020 PWM cluster	Max Z-score in cluster	Max Z-score in cluster
BHLHA15_var_2	34.77	Ascl2
TBX21	20.92	MGA
MTF1	19.98	MTF1
NR3C1	19.27	Ar
DMRTC2	15.15	DMRTA2

B8.5

KH2012 gene model	TF common name	Log2FC Sibling Cluster	-Log(pvalue) Sibling Cluster	Log2FC Parent Cluster	-Log(pvalue) Parent Cluster	DE in Imai	Matched PWM z-score	PWM Cluster z-score
KH2012:KH.S423.6	SMYD1	0.7736	4.6337	0.7894	11.6176	No	No PWM match	No PWM match
KH2012:KH.C5.416	TWIST-like-1a/b	0.6881	6.9337	0.6714	15.6212	Yes	No PWM match	No PWM match
KH2012:KH.C7.111	Transcription initiation protein SPT4 homolog	0.4517	4.9659	-0.2510	-4.0975	No	No PWM match	No PWM match
KH2012:KH.L153.50	HsignallingT1; suppressor of S. cerevisiae gcr2	0.4102	6.1653	-0.2077	-3.7754	No	No PWM match	No PWM match
KH2012:KH.C11.351	NF45	0.3456	3.2830	-0.0403	-0.3118	No	No PWM match	No PWM match
KH2012:KH.C8.162	Zinc Finger (C2H2)-21	0.3390	4.2029	-0.0147	-0.3762	No	No PWM Hits	No PWM Hits
KH2012:KH.C5.362	CBF1 interacting corepressor-like	0.3123	4.1564	0.1467	2.5936	No	No PWM match	No PWM match
KH2012:KH.C14.178	HMG1/2	0.2743	4.1420	-0.1516	-4.8379	No	No PWM match	No PWM match
KH2012:KH.C14.307	MyoD	0.2563	3.1647	0.2232	4.9285	No	No PWM Hits	No PWM Hits
KH2012:KH.C3.103	RBP1-like	-0.2560	-3.5428	0.1560	2.4266	No	No PWM match	No PWM match
KH2012:KH.C4.509	Zinc Finger (absent, small, or homeotic discs 1)	-0.2758	-3.1184	0.5473	10.6109	No	No PWM match	No PWM match
KH2012:KH.L108.4	CREB/ATF-a	-0.2855	-5.0779	0.0209	0.2657	No	3.3415	9.524
KH2012:KH.C3.697	Zinc Finger (C2H2)-5	-0.2890	-3.5752	0.3056	4.1413	No	No PWM Hits	No PWM Hits
KH2012:KH.C1.661	MAD	-0.3015	-3.6533	-0.2650	-4.8235	No	No PWM match	No PWM match
KH2012:KH.C14.586	Zinc Finger (BTB/C2H2)	-0.3119	-3.5406	0.0882	0.8751	No	5.912	5.912
KH2012:KH.C4.739	capicua	-0.3119	-5.1514	-0.1276	-2.0118	No	No PWM match	No PWM match
KH2012:KH.C3.402	IRF-like7	-0.3119	-5.0629	0.0384	0.1610	No	No PWM match	No PWM match
KH2012:KH.C4.182	Zinc Finger (C3H)	-0.3585	-7.4955	0.0786	2.2023	No	No PWM match	No PWM match
KH2012:KH.C10.49	Bcl3	-0.3795	-3.7074	0.0410	0.1585	No	No PWM match	No PWM match
KH2012:KH.L24.23	Myc	-0.4248	-5.7668	0.1434	0.5622	No	No PWM Hits	No PWM Hits
KH2012:KH.C1.159	E(spl)/hairy-a	-0.4375	-3.9534	0.3235	6.2983	No	No PWM Hits	No PWM Hits
KH2012:KH.L13.24	FoxN2/3	-0.4452	-7.3928	0.2622	5.6417	Yes	-10.935	0.28
KH2012:KH.C14.449	CBP	-0.5237	-8.1955	0.3076	4.1813	No	No PWM match	No PWM match
KH2012:KH.L18.112	Zinc finger (PHD/NFX, DDT)	-0.5646	-5.6965	0.2189	2.4864	No	No PWM match	No PWM match
KH2012:KH.C3.230	Smad6/7	-0.6779	-4.7382	0.0009	0.4957	No	No PWM match	No PWM match
KH2012:KH.L4.17	Zinc Finger (C2H2)-18	-0.8917	-11.1157	0.5173	6.7879	No	-0.538	-0.538
KH2012:KH.C11.313	FoxA-a	-1.3394	-26.0203	0.0499	0.1828	No	No PWM Hits	No PWM Hits
KH2012:KH.C3.312	E(spl)/hairy-b	-1.3467	-24.8962	-0.1609	-2.4502	Yes	No PWM Hits	No PWM Hits
KH2012:KH.C8.247	ELK	-1.5315	-28.0617	0.7020	13.9342	Yes	-1.462	-0.1245
KH2012:KH.S1404.1	Brachyury	-2.1692	-40.6892	-0.2251	-3.3316	No	2.29	2.29

JASPAR 2020 PWM cluster	Max Z-score in cluster	Max Z-score in cluster
GLIS2	16.46	GLIS3
NFIL3	15.59	CEBPA

B8.5 Mesenchyme

KH2012 gene model	TF common name	Log2FC Sibling Cluster	-Log(pvalue) Sibling Cluster	Log2FC Parent Cluster	-Log(pvalue) Parent Cluster	DE in Imai	Matched PWM z-score	PWM Cluster z-score
KH2012:KH.C5.416	TWIST-like-1a/b	2.7537	29.0200	2.1041	34.5819	Not Analyzed	No PWM match	No PWM match
KH2012:KH.C5.202	TWIST-like-2	2.6481	29.7081	2.6216	62.4259	Not Analyzed	No PWM match	No PWM match
KH2012:KH.C4.539	Fli/ERG1	2.0348	28.6219	1.9442	54.7260	Not Analyzed	10.985	18.434
KH2012:KH.C11.314	Fos	1.9823	25.2886	1.9170	49.5380	Not Analyzed	No PWM Hits	No PWM Hits
KH2012:KH.C4.84	Otx	0.8992	12.7093	-0.2971	-1.8055	Not Analyzed	6.7145	12.32
KH2012:KH.C11.657	Hlx	0.8519	9.1164	0.8519	22.2239	Not Analyzed	No PWM Hits	No PWM Hits
KH2012:KH.L171.10	Hex	0.7510	11.2383	0.7510	27.1765	Not Analyzed	No PWM match	No PWM match
KH2012:KH.C1.959	Hox4	0.6842	8.6757	0.8190	28.5393	Not Analyzed	No PWM Hits	No PWM Hits
KH2012:KH.C14.278	Zinc Finger (C2H2)-34	0.5736	5.7100	-0.5872	-7.9953	Not Analyzed	No PWM Hits	No PWM Hits
KH2012:KH.C4.182	Zinc Finger (C3H)	0.5656	3.6475	0.8242	13.2576	Not Analyzed	No PWM match	No PWM match
KH2012:KH.C1.159	E(spl)/hairy-a	0.5502	6.3566	0.6637	12.7978	Not Analyzed	No PWM Hits	No PWM Hits
KH2012:KH.L24.23	Myc	0.5452	4.7756	0.2190	1.4797	Not Analyzed	No PWM Hits	No PWM Hits
KH2012:KH.L96.52	transcription factor4	0.5233	3.4445	-0.3160	-3.9459	Not Analyzed	5.608	9.5285
KH2012:KH.C7.702	Zinc Finger (BTB/POZ)	0.5159	3.9623	-0.2940	-3.6893	Not Analyzed	No PWM Hits	No PWM Hits
KH2012:KH.C11.485	Irx-B	0.4369	3.1790	-0.1715	-1.7897	Not Analyzed	No PWM Hits	No PWM Hits
KH2012:KH.S423.6	SMYD1	0.3973	5.2891	-0.4252	-1.0269	Not Analyzed	No PWM match	No PWM match
KH2012:KH.C7.294	Orphan bZIP-4	0.3149	4.6885	0.2884	7.6565	Not Analyzed	No PWM match	No PWM match
KH2012:KH.C7.782	POU-like	0.2833	3.8387	0.2856	4.5096	Not Analyzed	No PWM match	No PWM match
KH2012:KH.L108.4	CREB/ATF-a	-0.2892	-3.5843	0.2208	1.5692	Not Analyzed	2.472	9.5285
KH2012:KH.C1.1025	NF-X-like	-0.3705	-4.2455	-0.2740	-4.6751	Not Analyzed	No PWM match	No PWM match
KH2012:KH.C7.111	Transcription initiation protein SPT4 homolog	-0.3826	-4.0499	-0.7707	-12.4415	Not Analyzed	No PWM match	No PWM match
KH2012:KH.C11.706	Mlx	-0.4142	-3.2150	0.1389	0.9920	Not Analyzed	2.358	9.5285
KH2012:KH.L18.112	Zinc finger (PHD/NFX, DDT)	-0.4544	-5.9094	0.2291	1.5428	Not Analyzed	No PWM match	No PWM match
KH2012:KH.C11.313	FoxA-a	-0.4550	-3.1602	1.1887	19.4452	Not Analyzed	No PWM Hits	No PWM Hits
KH2012:KH.C7.157	Emc	-0.4764	-7.4842	0.2453	3.3743	Not Analyzed	No PWM match	No PWM match
KH2012:KH.C3.230	Smad6/7	-0.5477	-8.4726	-0.1978	-4.1853	Not Analyzed	No PWM match	No PWM match
KH2012:KH.L4.17	Zinc Finger (C2H2)-18	-1.1977	-19.7605	0.9379	20.9945	Not Analyzed	-2.805	-2.805
KH2012:KH.C7.269	Orphan bHLH1	-1.9271	-13.9170	0.0754	1.1859	Not Analyzed	No PWM match	No PWM match
KH2012:KH.S1404.1	Brachyury	-2.8158	-27.9015	-0.0401	-1.2629	Not Analyzed	-0.3545	15.92

JASPAR 2020 PWM cluster	Max Z-score in cluster	Max Z-score in cluster
ELK3	18.43	ETV5
ZNF282	17.09	ZNF282
PBX3	16.03	PKNOX1
TBX19	15.92	TBX19

B7.3

KH2012 gene model	TF common name	Log2FC Sibling Cluster	-Log(pvalue) Sibling Cluster	DE in Imai	Matched PWM z-score	PWM Cluster z-score
KH2012:KH.C11.485	Irx-B	0.7235	10.1545	No	No PWM Hits	No PWM Hits
KH2012:KH.L128.12	Mnx	0.5361	7.8130	Yes	-0.454	4.717
KH2012:KH.S215.4	Lhx3	0.5045	7.9421	Yes	1.531	4.717
KH2012:KH.S1404.1	Brachyury	0.3964	4.5174	No	5.021	5.021
KH2012:KH.C8.247	ELK	0.3911	3.4746	Yes	-2.522	13.202
KH2012:KH.C3.697	Zinc Finger (C2H2)-5	0.3881	4.2418	No	No PWM Hits	No PWM Hits
KH2012:KH.C1.27	LAG1-like1	0.3814	3.4259	No	No PWM match	No PWM match
KH2012:KH.C11.450	CBF	0.2816	3.1843	No	No PWM match	No PWM match
KH2012:KH.C11.481	IRF-like6	0.2775	3.1072	No	No PWM match	No PWM match
KH2012:KH.C3.17	ROR	-0.2579	-3.0918	No	0.6385	1.006
KH2012:KH.S816.1	ZicL	-0.2845	-3.1093	No	-5.087	9.132
KH2012:KH.C6.22	Smad2/3a	-0.2897	-3.8543	No	No PWM match	No PWM match
KH2012:KH.C1.264	Y-box protein1/2/3	-0.2929	-3.7661	No	No PWM match	No PWM match
KH2012:KH.C5.130	Zinc Finger (C2H2)-27	-0.3843	-4.3306	No	No PWM Hits	No PWM Hits
KH2012:KH.C14.520	Orphan Fox-1	-0.3943	-7.6506	No	No PWM Hits	No PWM Hits
KH2012:KH.S654.3	Tbx6b/c/d	-0.4281	-9.1086	No	No PWM match	No PWM match
KH2012:KH.C4.341	FoxB	-0.4473	-15.7021	No	3.74	6.895
KH2012:KH.C3.773	Ci-mT	-0.6972	-10.0735	No	No PWM match	No PWM match
KH2012:KH.S423.6	SMYD1	-0.8093	-23.1876	No	No PWM match	No PWM match
KH2012:KH.C14.307	MyoD	-1.0630	-35.5728	No	No PWM Hits	No PWM Hits

JASPAR 2020 PWM cluster	Max Z-score in cluster	Max Z-score in cluster

B8.6

KH2012 gene model	TF common name	Log2FC Sibling Cluster	-Log(pvalue) Sibling Cluster	Log2FC Parent Cluster	-Log(pvalue) Parent Cluster	DE in Imai	Matched PWM z-score	PWM Cluster z-score
KH2012:KH.S1404.1	Brachyury	2.1692	40.6892	1.9440	29.8462	Yes	-2.602	-0.108
KH2012:KH.C8.247	ELK	1.5315	28.0617	2.2334	35.4650	No	1.817	15.0955
KH2012:KH.C3.312	E(spl)/hairy-b	1.3467	24.8962	1.1858	19.3246	No	No PWM Hits	No PWM Hits
KH2012:KH.C11.313	FoxA-a	1.3394	26.0203	1.3893	25.2876	No	No PWM Hits	No PWM Hits
KH2012:KH.L4.17	Zinc Finger (C2H2)-18	0.8917	11.1157	1.4089	20.2211	No	-2.524	-2.524
KH2012:KH.C3.230	Smad6/7	0.6779	4.7382	0.6788	3.8868	No	No PWM match	No PWM match
KH2012:KH.L18.112	Zinc finger (PHD/NFX, DDT)	0.5646	5.6965	0.7835	9.0603	No	No PWM match	No PWM match
KH2012:KH.C14.449	CBP	0.5237	8.1955	0.8313	15.6228	No	No PWM match	No PWM match
KH2012:KH.L13.24	FoxN2/3	0.4452	7.3928	0.7074	13.7070	No	16.597	16.597
KH2012:KH.C1.159	E(spl)/hairy-a	0.4375	3.9534	0.7609	13.2706	No	No PWM Hits	No PWM Hits
KH2012:KH.L24.23	Myc	0.4248	5.7668	0.5683	7.6064	No	No PWM Hits	No PWM Hits
KH2012:KH.C10.49	Bcl3	0.3795	3.7074	0.4205	3.9449	No	No PWM match	No PWM match
KH2012:KH.C4.182	Zinc Finger (C3H)	0.3585	7.4955	0.4370	12.0953	No	No PWM match	No PWM match
KH2012:KH.C3.402	IRF-like7	0.3119	5.0629	0.3504	5.2097	No	No PWM match	No PWM match
KH2012:KH.C14.586	Zinc Finger (BTB/C2H2)	0.3119	3.5406	0.4002	4.8329	No	-7.65	-7.65
KH2012:KH.C4.739	capicua	0.3119	5.1514	0.1844	2.0384	No	No PWM match	No PWM match
KH2012:KH.C1.661	MAD	0.3015	3.6533	0.0366	0.2654	No	No PWM match	No PWM match
KH2012:KH.C3.697	Zinc Finger (C2H2)-5	0.2890	3.5752	0.5946	9.2122	No	No PWM Hits	No PWM Hits
KH2012:KH.L108.4	CREB/ATF-a	0.2855	5.0779	0.3064	4.6525	No	-2.5985	9.879
KH2012:KH.C4.509	Zinc Finger (absent, small, or homeotic discs 1)	0.2758	3.1184	0.8231	14.6335	No	No PWM match	No PWM match
KH2012:KH.C3.103	RBP1-like	0.2560	3.5428	0.4120	6.8699	No	No PWM match	No PWM match
KH2012:KH.C14.307	MyoD	-0.2563	-3.1647	-0.0332	-0.8733	No	No PWM Hits	No PWM Hits
KH2012:KH.C14.178	HMG1/2	-0.2743	-4.1420	-0.4259	-10.1179	No	No PWM match	No PWM match
KH2012:KH.C5.362	CBF1 interacting corepressor-like	-0.3123	-4.1564	-0.1656	-1.8011	No	No PWM match	No PWM match
KH2012:KH.C8.162	Zinc Finger (C2H2)-21	-0.3390	-4.2029	-0.3537	-4.9057	No	No PWM Hits	No PWM Hits
KH2012:KH.C11.351	NF45	-0.3456	-3.2830	-0.3859	-3.5162	No	No PWM match	No PWM match
KH2012:KH.L153.50	HsignallingT1; suppressor of S. cerevisiae gcr2	-0.4102	-6.1653	-0.6179	-10.6398	No	No PWM match	No PWM match
KH2012:KH.C7.111	Transcription initiation protein SPT4 homolog	-0.4517	-4.9659	-0.7027	-10.0289	No	No PWM match	No PWM match
KH2012:KH.C5.416	TWIST-like-1a/b	-0.6881	-6.9337	-0.0167	-0.5533	No	No PWM match	No PWM match
KH2012:KH.S423.6	SMYD1	-0.7736	-4.6337	0.0157	0.2839	No	No PWM match	No PWM match

JASPAR 2020 PWM cluster	Max Z-score in cluster	Max Z-score in cluster
RARA_var_2	20.12	RARA::RXRG
SOX8	17.72	Dmrt1
Foxf1	16.60	Foxj3
ELK3	15.10	ELK3

Secondary Notochord

KH2012 gene model	TF common name	Log2FC Sibling Cluster	-Log(pvalue) Sibling Cluster	Log2FC Parent Cluster	-Log(pvalue) Parent Cluster	DE in Imai	Matched PWM z-score	PWM Cluster z-score
KH2012:KH.S1404.1	Brachyury	2.8158	27.9015	0.6066	4.0874	Not Analyzed	0.217	0.217
KH2012:KH.C7.269	Orphan bHLH1	1.9271	13.9170	1.9800	11.3680	Not Analyzed	No PWM match	No PWM match
KH2012:KH.L4.17	Zinc Finger (C2H2)-18	1.1977	19.7605	1.2440	15.9932	Not Analyzed	0.322	0.322
KH2012:KH.C3.230	Smad6/7	0.5477	8.4726	-0.3280	-0.6065	Not Analyzed	No PWM match	No PWM match
KH2012:KH.C7.157	Emc	0.4764	7.4842	0.8249	9.5686	Not Analyzed	No PWM match	No PWM match
KH2012:KH.C11.313	FoxA-a	0.4550	3.1602	0.3043	1.5363	Not Analyzed	No PWM Hits	No PWM Hits
KH2012:KH.L18.112	Zinc finger (PHD/NFX, DDT)	0.4544	5.9094	0.1190	0.7009	Not Analyzed	No PWM match	No PWM match
KH2012:KH.C11.706	Mlx	0.4142	3.2150	0.4370	2.9680	Not Analyzed	-1.773	6.6685
KH2012:KH.C7.111	Transcription initiation protein SPT4 homolog	0.3826	4.0499	0.0636	0.2488	Not Analyzed	No PWM match	No PWM match
KH2012:KH.C1.1025	NF-X-like	0.3705	4.2455	0.0111	0.0158	Not Analyzed	No PWM match	No PWM match
KH2012:KH.L108.4	CREB/ATF-a	0.2892	3.5843	0.2246	1.5628	Not Analyzed	-1.5695	6.6685
KH2012:KH.C7.782	POU-like	-0.2833	-3.8387	0.0663	0.0703	Not Analyzed	No PWM match	No PWM match
KH2012:KH.C7.294	Orphan bZIP-4	-0.3149	-4.6885	0.0000	0.0000	Not Analyzed	No PWM match	No PWM match
KH2012:KH.S423.6	SMYD1	-0.3973	-5.2891	-0.0489	-1.3028	Not Analyzed	No PWM match	No PWM match
KH2012:KH.C11.485	Irx-B	-0.4369	-3.1790	-0.8109	-9.7247	Not Analyzed	No PWM Hits	No PWM Hits
KH2012:KH.C7.702	Zinc Finger (BTB/POZ)	-0.5159	-3.9623	-0.9302	-16.2795	Not Analyzed	No PWM Hits	No PWM Hits
KH2012:KH.L96.52	transcription factor4	-0.5233	-3.4445	-0.7463	-8.4283	Not Analyzed	-4.3055	6.6685
KH2012:KH.L24.23	Myc	-0.5452	-4.7756	-0.7511	-9.3857	Not Analyzed	No PWM Hits	No PWM Hits
KH2012:KH.C1.159	E(spl)/hairy-a	-0.5502	-6.3566	-0.3240	-2.2442	Not Analyzed	No PWM Hits	No PWM Hits
KH2012:KH.C4.182	Zinc Finger (C3H)	-0.5656	-3.6475	-0.0999	-1.4206	Not Analyzed	No PWM match	No PWM match
KH2012:KH.C14.278	Zinc Finger (C2H2)-34	-0.5736	-5.7100	-1.3889	-18.2598	Not Analyzed	No PWM Hits	No PWM Hits
KH2012:KH.C1.959	Hox4	-0.6842	-8.6757	0.1481	2.0896	Not Analyzed	No PWM Hits	No PWM Hits
KH2012:KH.L171.10	Hex	-0.7510	-11.2383	0.0000	0.0000	Not Analyzed	No PWM match	No PWM match
KH2012:KH.C11.657	Hlx	-0.8519	-9.1164	0.0000	0.0000	Not Analyzed	No PWM Hits	No PWM Hits
KH2012:KH.C4.84	Otx	-0.8992	-12.7093	-0.8820	-15.7758	Not Analyzed	-5.4135	0.924
KH2012:KH.C11.314	Fos	-1.9823	-25.2886	0.0000	0.0000	Not Analyzed	No PWM Hits	No PWM Hits
KH2012:KH.C4.539	Fli/ERG1	-2.0348	-28.6219	0.0000	0.0000	Not Analyzed	-8.07	2.382
KH2012:KH.C5.202	TWIST-like-2	-2.6481	-29.7081	-0.0489	-1.3028	Not Analyzed	No PWM match	No PWM match
KH2012:KH.C5.416	TWIST-like-1a/b	-2.7537	-29.0200	0.0385	0.9348	Not Analyzed	No PWM match	No PWM match

JASPAR 2020 PWM cluster	Max Z-score in cluster	Max Z-score in cluster
ZNF135	17.20	ZNF135
POU3F1	15.42	POU2F3
OVOL2	15.12	MYBL2

B7.6

KH2012 gene model	TF common name	Log2FC Sibling Cluster	-Log(pvalue) Sibling Cluster	DE in Imai	Matched PWM z-score	PWM Cluster z-score
KH2012:KH.C1.727	macho-1	1.9316	40.9756	No	No PWM Hits	No PWM Hits
KH2012:KH.C1.1016	GCNF	0.7263	9.3521	No	No PWM match	No PWM match
KH2012:KH.C8.450	Nkx-A	0.5219	6.4111	No	No PWM match	No PWM match
KH2012:KH.C1.777	Zinc Finger (C2H2)-33	0.3949	6.5328	No	No PWM Hits	No PWM Hits
KH2012:KH.C8.419	HMG2-like	0.3128	4.4722	No	No PWM match	No PWM match
KH2012:KH.C14.105	CNOT7/8	-0.2572	-3.4274	No	No PWM match	No PWM match
KH2012:KH.C14.586	Zinc Finger (BTB/C2H2)	-0.2622	-3.8794	No	7.6095	7.6095
KH2012:KH.C7.269	Orphan bHLH1	-0.2667	-4.0886	No	No PWM match	No PWM match
KH2012:KH.C10.245	AF10like	-0.2733	-3.6483	No	No PWM match	No PWM match
KH2012:KH.S899.4	BTF3	-0.2784	-5.9441	No	No PWM match	No PWM match
KH2012:KH.C14.306	IRF-like 2	-0.3061	-3.2404	No	No PWM match	No PWM match
KH2012:KH.C3.751	Snail	-0.3258	-7.2275	No	No PWM Hits	No PWM Hits
KH2012:KH.C1.677	Smcx/SMCY/PLU-1/RBP-2 like 1	-0.3471	-4.0524	No	No PWM match	No PWM match
KH2012:KH.C3.773	Ci-mT	-0.3665	-4.7200	No	No PWM match	No PWM match
KH2012:KH.C3.312	E(spl)/hairy-b	-0.4030	-5.8955	No	No PWM Hits	No PWM Hits
KH2012:KH.S816.1	ZicL	-0.4436	-9.1350	No	-8.9325	7.8685
KH2012:KH.S654.3	Tbx6b/c/d	-0.5126	-11.1306	No	No PWM match	No PWM match
KH2012:KH.C4.84	Otx	-0.6887	-10.9042	No	5.5315	9.407

JASPAR 2020 PWM cluster	Max Z-score in cluster	Max Z-score in cluster
E2F7	16.59	E2F6

B7.6

KH2012 gene model	TF common name	Log2FC Sibling Cluster	-Log(pvalue) Sibling Cluster	Log2FC Parent Cluster	-Log(pvalue) Parent Cluster	DE in Imai	Matched PWM z-score	PWM Cluster z-score
KH2012:KH.C1.727	macho-1	3.1599	12.3008	0.9740	1.2354	No	No PWM Hits	No PWM Hits
KH2012:KH.C1.777	Zinc Finger (C2H2)-33	1.2621	5.1755	1.2870	6.4260	No	No PWM Hits	No PWM Hits
KH2012:KH.C10.92	Baf57	0.7475	5.2319	0.7349	5.6116	No	No PWM match	No PWM match
KH2012:KH.L150.2	Orphan Fox-2	-0.3898	-3.1241	0.1061	0.1502	No	No PWM Hits	No PWM Hits
KH2012:KH.C8.419	HMG2-like	-0.4214	-3.0126	-0.5607	-3.5579	No	No PWM match	No PWM match
KH2012:KH.L96.52	transcription factor4	-0.4901	-3.1648	-0.6367	-4.3996	No	-1.301	5.986
KH2012:KH.C5.90	core-binding factor, β subunit	-0.5496	-2.9995	-0.4309	-1.7030	No	No PWM match	No PWM match
KH2012:KH.L18.112	Zinc finger (PHD/NFX, DDT)	-0.5641	-3.2458	-0.2895	-1.0135	No	No PWM match	No PWM match
KH2012:KH.C1.661	MAD	-0.6122	-4.2908	-0.2565	-0.6494	No	No PWM match	No PWM match
KH2012:KH.L122.11	metastasis associated protein	-0.6122	-5.3100	-0.0252	-0.0076	No	No PWM match	No PWM match
KH2012:KH.C1.159	E(spl)/hairy-a	-0.6308	-4.4020	0.0000	0.0000	No	No PWM Hits	No PWM Hits
KH2012:KH.C14.377	Otp	-0.6897	-4.7623	0.0000	0.0000	No	No PWM Hits	No PWM Hits
KH2012:KH.C1.397	NGFI-A binding protein 43832	-0.7276	-4.2267	-0.3036	-1.3739	No	No PWM match	No PWM match
KH2012:KH.C11.485	Irx-B	-0.7853	-4.4126	0.3479	8.7098	No	No PWM Hits	No PWM Hits
KH2012:KH.L8.11	Tbx6a	-0.8048	-4.3940	0.2410	6.0476	No	No PWM match	No PWM match
KH2012:KH.C8.143	PPAR	-0.8463	-5.1271	-0.4800	-1.1509	No	-2.362	9.636
KH2012:KH.C3.348	E12/E47	-0.9992	-6.4359	-0.1655	-0.7367	No	No PWM Hits	No PWM Hits
KH2012:KH.C4.84	Otx	-1.2293	-6.3394	2.2655	12.2383	No	2.2635	3.1405
KH2012:KH.S215.4	Lhx3	-1.2918	-6.5507	0.7800	6.8327	No	-2.403	9.241
KH2012:KH.S816.1	ZicL	-1.4057	-7.5892	0.4475	11.4095	No	1.4765	3.5765
KH2012:KH.C14.520	Orphan Fox-1	-1.4213	-8.0450	-0.3777	-1.8407	No	No PWM Hits	No PWM Hits
KH2012:KH.C8.247	ELK	-1.5927	-11.2808	0.3801	4.2585	No	-0.7865	4.147
KH2012:KH.C3.100	Mesp	-1.9021	-10.5195	0.4475	6.0462	No	No PWM match	No PWM match
KH2012:KH.C3.751	Snail	-2.2131	-12.7136	0.4475	6.0462	No	No PWM Hits	No PWM Hits
KH2012:KH.S654.3	Tbx6b/c/d	-2.2668	-12.9287	0.5406	8.7057	No	No PWM match	No PWM match

JASPAR 2020 PWM cluster	Max Z-score in cluster	Max Z-score in cluster
Nr2f6	15.13	NR1H2::RXRA

B7.6

KH2012 gene model	TF common name	Log2FC Sibling Cluster	-Log(pvalue) Sibling Cluster	Log2FC Parent Cluster	-Log(pvalue) Parent Cluster	DE in Imai	Matched PWM z-score	PWM Cluster z-score
KH2012:KH.C1.727	macho-1	2.8759	18.5754	-0.3964	-0.3640	Not Analyzed	No PWM Hits	No PWM Hits
KH2012:KH.C1.1016	GCNF	1.8294	9.9823	1.0644	2.5213	Not Analyzed	No PWM match	No PWM match
KH2012:KH.C8.450	Nkx-A	0.7875	4.5865	0.0380	0.0434	Not Analyzed	No PWM match	No PWM match
KH2012:KH.C8.419	HMG2-like	0.4630	3.5262	0.2708	0.9921	Not Analyzed	No PWM match	No PWM match
KH2012:KH.C12.116	Pax258-B	0.4566	3.4911	0.1208	0.4735	Not Analyzed	No PWM Hits	No PWM Hits
KH2012:KH.C1.99	SoxB1	0.4420	6.2662	0.5525	4.5427	Not Analyzed	No PWM match	No PWM match
KH2012:KH.C2.573	Smad1/5	0.3833	3.8821	0.3720	2.8406	Not Analyzed	No PWM Hits	No PWM Hits
KH2012:KH.L39.1	FoxH-b	0.3421	3.0246	0.8745	7.3861	Not Analyzed	-0.8375	0.6645
KH2012:KH.C9.580	RAR	0.2932	3.4451	0.4105	2.9090	Not Analyzed	0.7575	4.6885
KH2012:KH.C11.450	CBF	-0.3895	-3.2553	-0.0069	-0.1353	Not Analyzed	No PWM match	No PWM match
KH2012:KH.L53.12	Zinc Finger (C2H2)-15	-0.3952	-3.5033	-0.7105	-4.6785	Not Analyzed	No PWM Hits	No PWM Hits
KH2012:KH.C5.130	Zinc Finger (C2H2)-27	-0.4222	-3.0732	-0.3886	-1.4268	Not Analyzed	No PWM Hits	No PWM Hits
KH2012:KH.C1.275	STAT-b	-0.4890	-3.4014	-0.2870	-2.5493	Not Analyzed	No PWM match	No PWM match
KH2012:KH.C14.520	Orphan Fox-1	-0.5074	-6.0512	0.6032	3.1335	Not Analyzed	No PWM Hits	No PWM Hits
KH2012:KH.C5.362	CBF1 interacting corepressor-like	-0.5108	-5.1956	-0.7058	-3.1547	Not Analyzed	No PWM match	No PWM match
KH2012:KH.L165.2	HIV TAT specific factor 1 (HTATSF1)	-0.5481	-6.2538	0.0578	0.1631	Not Analyzed	No PWM match	No PWM match
KH2012:KH.S406.15	Serum response factor	-0.5742	-3.5557	-0.5406	-4.2256	Not Analyzed	-2.304	-2.304
KH2012:KH.S215.4	Lhx3	-0.5850	-3.0967	-0.5995	-1.7089	Not Analyzed	-2.864	4.729
KH2012:KH.C1.661	MAD	-0.5850	-5.1366	-0.1479	-0.9189	Not Analyzed	No PWM match	No PWM match
KH2012:KH.L128.12	Mnx	-0.6202	-4.0467	0.1375	0.2966	Not Analyzed	-0.487	4.729
KH2012:KH.C3.312	E(spl)/hairy-b	-0.6265	-4.6016	-0.3695	-2.3375	Not Analyzed	No PWM Hits	No PWM Hits
KH2012:KH.L96.87	Tbx2/3	-0.6374	-5.4490	0.0000	0.0000	Not Analyzed	-11.637	5.2865
KH2012:KH.L153.85	Zinc Finger (C2H2)-8	-0.6630	-3.5719	-0.6491	-1.7306	Not Analyzed	0.1165	0.1575
KH2012:KH.C3.348	E12/E47	-0.6794	-7.7291	0.1692	0.3710	Not Analyzed	No PWM Hits	No PWM Hits
KH2012:KH.C8.247	ELK	-0.6881	-5.6484	0.1375	0.0521	Not Analyzed	3.1135	5.745
KH2012:KH.S899.4	BTF3	-0.7251	-7.3973	-0.2349	-0.5517	Not Analyzed	No PWM match	No PWM match
KH2012:KH.C3.230	Smad6/7	-0.7370	-5.0586	-0.1674	-1.5399	Not Analyzed	No PWM match	No PWM match
KH2012:KH.L4.17	Zinc Finger (C2H2)-18	-0.7834	-8.5161	0.3797	1.8484	Not Analyzed	-4.044	-4.044
KH2012:KH.L9.33	E(Y)2	-1.0120	-4.6818	-0.5850	-3.3360	Not Analyzed	No PWM match	No PWM match
KH2012:KH.C3.17	ROR	-1.0745	-7.5994	-0.0755	-0.0493	Not Analyzed	2.408	11.115
KH2012:KH.C11.485	Irx-B	-1.0825	-7.9398	-0.1674	-1.5399	Not Analyzed	No PWM Hits	No PWM Hits
KH2012:KH.C14.307	MyoD	-1.4150	-8.6228	0.0931	0.8295	Not Analyzed	No PWM Hits	No PWM Hits
KH2012:KH.C14.377	Otp	-1.6428	-12.3136	0.3410	1.4352	Not Analyzed	No PWM Hits	No PWM Hits
KH2012:KH.S654.3	Tbx6b/c/d	-1.8329	-13.7569	-0.4475	-1.8364	Not Analyzed	No PWM match	No PWM match
KH2012:KH.C3.751	Snail	-2.0797	-14.1779	0.1051	0.0945	Not Analyzed	No PWM Hits	No PWM Hits
KH2012:KH.C4.84	Otx	-2.4188	-15.4085	-1.6699	-3.5631	Not Analyzed	-5.384	0.6645
KH2012:KH.C3.100	Mesp	-2.7496	-16.2290	-0.2669	-0.9163	Not Analyzed	No PWM match	No PWM match
KH2012:KH.S423.6	SMYD1	-3.1278	-14.5430	0.2630	1.4337	Not Analyzed	No PWM match	No PWM match

JASPAR 2020 PWM cluster	Max Z-score in cluster	Max Z-score in cluster
BHLHA15_var_2	15.19	SNAI1

B7.5

KH2012 gene model	TF common name	Log2FC Sibling Cluster	-Log(pvalue) Sibling Cluster	DE in Imai	Matched PWM z-score	PWM Cluster z-score
KH2012:KH.C4.84	Otx	0.6887	10.9042	Yes	-5.829	2.144
KH2012:KH.S654.3	Tbx6b/c/d	0.5126	11.1306	Yes	No PWM match	No PWM match
KH2012:KH.S816.1	ZicL	0.4436	9.1350	Yes	16.556	16.556
KH2012:KH.C3.312	E(spl)/hairy-b	0.4030	5.8955	No	No PWM Hits	No PWM Hits
KH2012:KH.C3.773	Ci-mT	0.3665	4.7200	No	No PWM match	No PWM match
KH2012:KH.C1.677	Smcx/SMCY/PLU-1/RBP-2 like 1	0.3471	4.0524	No	No PWM match	No PWM match
KH2012:KH.C3.751	Snail	0.3258	7.2275	No	No PWM Hits	No PWM Hits
KH2012:KH.C14.306	IRF-like 2	0.3061	3.2404	No	No PWM match	No PWM match
KH2012:KH.S899.4	BTF3	0.2784	5.9441	No	No PWM match	No PWM match
KH2012:KH.C10.245	AF10like	0.2733	3.6483	No	No PWM match	No PWM match
KH2012:KH.C7.269	Orphan bHLH1	0.2667	4.0886	No	No PWM match	No PWM match
KH2012:KH.C14.586	Zinc Finger (BTB/C2H2)	0.2622	3.8794	No	-6.514	-6.514
KH2012:KH.C14.105	CNOT7/8	0.2572	3.4274	No	No PWM match	No PWM match
KH2012:KH.C8.419	HMG2-like	-0.3128	-4.4722	No	No PWM match	No PWM match
KH2012:KH.C1.777	Zinc Finger (C2H2)-33	-0.3949	-6.5328	No	No PWM Hits	No PWM Hits
KH2012:KH.C8.450	Nkx-A	-0.5219	-6.4111	No	No PWM match	No PWM match
KH2012:KH.C1.1016	GCNF	-0.7263	-9.3521	No	No PWM match	No PWM match
KH2012:KH.C1.727	macho-1	-1.9316	-40.9756	No	No PWM Hits	No PWM Hits

JASPAR 2020 PWM cluster	Max Z-score in cluster	Max Z-score in cluster
IRF8	28.65	IRF5
FOSL2::JUNB_var_2	21.48	ATF7
GLIS2	16.56	ZIC4

B7.5

KH2012 gene model	TF common name	Log2FC Sibling Cluster	-Log(pvalue) Sibling Cluster	Log2FC Parent Cluster	-Log(pvalue) Parent Cluster	DE in Imai	Matched PWM z-score	PWM Cluster z-score
KH2012:KH.S654.3	Tbx6b/c/d	2.2668	12.9287	2.2947	37.1606	Yes	No PWM match	No PWM match
KH2012:KH.C3.751	Snail	2.2131	12.7136	2.3347	40.8608	Yes	No PWM Hits	No PWM Hits
KH2012:KH.C3.100	Mesp	1.9021	10.5195	2.3496	48.7534	Yes	No PWM match	No PWM match
KH2012:KH.C8.247	ELK	1.5927	11.2808	1.7922	36.7230	No	1.89	10.5045
KH2012:KH.C14.520	Orphan Fox-1	1.4213	8.0450	0.9905	13.3935	Yes	No PWM Hits	No PWM Hits
KH2012:KH.S816.1	ZicL	1.4057	7.5892	1.4096	22.1421	Yes	-3.0815	9.86
KH2012:KH.S215.4	Lhx3	1.2918	6.5507	1.8913	30.7571	Yes	5.2705	13.003
KH2012:KH.C4.84	Otx	1.2293	6.3394	2.8060	37.4612	Yes	-4.755	5.9955
KH2012:KH.C3.348	E12/E47	0.9992	6.4359	0.8113	13.2929	Yes	No PWM Hits	No PWM Hits
KH2012:KH.C8.143	PPAR	0.8463	5.1271	0.5684	7.1654	No	No PWM Hits	-8.729
KH2012:KH.L8.11	Tbx6a	0.8048	4.3940	1.0267	27.6770	Yes	No PWM match	No PWM match
KH2012:KH.C11.485	Irx-B	0.7853	4.4126	1.0953	30.6687	No	No PWM Hits	No PWM Hits
KH2012:KH.C1.397	NGFI-A binding protein 43832	0.7276	4.2267	0.2323	1.8092	No	No PWM match	No PWM match
KH2012:KH.C14.377	Otp	0.6897	4.7623	0.6897	21.2776	No	No PWM Hits	No PWM Hits
KH2012:KH.C1.159	E(spl)/hairy-a	0.6308	4.4020	0.5742	14.2368	No	No PWM Hits	No PWM Hits
KH2012:KH.L122.11	metastasis associated protein	0.6122	5.3100	0.5532	11.4841	No	No PWM match	No PWM match
KH2012:KH.C1.661	MAD	0.6122	4.2908	0.3271	5.4833	No	No PWM match	No PWM match
KH2012:KH.L18.112	Zinc finger (PHD/NFX, DDT)	0.5641	3.2458	0.1459	1.3842	No	No PWM match	No PWM match
KH2012:KH.C5.90	core-binding factor, β subunit	0.5496	2.9995	0.1292	1.0957	No	No PWM match	No PWM match
KH2012:KH.L96.52	transcription factor4	0.4901	3.1648	-0.0624	-0.3724	No	2.7755	11.168
KH2012:KH.C8.419	HMG2-like	0.4214	3.0126	0.1734	3.0404	No	No PWM match	No PWM match
KH2012:KH.L150.2	Orphan Fox-2	0.3898	3.1241	0.3360	5.7807	No	No PWM Hits	No PWM Hits
KH2012:KH.C10.92	Baf57	-0.7475	-5.2319	0.1150	1.4655	No	No PWM match	No PWM match
KH2012:KH.C1.777	Zinc Finger (C2H2)-33	-1.2621	-5.1755	0.4198	8.0445	No	No PWM Hits	No PWM Hits
KH2012:KH.C1.727	macho-1	-3.1599	-12.3008	-0.2543	-2.9187	No	No PWM Hits	No PWM Hits

JASPAR 2020 PWM cluster	Max Z-score in cluster	Max Z-score in cluster
YY1	29.18	YY1
Hmx1	25.60	Alx1
SOX8	22.30	Sox11
SRF	21.69	SRF
ZBTB7B	18.75	ZBTB7C
ZNF274	17.54	ZNF274
TBX21	17.38	TBX20
ZSCAN4	17.18	ZSCAN4
ZNF282	16.64	ZNF282
PAX1	16.18	PAX9
GATA2	15.81	Mecom
TFAP2B	15.56	EBF3

Trunk Ventral Cells

KH2012 gene model	TF common name	Log2FC Sibling Cluster	-Log(pvalue) Sibling Cluster	Log2FC Parent Cluster	-Log(pvalue) Parent Cluster	DE in Imai	Matched PWM z-score	PWM Cluster z-score
KH2012:KH.S423.6	SMYD1	3.1278	14.5430	3.3006	25.2753	Not Analyzed	No PWM match	No PWM match
KH2012:KH.C3.100	Mesp	2.7496	16.2290	0.5806	4.1242	Not Analyzed	No PWM match	No PWM match
KH2012:KH.C4.84	Otx	2.4188	15.4085	-0.4803	-6.2437	Not Analyzed	8.7705	14.516
KH2012:KH.C3.751	Snail	2.0797	14.1779	-0.0282	-0.1119	Not Analyzed	No PWM Hits	No PWM Hits
KH2012:KH.S654.3	Tbx6b/c/d	1.8329	13.7569	-0.8814	-12.7978	Not Analyzed	No PWM match	No PWM match
KH2012:KH.C14.377	Otp	1.6428	12.3136	1.2942	14.7231	Not Analyzed	No PWM Hits	No PWM Hits
KH2012:KH.C14.307	MyoD	1.4150	8.6228	1.2529	11.9404	Not Analyzed	No PWM Hits	No PWM Hits
KH2012:KH.C11.485	Irx-B	1.0825	7.9398	0.1298	0.5131	Not Analyzed	No PWM Hits	No PWM Hits
KH2012:KH.C3.17	ROR	1.0745	7.5994	1.1890	14.1725	Not Analyzed	-3.449	3.244
KH2012:KH.L9.33	E(Y)2	1.0120	4.6818	-0.0945	-1.0150	Not Analyzed	No PWM match	No PWM match
KH2012:KH.L4.17	Zinc Finger (C2H2)-18	0.7834	8.5161	1.4026	20.8782	Not Analyzed	0.815	0.815
KH2012:KH.C3.230	Smad6/7	0.7370	5.0586	-0.1944	-0.4587	Not Analyzed	No PWM match	No PWM match
KH2012:KH.S899.4	BTF3	0.7251	7.3973	0.3475	3.4678	Not Analyzed	No PWM match	No PWM match
KH2012:KH.C8.247	ELK	0.6881	5.6484	-0.7671	-10.0977	Not Analyzed	-4.3535	12.645
KH2012:KH.C3.348	E12/E47	0.6794	7.7291	-0.1506	-0.7377	Not Analyzed	No PWM Hits	No PWM Hits
KH2012:KH.L153.85	Zinc Finger (C2H2)-8	0.6630	3.5719	-0.1175	-1.6325	Not Analyzed	-0.113	14.9985
KH2012:KH.L96.87	Tbx2/3	0.6374	5.4490	0.3437	2.5801	Not Analyzed	19.8955	19.8955
KH2012:KH.C3.312	E(spl)/hairy-b	0.6265	4.6016	-0.1657	-1.1585	Not Analyzed	No PWM Hits	No PWM Hits
KH2012:KH.L128.12	Mnx	0.6202	4.0467	0.7930	9.4872	Not Analyzed	0.721	16.257
KH2012:KH.C1.661	MAD	0.5850	5.1366	-0.1751	-1.3956	Not Analyzed	No PWM match	No PWM match
KH2012:KH.S215.4	Lhx3	0.5850	3.0967	-1.3063	-14.7164	Not Analyzed	5.2075	16.257
KH2012:KH.S406.15	Serum response factor	0.5742	3.5557	-0.0565	-1.4114	Not Analyzed	4.188	4.188
KH2012:KH.L165.2	HIV TAT specific factor 1 (HTATSF1)	0.5481	6.2538	0.6798	10.7529	Not Analyzed	No PWM match	No PWM match
KH2012:KH.C5.362	CBF1 interacting corepressor-like	0.5108	5.1956	-0.0456	-1.3083	Not Analyzed	No PWM match	No PWM match
KH2012:KH.C14.520	Orphan Fox-1	0.5074	6.0512	-0.3107	-2.0070	Not Analyzed	No PWM Hits	No PWM Hits
KH2012:KH.C1.275	STAT-b	0.4890	3.4014	-0.0486	-0.2054	Not Analyzed	No PWM match	No PWM match
KH2012:KH.C5.130	Zinc Finger (C2H2)-27	0.4222	3.0732	-0.0961	-0.8466	Not Analyzed	No PWM Hits	No PWM Hits
KH2012:KH.L53.12	Zinc Finger (C2H2)-15	0.3952	3.5033	-0.0145	-0.0752	Not Analyzed	No PWM Hits	No PWM Hits
KH2012:KH.C11.450	CBF	0.3895	3.2553	0.2810	2.6519	Not Analyzed	No PWM match	No PWM match
KH2012:KH.C9.580	RAR	-0.2932	-3.4451	0.3642	2.9809	Not Analyzed	-1.2245	-1.2245
KH2012:KH.L39.1	FoxH-b	-0.3421	-3.0246	0.3642	4.9923	Not Analyzed	2.484	14.516
KH2012:KH.C2.573	Smad1/5	-0.3833	-3.8821	0.0163	0.8948	Not Analyzed	No PWM Hits	No PWM Hits
KH2012:KH.C1.99	SoxB1	-0.4420	-6.2662	0.1903	2.8740	Not Analyzed	No PWM match	No PWM match
KH2012:KH.C12.116	Pax258-B	-0.4566	-3.4911	-0.4093	-4.3151	Not Analyzed	No PWM Hits	No PWM Hits
KH2012:KH.C8.419	HMG2-like	-0.4630	-3.5262	-0.6136	-9.5364	Not Analyzed	No PWM match	No PWM match
KH2012:KH.C8.450	Nkx-A	-0.7875	-4.5865	-0.2058	-1.4051	Not Analyzed	No PWM match	No PWM match
KH2012:KH.C1.1016	GCNF	-1.8294	-9.9823	-0.0071	-0.1529	Not Analyzed	No PWM match	No PWM match
KH2012:KH.C1.727	macho-1	-2.8759	-18.5754	-0.1124	-1.6842	Not Analyzed	No PWM Hits	No PWM Hits

JASPAR 2020 PWM cluster	Max Z-score in cluster	Max Z-score in cluster
IRF8	28.81	IRF3
MTF1	27.02	MTF1
TBX21	19.90	TBX2
Rhox11	19.17	Rhox11
SP3	19.15	SP2
ARNT2	19.02	CREB3
PBX3	18.68	PBX3
Hmx1	17.41	Alx1
PDX1	16.26	HESX1
TFAP2B	15.62	Ebf2

B7.8

KH2012 gene model	TF common name	Log2FC Sibling Cluster	-Log(pvalue) Sibling Cluster	DE in Imai	Matched PWM z-score	PWM Cluster z-score
KH2012:KH.C14.307	MyoD	0.6898	27.3493	Yes	No PWM Hits	No PWM Hits
KH2012:KH.C3.773	Ci-mT	0.6625	16.9725	No	No PWM match	No PWM match
KH2012:KH.C3.751	Snail	0.5435	10.6236	No	No PWM Hits	No PWM Hits
KH2012:KH.S654.3	Tbx6b/c/d	0.4464	11.7331	No	No PWM match	No PWM match
KH2012:KH.S423.6	SMYD1	0.3004	10.8801	No	No PWM match	No PWM match
KH2012:KH.C14.377	Otp	0.2863	12.6843	Yes	No PWM Hits	No PWM Hits
KH2012:KH.C14.520	Orphan Fox-1	0.2845	8.6015	No	No PWM Hits	No PWM Hits
KH2012:KH.C5.130	Zinc Finger (C2H2)-27	-0.2562	-5.2742	No	No PWM Hits	No PWM Hits
KH2012:KH.L18.112	Zinc finger (PHD/NFX, DDT)	-0.2934	-3.4130	No	No PWM match	No PWM match
KH2012:KH.C8.247	ELK	-0.3124	-5.2856	No	5.4945	6.822
KH2012:KH.C5.416	TWIST-like-1a/b	-0.4371	-12.8343	No	No PWM match	No PWM match
KH2012:KH.C11.485	Irx-B	-0.5107	-8.7520	No	No PWM Hits	No PWM Hits

JASPAR 2020 PWM cluster	Max Z-score in cluster	Max Z-score in cluster
PROX1	34.57	PROX1
GLIS2	29.95	ZIC1
TBX21	26.12	TBR1
PHOX2A	24.65	DUXA
HSF2	23.46	HSF2
IRF8	23.10	STAT1::STAT2
BHLHA15_var_2	23.05	TCF3
Prdm15	21.15	Prdm15
TFAP2B	21.15	Znf423
Plagl1	19.63	Plagl1
Nr2f6	18.53	TCF7L2
ZFP57	17.76	ZFP57
FOS::JUNB	16.15	MAFK
INSM1	15.76	CTCFL
PBX3	15.38	PKNOX1

B7.8 Muscle

KH2012 gene model	TF common name	Log2FC Sibling Cluster	-Log(pvalue) Sibling Cluster	Log2FC Parent Cluster	-Log(pvalue) Parent Cluster	DE in Imai	Matched PWM z-score	PWM Cluster z-score
KH2012:KH.S423.6	SMYD1	3.4785	31.1062	3.3190	63.0053	Yes	No PWM match	No PWM match
KH2012:KH.C14.377	Otp	2.0204	30.8285	1.8750	58.9773	Yes	No PWM Hits	No PWM Hits
KH2012:KH.C14.307	MyoD	1.9778	27.1326	1.3474	36.0579	Yes	No PWM Hits	No PWM Hits
KH2012:KH.S654.3	Tbx6b/c/d	1.4002	22.0302	1.1624	33.8293	Yes	No PWM match	No PWM match
KH2012:KH.C3.773	Ci-mT	0.8429	8.0740	-0.6587	-12.1297	No	No PWM match	No PWM match
KH2012:KH.L4.17	Zinc Finger (C2H2)-18	0.8329	7.7609	0.8043	12.2034	No	24.52	24.52
KH2012:KH.C1.661	MAD	0.5399	6.1518	0.2531	3.0959	No	No PWM match	No PWM match
KH2012:KH.C14.520	Orphan Fox-1	0.4920	6.4783	-0.1061	-1.4708	No	No PWM Hits	No PWM Hits
KH2012:KH.C3.17	ROR	0.4458	4.9430	0.5035	10.0407	No	3.7165	6.278
KH2012:KH.L128.12	Mnx	0.4371	3.4179	0.4371	8.3670	No	5.427	12.021
KH2012:KH.L41.39	Orphan bHLH-4	0.3981	3.7886	0.4754	8.7301	No	No PWM match	No PWM match
KH2012:KH.C11.450	CBF	0.3853	4.3658	0.0837	1.3707	No	No PWM match	No PWM match
KH2012:KH.L39.1	FoxH-b	0.2996	3.7250	-0.0669	-0.9924	No	-0.65	-0.65
KH2012:KH.C3.697	Zinc Finger (C2H2)-5	0.2944	3.0427	0.4080	7.4580	No	No PWM Hits	No PWM Hits
KH2012:KH.C9.580	RAR	0.2717	3.1355	0.1236	1.7651	No	-7.038	-4.261
KH2012:KH.C14.306	IRF-like 2	0.2609	3.0892	0.0816	1.4404	No	No PWM match	No PWM match
KH2012:KH.C2.161	FXR	-0.2675	-3.7261	0.0148	0.4261	No	No PWM Hits	No PWM Hits
KH2012:KH.L171.10	Hex	-0.2895	-7.8105	-0.0173	-0.9582	No	No PWM match	No PWM match
KH2012:KH.S1032.1	TEF/HLF	-0.2999	-3.0810	0.0307	0.3747	No	No PWM Hits	No PWM Hits
KH2012:KH.C2.277	TAR DNA-binding protein	-0.3241	-4.0081	-0.0824	-0.5394	No	No PWM match	No PWM match
KH2012:KH.C12.676	PUR-α/β/γ	-0.3241	-3.2474	-0.0803	-0.5479	No	No PWM match	No PWM match
KH2012:KH.C4.182	Zinc Finger (C3H)	-0.3524	-5.9005	-0.0123	-0.1311	No	No PWM match	No PWM match
KH2012:KH.L96.11	Groucho2	-0.3640	-4.2613	-0.2353	-3.0840	No	No PWM match	No PWM match
KH2012:KH.C1.777	Zinc Finger (C2H2)-33	-0.3718	-4.6877	0.1354	2.1616	No	No PWM Hits	No PWM Hits
KH2012:KH.C4.83	HIF	-0.3878	-6.4649	-0.1010	-1.3534	No	No PWM match	No PWM match
KH2012:KH.C5.74	Orphan Fox-4	-0.4031	-9.3002	0.1101	1.5932	No	No PWM Hits	No PWM Hits
KH2012:KH.C4.366	ERF2	-0.4195	-3.7483	0.1896	3.1520	No	-11.04	-1.9995
KH2012:KH.C5.610	Jun	-0.4710	-11.6208	0.0220	1.3308	No	No PWM Hits	No PWM Hits
KH2012:KH.C7.294	Orphan bZIP-4	-0.5305	-18.3876	0.0000	0.0000	No	No PWM match	No PWM match
KH2012:KH.C7.523	SoxC	-0.5305	-11.7843	-0.0726	-0.9748	No	No PWM match	No PWM match
KH2012:KH.C1.159	E(spl)/hairy-a	-0.6719	-8.7455	-0.0356	-0.6665	No	No PWM Hits	No PWM Hits
KH2012:KH.C4.84	Otx	-0.6809	-11.3246	0.7148	17.6296	No	-1.891	-0.65
KH2012:KH.C4.539	Fli/ERG1	-0.9175	-24.9587	0.0000	0.0000	No	-18.5715	-1.9995
KH2012:KH.C11.314	Fos	-1.0507	-20.0931	0.3360	9.1940	No	No PWM Hits	No PWM Hits
KH2012:KH.C3.230	Smad6/7	-1.0790	-21.9317	0.2395	4.3007	No	No PWM match	No PWM match
KH2012:KH.C11.485	Irx-B	-1.1750	-18.2077	0.7777	5.9918	No	No PWM Hits	No PWM Hits
KH2012:KH.C5.202	TWIST-like-2	-1.1758	-32.1674	0.0000	0.0000	No	No PWM match	No PWM match
KH2012:KH.C10.148	Fli/ERG3	-1.2004	-28.4723	-0.0624	-1.7549	No	-17.572	-1.9995
KH2012:KH.C8.247	ELK	-2.2374	-31.9458	-0.0361	-0.0233	No	-11.571	-1.9995
KH2012:KH.C5.416	TWIST-like-1a/b	-2.7229	-42.8596	0.0220	1.3308	No	No PWM match	No PWM match

JASPAR 2020 PWM cluster	Max Z-score in cluster	Max Z-score in cluster
RREB1	24.52	RREB1
TBX21	19.72	TBX2
E2F7	19.61	E2F3
POU4F1	17.83	HNF1B
BHLHA15_var_2	17.01	Tcf12
TBX19	15.45	TBX19

B7.8 Muscle

KH2012 gene model	TF common name	Log2FC Sibling Cluster	-Log(pvalue) Sibling Cluster	Log2FC Parent Cluster	-Log(pvalue) Parent Cluster	DE in Imai	Matched PWM z-score	PWM Cluster z-score
KH2012:KH.S423.6	SMYD1	4.6723	34.5143	1.7951	42.7396	Not Analyzed	No PWM match	No PWM match
KH2012:KH.S654.3	Tbx6b/c/d	2.6344	34.3655	0.5967	10.7568	Not Analyzed	No PWM match	No PWM match
KH2012:KH.C14.307	MyoD	2.5380	33.4623	0.4570	8.2609	Not Analyzed	No PWM Hits	No PWM Hits
KH2012:KH.C14.377	Otp	2.2983	31.7226	0.2820	2.0260	Not Analyzed	No PWM Hits	No PWM Hits
KH2012:KH.L128.12	Mnx	1.8714	27.2031	1.5904	33.0597	Not Analyzed	1.5165	12.9155
KH2012:KH.C3.751	Snail	1.8648	30.8885	0.6203	16.3661	Not Analyzed	No PWM Hits	No PWM Hits
KH2012:KH.C1.264	Y-box protein1/2/3	1.6442	28.0455	1.6688	40.9592	Not Analyzed	No PWM match	No PWM match
KH2012:KH.C9.717	FoxH-a	1.6033	25.8387	1.6033	40.5922	Not Analyzed	4.706	8.4355
KH2012:KH.C3.17	ROR	1.5633	30.5716	1.3168	36.5184	Not Analyzed	8.7275	13.813
KH2012:KH.L96.87	Tbx2/3	1.0014	17.8988	0.5949	10.1186	Not Analyzed	19.5625	24.211
KH2012:KH.C9.580	RAR	0.9606	14.5356	0.8381	16.4476	Not Analyzed	2.807	5.667
KH2012:KH.L4.17	Zinc Finger (C2H2)-18	0.8893	12.5248	0.8546	16.7498	Not Analyzed	18.2245	18.2245
KH2012:KH.C3.312	E(spl)/hairy-b	0.8117	8.9093	0.3438	3.3563	Not Analyzed	No PWM Hits	No PWM Hits
KH2012:KH.C2.725	Su(H)/RBP-J	0.7019	8.6387	0.5652	7.7834	Not Analyzed	No PWM Hits	No PWM Hits
KH2012:KH.C4.83	HIF	0.6816	9.3136	0.6057	10.5330	Not Analyzed	No PWM match	No PWM match
KH2012:KH.C1.661	MAD	0.6551	8.4198	0.2988	3.1180	Not Analyzed	No PWM match	No PWM match
KH2012:KH.C10.174	meis	0.5850	9.0677	0.5850	14.9679	Not Analyzed	7.704	9.51
KH2012:KH.C1.669	Zinc Finger (C2H2)-25	0.5423	6.0446	0.5302	8.0374	Not Analyzed	0.627	0.627
KH2012:KH.C3.84	HNF4	0.5200	6.2429	0.2928	3.1660	Not Analyzed	No PWM Hits	No PWM Hits
KH2012:KH.C3.348	E12/E47	0.5043	7.7492	0.3915	6.9233	Not Analyzed	No PWM Hits	No PWM Hits
KH2012:KH.L13.24	FoxN2/3	0.5017	5.7233	0.4670	6.4587	Not Analyzed	-8.067	1.6595
KH2012:KH.C12.676	PUR-α/β/γ	0.4820	6.8694	0.0531	0.4761	Not Analyzed	No PWM match	No PWM match
KH2012:KH.C3.103	RBP1-like	0.4397	9.4453	-0.0502	-0.7320	Not Analyzed	No PWM match	No PWM match
KH2012:KH.S980.1	Zinc finger (PHD)-2	0.4211	3.9609	-0.2020	-2.3590	Not Analyzed	No PWM match	No PWM match
KH2012:KH.S899.4	BTF3	0.4097	6.1733	0.6200	17.4324	Not Analyzed	No PWM match	No PWM match
KH2012:KH.C14.448	AP4	0.3870	7.5317	0.2395	4.3825	Not Analyzed	26.114	40.8775
KH2012:KH.C14.449	CBP	0.3659	3.4675	-0.0417	-0.6856	Not Analyzed	No PWM match	No PWM match
KH2012:KH.C4.810	HSF	0.3617	6.4693	0.0560	1.1022	Not Analyzed	No PWM match	No PWM match
KH2012:KH.C3.230	Smad6/7	0.3357	3.5591	0.1991	2.0509	Not Analyzed	No PWM match	No PWM match
KH2012:KH.C4.504	FoxM	0.3093	5.0423	-0.3155	-4.8077	Not Analyzed	-8.067	1.6595
KH2012:KH.C1.983	pem4	0.3033	5.0846	0.1964	3.7127	Not Analyzed	-2.321	10.051
KH2012:KH.C9.38	FUBP	0.2939	3.3449	0.2801	3.6347	Not Analyzed	No PWM match	No PWM match
KH2012:KH.S1032.1	TEF/HLF	0.2845	4.0384	0.1104	1.2890	Not Analyzed	No PWM Hits	No PWM Hits
KH2012:KH.C4.509	Zinc Finger (absent, small, or homeotic discs 1)	0.2768	3.3736	-0.2086	-1.8874	Not Analyzed	No PWM match	No PWM match
KH2012:KH.C2.961	Zinc Finger (C2H2)-12	0.2701	3.8786	-0.2518	-1.8329	Not Analyzed	0.275	0.275
KH2012:KH.C7.523	SoxC	0.2520	3.5484	0.3012	4.8053	Not Analyzed	No PWM match	No PWM match
KH2012:KH.C3.83	Six45	-0.2817	-4.8001	-0.0666	-1.0748	Not Analyzed	No PWM Hits	No PWM Hits
KH2012:KH.L153.50	HsignallingT1; suppressor of S. cerevisiae gcr2	-0.2860	-3.4880	0.1172	0.9807	Not Analyzed	No PWM match	No PWM match
KH2012:KH.C1.1271	AP-2-like1	-0.3081	-5.0641	-0.1326	-1.8371	Not Analyzed	No PWM match	No PWM match
KH2012:KH.C3.308	Mist	-0.3301	-6.1292	0.0000	0.0000	Not Analyzed	3.5065	40.8775
KH2012:KH.L60.12	transcription factor Dp-1	-0.3536	-3.1603	-0.2630	-2.8749	Not Analyzed	No PWM match	No PWM match
KH2012:KH.L155.15	SWI/SNF	-0.3785	-4.0302	-0.3137	-3.0805	Not Analyzed	No PWM match	No PWM match
KH2012:KH.C7.111	Transcription initiation protein SPT4 homolog	-0.3879	-4.7303	-0.5392	-6.8707	Not Analyzed	No PWM match	No PWM match
KH2012:KH.C11.313	FoxA-a	-0.4253	-7.0538	-0.0437	-1.5687	Not Analyzed	No PWM Hits	No PWM Hits
KH2012:KH.L171.10	Hex	-0.4253	-10.9308	0.0000	0.0000	Not Analyzed	No PWM match	No PWM match
KH2012:KH.L96.11	Groucho2	-0.4268	-5.1105	-0.2767	-3.0120	Not Analyzed	No PWM match	No PWM match
KH2012:KH.L9.33	E(Y)2	-0.4351	-4.1855	-0.3471	-4.9038	Not Analyzed	No PWM match	No PWM match
KH2012:KH.C11.535	Zinc Finger (C2H2)-17	-0.4353	-3.9005	-0.4312	-6.1301	Not Analyzed	No PWM match	No PWM match
KH2012:KH.C5.90	core-binding factor, β subunit	-0.4509	-5.3418	-0.3441	-5.0303	Not Analyzed	No PWM match	No PWM match
KH2012:KH.C7.702	Zinc Finger (BTB/POZ)	-0.4871	-5.2491	-0.9388	-20.6330	Not Analyzed	No PWM Hits	No PWM Hits
KH2012:KH.C5.373	Max	-0.5163	-6.0225	-0.5427	-8.2038	Not Analyzed	-5.0485	9.4235
KH2012:KH.C14.306	IRF-like 2	-0.5174	-6.8222	-0.5493	-9.8164	Not Analyzed	No PWM match	No PWM match
KH2012:KH.S1482.3	NFRKB	-0.5327	-6.8842	-0.3128	-4.1658	Not Analyzed	No PWM match	No PWM match
KH2012:KH.C11.314	Fos	-0.5461	-5.7262	-0.2464	-3.5309	Not Analyzed	No PWM Hits	No PWM Hits
KH2012:KH.C10.245	AF10like	-0.5514	-5.3721	-0.4671	-9.3029	Not Analyzed	No PWM match	No PWM match
KH2012:KH.C2.573	Smad1/5	-0.5712	-8.0388	-0.4338	-7.0051	Not Analyzed	No PWM Hits	No PWM Hits
KH2012:KH.C11.620	ubiquitin-like, containing PHD and RING finger domains, 1	-0.5773	-14.8023	-0.8100	-27.1839	Not Analyzed	No PWM match	No PWM match
KH2012:KH.C14.178	HMG1/2	-0.5778	-13.1187	-0.1039	-1.6293	Not Analyzed	No PWM match	No PWM match
KH2012:KH.C1.516	HMG20	-0.6372	-7.3702	-0.3854	-4.3808	Not Analyzed	No PWM match	No PWM match
KH2012:KH.C8.247	ELK	-0.6737	-6.4131	0.2919	2.5872	Not Analyzed	0.1595	8.3165
KH2012:KH.L41.71	Zinc Finger (C2H2)-32	-0.6857	-7.1369	-0.6721	-12.1675	Not Analyzed	No PWM match	No PWM match
KH2012:KH.C9.892	RXR	-0.6985	-8.0205	-0.2556	-3.4127	Not Analyzed	-3.16	7.438
KH2012:KH.C2.822	E2F1/2/3/6	-0.7287	-9.1479	-0.9005	-17.3287	Not Analyzed	No PWM match	No PWM match
KH2012:KH.C14.278	Zinc Finger (C2H2)-34	-0.8723	-11.3701	-1.0556	-26.2417	Not Analyzed	No PWM Hits	No PWM Hits
KH2012:KH.C10.148	Fli/ERG3	-1.1186	-15.0226	-0.0220	-0.9666	Not Analyzed	-3.039	8.3165
KH2012:KH.C4.84	Otx	-1.1897	-9.5771	-1.8202	-36.3566	Not Analyzed	2.969	8.4355
KH2012:KH.C4.539	Fli/ERG1	-1.2630	-23.1714	0.0000	0.0000	Not Analyzed	-0.9995	8.3165
KH2012:KH.C4.182	Zinc Finger (C3H)	-1.4726	-27.1960	0.0055	0.1239	Not Analyzed	No PWM match	No PWM match
KH2012:KH.C5.202	TWIST-like-2	-1.9041	-24.3872	0.0000	0.0000	Not Analyzed	No PWM match	No PWM match
KH2012:KH.C5.416	TWIST-like-1a/b	-2.5001	-31.0997	-0.0220	-0.9666	Not Analyzed	No PWM match	No PWM match

JASPAR 2020 PWM cluster	Max Z-score in cluster	Max Z-score in cluster
BHLHA15_var_2	40.88	MYOG
TBX21	24.21	MGA
Zic1::Zic2	23.78	ZNF317
FOSL2::JUNB_var_2	21.80	ATF7
Rhox11	21.09	ZNF341
MTF1	18.33	MTF1
RREB1	18.22	RREB1
TBX19	16.58	TBX19
GLIS2	16.07	ZIC5
Gmeb1	16.01	Gmeb1

B7.7

KH2012 gene model	TF common name	Log2FC Sibling Cluster	-Log(pvalue) Sibling Cluster	DE in Imai	Matched PWM z-score	PWM Cluster z-score
KH2012:KH.C11.485	Irx-B	0.5107	8.7520	No	No PWM Hits	No PWM Hits
KH2012:KH.C5.416	TWIST-like-1a/b	0.4371	12.8343	Yes	No PWM match	No PWM match
KH2012:KH.C8.247	ELK	0.3124	5.2856	Yes	-2.588	8.4965
KH2012:KH.L18.112	Zinc finger (PHD/NFX, DDT)	0.2934	3.4130	No	No PWM match	No PWM match
KH2012:KH.C5.130	Zinc Finger (C2H2)-27	0.2562	5.2742	No	No PWM Hits	No PWM Hits
KH2012:KH.C14.520	Orphan Fox-1	-0.2845	-8.6015	No	No PWM Hits	No PWM Hits
KH2012:KH.C14.377	Otp	-0.2863	-12.6843	No	No PWM Hits	No PWM Hits
KH2012:KH.S423.6	SMYD1	-0.3004	-10.8801	No	No PWM match	No PWM match
KH2012:KH.S654.3	Tbx6b/c/d	-0.4464	-11.7331	No	No PWM match	No PWM match
KH2012:KH.C3.751	Snail	-0.5435	-10.6236	No	No PWM Hits	No PWM Hits
KH2012:KH.C3.773	Ci-mT	-0.6625	-16.9725	No	No PWM match	No PWM match
KH2012:KH.C14.307	MyoD	-0.6898	-27.3493	No	No PWM Hits	No PWM Hits

JASPAR 2020 PWM cluster	Max Z-score in cluster	Max Z-score in cluster
NFKB2	22.49	NFKB2
Nr2f6	16.05	Pparg::Rxra

B7.7

KH2012 gene model	TF common name	Log2FC Sibling Cluster	-Log(pvalue) Sibling Cluster	Log2FC Parent Cluster	-Log(pvalue) Parent Cluster	DE in Imai	Matched PWM z-score	PWM Cluster z-score
KH2012:KH.C5.416	TWIST-like-1a/b	2.7229	42.8596	2.3079	40.8247	Yes	No PWM match	No PWM match
KH2012:KH.C8.247	ELK	2.2374	31.9458	1.8890	34.1960	Yes	18.087	27.736
KH2012:KH.C10.148	Fli/ERG3	1.2004	28.4723	1.1155	34.8525	Yes	26.667	27.736
KH2012:KH.C5.202	TWIST-like-2	1.1758	32.1674	1.1758	57.0832	No	No PWM match	No PWM match
KH2012:KH.C11.485	Irx-B	1.1750	18.2077	1.4421	29.5662	Yes	No PWM Hits	No PWM Hits
KH2012:KH.C3.230	Smad6/7	1.0790	21.9317	1.2425	33.9879	Yes	No PWM match	No PWM match
KH2012:KH.C11.314	Fos	1.0507	20.0931	1.3895	55.6499	Yes	No PWM Hits	No PWM Hits
KH2012:KH.C4.539	Fli/ERG1	0.9175	24.9587	0.9065	41.8765	Yes	27.736	27.736
KH2012:KH.C4.84	Otx	0.6809	11.3246	1.5820	30.7827	No	2.363	12.9165
KH2012:KH.C1.159	E(spl)/hairy-a	0.6719	8.7455	0.6719	12.8827	No	No PWM Hits	No PWM Hits
KH2012:KH.C7.523	SoxC	0.5305	11.7843	0.3469	6.4397	Yes	No PWM match	No PWM match
KH2012:KH.C7.294	Orphan bZIP-4	0.5305	18.3876	0.5085	29.6572	Yes	No PWM match	No PWM match
KH2012:KH.C5.610	Jun	0.4710	11.6208	0.4820	21.8590	Yes	No PWM Hits	No PWM Hits
KH2012:KH.C4.366	ERF2	0.4195	3.7483	0.7585	11.9824	Yes	17.684	27.736
KH2012:KH.C5.74	Orphan Fox-4	0.4031	9.3002	0.4976	19.8048	No	No PWM Hits	No PWM Hits
KH2012:KH.C4.83	HIF	0.3878	6.4649	0.3339	6.3871	No	No PWM match	No PWM match
KH2012:KH.C1.777	Zinc Finger (C2H2)-33	0.3718	4.6877	0.5440	16.5442	No	No PWM Hits	No PWM Hits
KH2012:KH.L96.11	Groucho2	0.3640	4.2613	-0.0270	-0.0645	No	No PWM match	No PWM match
KH2012:KH.C4.182	Zinc Finger (C3H)	0.3524	5.9005	0.3744	14.1773	No	No PWM match	No PWM match
KH2012:KH.C12.676	PUR-α/β/γ	0.3241	3.2474	0.0491	0.7292	No	No PWM match	No PWM match
KH2012:KH.C2.277	TAR DNA-binding protein	0.3241	4.0081	0.3415	5.4815	No	No PWM match	No PWM match
KH2012:KH.S1032.1	TEF/HLF	0.2999	3.0810	0.3930	6.0716	No	No PWM Hits	No PWM Hits
KH2012:KH.L171.10	Hex	0.2895	7.8105	0.2895	14.1786	No	No PWM match	No PWM match
KH2012:KH.C2.161	FXR	0.2675	3.7261	0.3185	8.2486	No	No PWM Hits	No PWM Hits
KH2012:KH.C14.306	IRF-like 2	-0.2609	-3.0892	-0.1354	-0.7540	No	No PWM match	No PWM match
KH2012:KH.C9.580	RAR	-0.2717	-3.1355	-0.3250	-3.0811	No	11.843	11.843
KH2012:KH.C3.697	Zinc Finger (C2H2)-5	-0.2944	-3.0427	0.1258	0.2395	No	No PWM Hits	No PWM Hits
KH2012:KH.L39.1	FoxH-b	-0.2996	-3.7250	-0.3699	-5.3016	No	0.6735	12.9165
KH2012:KH.C11.450	CBF	-0.3853	-4.3658	-0.2823	-2.9844	No	No PWM match	No PWM match
KH2012:KH.L41.39	Orphan bHLH-4	-0.3981	-3.7886	0.0433	0.2650	No	No PWM match	No PWM match
KH2012:KH.L128.12	Mnx	-0.4371	-3.4179	-0.0111	-0.4144	No	-6.462	4.241
KH2012:KH.C3.17	ROR	-0.4458	-4.9430	0.2844	3.7625	No	-3.9795	7.673
KH2012:KH.C14.520	Orphan Fox-1	-0.4920	-6.4783	-0.3137	-3.7505	No	No PWM Hits	No PWM Hits
KH2012:KH.C1.661	MAD	-0.5399	-6.1518	-0.2279	-1.4712	No	No PWM match	No PWM match
KH2012:KH.L4.17	Zinc Finger (C2H2)-18	-0.8329	-7.7609	-0.0446	-1.2658	No	-16.0145	-16.0145
KH2012:KH.C3.773	Ci-mT	-0.8429	-8.0740	-0.8391	-9.7029	No	No PWM match	No PWM match
KH2012:KH.S654.3	Tbx6b/c/d	-1.4002	-22.0302	0.2086	2.9783	No	No PWM match	No PWM match
KH2012:KH.C14.307	MyoD	-1.9778	-27.1326	0.0594	0.4004	No	No PWM Hits	No PWM Hits
KH2012:KH.C14.377	Otp	-2.0204	-30.8285	0.1409	6.1967	No	No PWM Hits	No PWM Hits
KH2012:KH.S423.6	SMYD1	-3.4785	-31.1062	0.1409	3.8031	No	No PWM match	No PWM match

JASPAR 2020 PWM cluster	Max Z-score in cluster	Max Z-score in cluster
ELK3	27.74	ERG
GCM2	17.93	CENPB
NFKB2	15.10	REL

B7.7 Mesenchyme

KH2012 gene model	TF common name	Log2FC Sibling Cluster	-Log(pvalue) Sibling Cluster	Log2FC Parent Cluster	-Log(pvalue) Parent Cluster	DE in Imai	Matched PWM z-score	PWM Cluster z-score
KH2012:KH.C5.416	TWIST-like-1a/b	2.5001	31.0997	-0.2449	-1.3490	Not Analyzed	No PWM match	No PWM match
KH2012:KH.C5.202	TWIST-like-2	1.9041	24.3872	0.7283	1.3891	Not Analyzed	No PWM match	No PWM match
KH2012:KH.C4.182	Zinc Finger (C3H)	1.4726	27.1960	1.1257	11.7259	Not Analyzed	No PWM match	No PWM match
KH2012:KH.C4.539	Fli/ERG1	1.2630	23.1714	0.3455	1.7897	Not Analyzed	0.7645	9.248
KH2012:KH.C4.84	Otx	1.1897	9.5771	-1.3113	-16.0493	Not Analyzed	-3.7365	1.844
KH2012:KH.C10.148	Fli/ERG3	1.1186	15.0226	-0.1037	-1.5336	Not Analyzed	3.299	9.248
KH2012:KH.C14.278	Zinc Finger (C2H2)-34	0.8723	11.3701	-0.2546	-2.0026	Not Analyzed	No PWM Hits	No PWM Hits
KH2012:KH.C2.822	E2F1/2/3/6	0.7287	9.1479	-0.1603	-1.0392	Not Analyzed	No PWM match	No PWM match
KH2012:KH.C9.892	RXR	0.6985	8.0205	0.2518	0.6653	Not Analyzed	3.863	9.328
KH2012:KH.L41.71	Zinc Finger (C2H2)-32	0.6857	7.1369	-0.0275	-0.3310	Not Analyzed	No PWM match	No PWM match
KH2012:KH.C8.247	ELK	0.6737	6.4131	-1.2718	-16.5537	Not Analyzed	-0.5085	9.248
KH2012:KH.C1.516	HMG20	0.6372	7.3702	0.2160	1.6217	Not Analyzed	No PWM match	No PWM match
KH2012:KH.C14.178	HMG1/2	0.5778	13.1187	0.4051	5.6307	Not Analyzed	No PWM match	No PWM match
KH2012:KH.C11.620	ubiquitin-like, containing PHD and RING finger domains, 1	0.5773	14.8023	-0.0692	-0.0713	Not Analyzed	No PWM match	No PWM match
KH2012:KH.C2.573	Smad1/5	0.5712	8.0388	0.1301	0.9399	Not Analyzed	No PWM Hits	No PWM Hits
KH2012:KH.C10.245	AF10like	0.5514	5.3721	-0.1487	-1.9395	Not Analyzed	No PWM match	No PWM match
KH2012:KH.C11.314	Fos	0.5461	5.7262	-0.7510	-10.1808	Not Analyzed	No PWM Hits	No PWM Hits
KH2012:KH.S1482.3	NFRKB	0.5327	6.8842	0.1912	1.5938	Not Analyzed	No PWM match	No PWM match
KH2012:KH.C14.306	IRF-like 2	0.5174	6.8222	0.2289	2.3771	Not Analyzed	No PWM match	No PWM match
KH2012:KH.C5.373	Max	0.5163	6.0225	-0.1046	-0.7640	Not Analyzed	6.063	12.789
KH2012:KH.C7.702	Zinc Finger (BTB/POZ)	0.4871	5.2491	-0.4585	-5.2756	Not Analyzed	No PWM Hits	No PWM Hits
KH2012:KH.C5.90	core-binding factor, β subunit	0.4509	5.3418	0.0617	0.2783	Not Analyzed	No PWM match	No PWM match
KH2012:KH.C11.535	Zinc Finger (C2H2)-17	0.4353	3.9005	0.2930	1.4709	Not Analyzed	No PWM match	No PWM match
KH2012:KH.L9.33	E(Y)2	0.4351	4.1855	-0.1520	-1.1102	Not Analyzed	No PWM match	No PWM match
KH2012:KH.L96.11	Groucho2	0.4268	5.1105	-0.2139	-1.5857	Not Analyzed	No PWM match	No PWM match
KH2012:KH.C11.313	FoxA-a	0.4253	7.0538	0.4253	4.1924	Not Analyzed	No PWM Hits	No PWM Hits
KH2012:KH.L171.10	Hex	0.4253	10.9308	0.1358	1.2593	Not Analyzed	No PWM match	No PWM match
KH2012:KH.C7.111	Transcription initiation protein SPT4 homolog	0.3879	4.7303	-0.3893	-4.3047	Not Analyzed	No PWM match	No PWM match
KH2012:KH.L155.15	SWI/SNF	0.3785	4.0302	-0.0451	-0.1102	Not Analyzed	No PWM match	No PWM match
KH2012:KH.L60.12	transcription factor Dp-1	0.3536	3.1603	-0.1380	-1.1473	Not Analyzed	No PWM match	No PWM match
KH2012:KH.C3.308	Mist	0.3301	6.1292	0.2271	1.4008	Not Analyzed	-4.4575	17.4915
KH2012:KH.C1.1271	AP-2-like1	0.3081	5.0641	0.2595	2.3256	Not Analyzed	No PWM match	No PWM match
KH2012:KH.L153.50	HsignallingT1; suppressor of S. cerevisiae gcr2	0.2860	3.4880	0.2958	2.4805	Not Analyzed	No PWM match	No PWM match
KH2012:KH.C3.83	Six45	0.2817	4.8001	0.3101	3.8798	Not Analyzed	No PWM Hits	No PWM Hits
KH2012:KH.C7.523	SoxC	-0.2520	-3.5484	-0.4813	-8.0143	Not Analyzed	No PWM match	No PWM match
KH2012:KH.C2.961	Zinc Finger (C2H2)-12	-0.2701	-3.8786	-0.5968	-7.1100	Not Analyzed	-0.3325	-0.3325
KH2012:KH.C4.509	Zinc Finger (absent, small, or homeotic discs 1)	-0.2768	-3.3736	-0.3591	-2.5508	Not Analyzed	No PWM match	No PWM match
KH2012:KH.S1032.1	TEF/HLF	-0.2845	-4.0384	-0.4739	-5.6310	Not Analyzed	No PWM Hits	No PWM Hits
KH2012:KH.C9.38	FUBP	-0.2939	-3.3449	-0.1216	-0.8511	Not Analyzed	No PWM match	No PWM match
KH2012:KH.C1.983	pem4	-0.3033	-5.0846	-0.1114	-0.8875	Not Analyzed	3.201	9.0425
KH2012:KH.C4.504	FoxM	-0.3093	-5.0423	-0.4385	-3.3170	Not Analyzed	10.0105	13.936
KH2012:KH.C3.230	Smad6/7	-0.3357	-3.5591	-1.2157	-19.2505	Not Analyzed	No PWM match	No PWM match
KH2012:KH.C4.810	HSF	-0.3617	-6.4693	-0.3744	-3.6005	Not Analyzed	No PWM match	No PWM match
KH2012:KH.C14.449	CBP	-0.3659	-3.4675	-0.3388	-3.2232	Not Analyzed	No PWM match	No PWM match
KH2012:KH.C14.448	AP4	-0.3870	-7.5317	-0.2895	-5.5809	Not Analyzed	-22.4505	17.4915
KH2012:KH.S899.4	BTF3	-0.4097	-6.1733	0.1917	1.5758	Not Analyzed	No PWM match	No PWM match
KH2012:KH.S980.1	Zinc finger (PHD)-2	-0.4211	-3.9609	-0.5357	-3.1605	Not Analyzed	No PWM match	No PWM match
KH2012:KH.C3.103	RBP1-like	-0.4397	-9.4453	-0.2613	-1.8826	Not Analyzed	No PWM match	No PWM match
KH2012:KH.C12.676	PUR-α/β/γ	-0.4820	-6.8694	-0.7529	-10.1270	Not Analyzed	No PWM match	No PWM match
KH2012:KH.L13.24	FoxN2/3	-0.5017	-5.7233	-0.2360	-2.6921	Not Analyzed	10.0105	13.936
KH2012:KH.C3.348	E12/E47	-0.5043	-7.7492	-0.0254	-0.3485	Not Analyzed	No PWM Hits	No PWM Hits
KH2012:KH.C3.84	HNF4	-0.5200	-6.2429	-0.2771	-1.8572	Not Analyzed	No PWM Hits	No PWM Hits
KH2012:KH.C1.669	Zinc Finger (C2H2)-25	-0.5423	-6.0446	0.0850	0.3648	Not Analyzed	-0.362	-0.362
KH2012:KH.C10.174	meis	-0.5850	-9.0677	0.0000	0.0000	Not Analyzed	-9.434	-8.521
KH2012:KH.C1.661	MAD	-0.6551	-8.4198	0.1836	0.8534	Not Analyzed	No PWM match	No PWM match
KH2012:KH.C4.83	HIF	-0.6816	-9.3136	-0.4637	-6.8708	Not Analyzed	No PWM match	No PWM match
KH2012:KH.C2.725	Su(H)/RBP-J	-0.7019	-8.6387	-0.2813	-2.4332	Not Analyzed	No PWM Hits	No PWM Hits
KH2012:KH.C3.312	E(spl)/hairy-b	-0.8117	-8.9093	-0.2566	-3.2373	Not Analyzed	No PWM Hits	No PWM Hits
KH2012:KH.L4.17	Zinc Finger (C2H2)-18	-0.8893	-12.5248	0.7982	5.0368	Not Analyzed	-10.099	-10.099
KH2012:KH.C9.580	RAR	-0.9606	-14.5356	0.1492	1.4702	Not Analyzed	-2.894	-2.894
KH2012:KH.L96.87	Tbx2/3	-1.0014	-17.8988	-0.5484	-8.4925	Not Analyzed	-18.937	-4.11
KH2012:KH.C3.17	ROR	-1.5633	-30.5716	0.1993	1.5701	Not Analyzed	-8.4075	4.57
KH2012:KH.C9.717	FoxH-a	-1.6033	-25.8387	0.0000	0.0000	Not Analyzed	-5.0295	1.844
KH2012:KH.C1.264	Y-box protein1/2/3	-1.6442	-28.0455	0.2606	0.9676	Not Analyzed	No PWM match	No PWM match
KH2012:KH.C3.751	Snail	-1.8648	-30.8885	-1.2135	-13.7894	Not Analyzed	No PWM Hits	No PWM Hits
KH2012:KH.L128.12	Mnx	-1.8714	-27.2031	0.1561	2.0661	Not Analyzed	-0.46	16.88
KH2012:KH.C14.377	Otp	-2.2983	-31.7226	0.0041	0.6769	Not Analyzed	No PWM Hits	No PWM Hits
KH2012:KH.C14.307	MyoD	-2.5380	-33.4623	-0.1031	-1.3141	Not Analyzed	No PWM Hits	No PWM Hits
KH2012:KH.S654.3	Tbx6b/c/d	-2.6344	-34.3655	-0.6374	-10.1111	Not Analyzed	No PWM match	No PWM match
KH2012:KH.S423.6	SMYD1	-4.6723	-34.5143	0.6014	1.4597	Not Analyzed	No PWM match	No PWM match

JASPAR 2020 PWM cluster	Max Z-score in cluster	Max Z-score in cluster
SRF	30.18	SRF
FOS::JUNB	25.50	Nfe2l2
ZNF384	21.51	ZNF384
ESR1	20.09	ESR1
Stat5a::Stat5b	18.79	STAT3
BHLHA15_var_2	17.49	Twist2
MEF2D	17.34	MEF2C
PDX1	16.88	Dlx1
NFKB2	16.06	REL
YY1	15.75	YY1
Plagl1	15.57	Plagl1

0.04
